# CerM and Its Antagonist CerN Are New Components of the Quorum Sensing System in *Cereibacter sphaeroides*, Signaling to the CckA/ChpT/CtrA System

**DOI:** 10.1002/mbo3.70012

**Published:** 2024-12-18

**Authors:** José Hernández‐Valle, Benjamín Vega‐Baray, Sebastián Poggio, Laura Camarena

**Affiliations:** ^1^ Departamento de Biología Molecular y Biotecnología, Instituto de Investigaciones Biomédicas Universidad Nacional Autónoma de México Mexico City Mexico

**Keywords:** CckA, CerI, CtrA, LuxR, quorum sensing, TrlR, two‐component system

## Abstract

*Cereibacter sphaeroides* has a quorum sensing (QS) system that has been partially characterized. Using a bioinformatic approach, six LuxR homologs and one homolog of the acylhomoserine lactone synthase were identified in this bacterium, including the previously characterized CerR and CerI proteins. This study focused on determining the roles of two LuxR homologs, CerM and CerN. CerN lacks the HTH domain and, together with CerM, controls the expression of ctrA, which is part of the TCS CckA/ChpT/CtrA. CtrA is widely conserved in alpha‐proteobacteria and regulates flagellar motility and other cellular processes. Genetic and biochemical data suggest that CerM indirectly represses *ctrA* expression, which is counteracted by its interaction with CerN‐AHL. A transcriptomic study identified 181 genes regulated by CerM/CerN, with a conserved sequence in their regulatory regions likely indicating the CerM binding site. This hypothesis was supported by in vitro and in vivo DNA–protein interaction assays. Our results identified a transcription factor that could connect the QS system with the regulation of the two‐component system CckA/ChpT/CtrA.

## Graphical abstract

The quorum sensing (QS) system of *Cereibacter sphaeroides* includes six LuxR homologs and one acylhomoserine lactone synthase homolog. Two LuxR homologs, CerM and CerN, are involved in regulating the expression of ctrA, which is part of the CckA/ChpT/CtrA two‐component system that controls flagellar motility. This study indicates that CerN‐AHL counteracts CerM's repression of ctrA, thereby connecting the QS system with the regulation by the two‐component system.

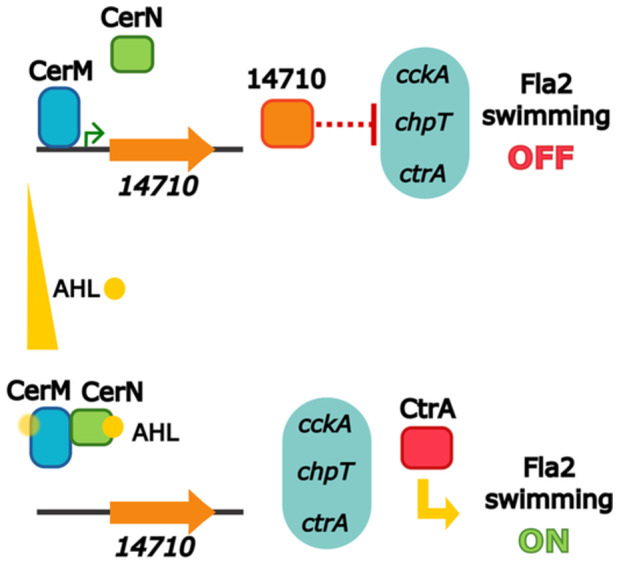

## 1 Introduction

The quorum sensing (QS) system enables bacteria to control gene expression based on cell density. In *Aliivibrio fischeri*, the synthase LuxI catalyzes the production of the autoinducer 3‐oxo‐hexanoyl homoserine lactone (3‐oxo‐C6‐HSL), which is continuously produced and released to the extracellular space. As the population proliferates, there is a continuous accumulation of this signal molecule. Once a specific concentration threshold is reached, it binds to the transcriptional regulator LuxR, and this complex activates the expression of *lux* genes required to produce luminescence (Fuqua and Greenberg 2002; Miller and Bassler 2001). In this bacterium, the QS system also regulates swimming motility (Lupp and Ruby 2005) and biofilm formation (Ray and Visick 2012), coordinating these behaviors with changes in population density.

In species of the Roseobacteraceae and Paracoccaceae families, the QS systems are predominantly composed of LuxR and LuxI homologs (Liang et al. 2021; Zan et al. 2014), and for several microorganisms, duplications or even multiplications of the acylhomoserine lactone (AHL) synthases and the LuxR regulators are frequently observed. This complexity includes not only situations where different AHLs are synthesized to control specific responses but also interconnections between these regulators in hierarchical patterns of expression or signaling toward other regulatory networks. In several microorganisms, traits such as flagellar motility, biofilm formation, conjugation, and production of the gene transfer agents (GTAs) are controlled by the two‐component system (TCS) CcKA/ChpT/CtrA. In many species, it has been determined that activation of the QS response regulates *ctrA* expression; however, the evidence so far suggests that this control is indirect.

The TCS formed by the hybrid histidine kinase CckA, the phosphotransfer protein ChpT, and the response regulator CtrA is widely distributed in α‐proteobacteria (Brilli et al. 2010) and has been thoroughly characterized in *Caulobacter crescentus*, where it is essential and controls DNA replication and cell cycle progression (Leicht et al. 2020; Quon et al. 1998; Quon, Marczynski, and Shapiro 1996). In contrast, in Rhodobacterales this TCS is not essential, with CtrA directly controlling flagellar biosynthesis and GTA production (Brilli et al. 2010; Lang and Beatty 2002; Mercer et al. 2010). In bacteria from this phylogenetic order, the relationship between the QS system and the TCS CckA/ChpT/CtrA has been reported in *Rhodobacter capsulatus*, *Ruegeria* sp. KLH11, and *Dinoroseobacter shibae* (Koppenhöfer et al. 2019; Leung, Brimacombe, and Beatty 2013; Zan et al. 2013). For these bacteria, it has been observed that in the absence of AHL—the expression of *ctrA* is downregulated (Leung, Brimacombe, and Beatty 2013; Patzelt et al. 2013; Zan et al. 2013). In *D*. *shibae*, the QS system consists of three AHL synthases associated with a *luxR* homolog, the genes *luxI1*‐*luxR1*, *luxI2*‐*luxR2*, and *luxI3*‐*luxB* (*luxB* is a *luxR*‐type regulator) (Patzelt et al. 2013; Wagner‐Döbler et al. 2010, 2005), as well as three orphan *luxR* homologs (*luxR3*‐*5*) (Wagner‐Döbler et al. 2010). It has been shown that LuxI1 is critical to promoting the expression of *ctrA*, which in turn activates the expression of *luxI2R2*, *luxI3*, and *luxB* (Patzelt et al. 2013; Wang et al. 2014).

In *R. capsulatus*, the QS system consists of the LuxR and LuxI homologs, GtaR and GtaI (Leung et al. 2012; Schaefer et al. 2002); it has been observed that in the absence of its autoinducer, GtaR represses, directly or indirectly, the expression of *ctrA*. This repression is relieved in the double mutant *gtaRI* (Leung, Brimacombe, and Beatty 2013). In *Ruegeria* sp. KLH11, the QS system consists of two pairs of *luxR* and *luxI* homologs, that is, *ssaRI* and *ssbRI*, and one orphan *luxI* homolog, named *sscI* (Zan et al. 2015). Flagellar motility is mainly controlled by SsaR and SsaI, and it was demonstrated that both proteins indirectly control the expression of *ctrA* through an unknown intermediary regulator (Zan et al. 2013, 2012).

Within Paracoccaceae, *Cereibacter sphaeroides* is another species in which the QS system has been studied. In this microorganism, the presence of the autoinducer synthase CerI that produces 7,8‐cis‐*N*‐(tetradecanoyl) homoserine lactone (C14‐HSL) and the regulator CerR, a homolog of LuxR was identified. The absence of these proteins leads to cell aggregation, hence the name given to these genes, which stands for *community escape response* (Puskas et al. 1997). Later, it was demonstrated that in cell cultures, the amount of AHL reaches a maximum during the exponential phase of growth, mirroring the transcriptional activity of *cerI* that is dependent on CerR (McIntosh et al. 2019).

An important trait controlled by the QS system is flagellar motility (Hochstrasser and Hilbi 2020; Jang et al. 2014; Koppenhöfer et al. 2019; Zan et al. 2012). *C. sphaeroides* represents an interesting model to study the mechanisms that control this phenotype since it carries two different flagellar systems of different phylogenic origins that are controlled by independent sets of regulatory proteins (Camarena and Dreyfus 2020). The flagellar *fla1* genes were acquired by *C. sphaeroides* through a horizontal transfer event, probably from an ancestral γ‐proteobacteria. These genes are constitutively expressed under the growth conditions commonly used in the laboratory, and their products assemble a single subpolar flagellum. The *fla2* genes represent the vertically inherited system characteristic of the α‐proteobacteria; however, the wild‐type strain does not show a significant expression of these genes (Poggio et al. 2007). It was demonstrated that the expression of the *fla2* genes was dependent on the activation of the TCS CckA/ChpT/CtrA, and their study is done using gain of function mutants of the CckA protein (Rivera‐Osorio et al. 2018; Vega‐Baray et al. 2022, 2015). The response regulator CtrA controls the expression of 321 genes, including the *fla2* genes as well as the chemotactic genes that control Fla2 rotation (Hernández‐Valle et al. 2020).

In this work, we characterized two novel LuxR homologs, CerM and CerN, which indirectly control the expression of the TCS CckA/ChpT/CtrA. Genetic and biochemical evidence support the idea that the expression of *ctrA* is indirectly regulated by these proteins. Among the genes directly controlled by CerM/CerN, we identified a transcription factor that could interconnect the QS system and the TCS CckA/ChpT/CtrA. Importantly, our results reveal a plausible mechanism to explain how CerN, a truncated LuxR regulator lacking the DNA‐binding domain, negatively affects the DNA‐binding ability of CerM in response to AHL.

## 2 Materials and Methods

### 2.1 Bacterial Strains, Plasmids, and Growth Conditions

Bacterial strains and plasmids used in this work are listed in Table 1. Oligonucleotides were purchased from Oligo T4 (Irapuato, Guanajuato, Mexico) and are listed in Table S1. *C*. *sphaeroides* WS8N strains were routinely grown in Sistrom's minimal medium without casamino acids (Hernández‐Valle et al. 2020; Sistrom 1962). This medium contains 15 mM succinic acid as the carbon source. When indicated, 0.2% casamino acids were used as the carbon source by supplementing a modified Sistrom's minimal medium that lacked succinic acid. Liquid cultures grown heterotrophically were incubated in the dark with orbital shaking at 200 rpm. Photoheterotrophic liquid cultures were grown under continuous illumination in filled screw‐cap tubes. The light source was three incandescent bulbs of 75 W at a distance of 25 cm. Cultures of *C*. *sphaeroides* were incubated at 30°C. *Escherichia coli* was grown in LB (lysogeny broth) medium at 37°C (Ausubel 1987). When required, antibiotics were added at the indicated concentrations: for *C*. *sphaeroides*: spectinomycin (50 μg mL^−1^) kanamycin (25 μg mL^−1^), tetracycline (1 μg mL^−1^), hygromycin (20 μg mL^−1^ for liquid cultures and 150 μg mL^−1^ for plates), rifampicin (0.25 μg mL^−1^ for liquid cultures and 2 μg mL^−1^ for plates), and nalidixic acid (20 μg mL^−1^). For *E*. *coli*, ampicillin (100 μg mL^−1^) spectinomycin (50 μg mL^−1^) kanamycin (50 μg mL^−1^), tetracycline (10 μg mL^−1^), hygromycin (20 μg mL^−1^ for liquid cultures and 200 μg mL^−1^ for plates), and rifampicin (25 μg mL^−1^ for liquid cultures and 50 μg mL^−1^ for plates). For plates, 15 g L^−1^ of Bacto‐agar (Difco) was added.

**Table 1:** Bacterial strains and plasmids used in this study.

Strain or plasmid	Description	Reference or source
Strains		
*C. sphaeroides*		
AM1	WS8N derivative; Δ*fleQ*::Kan *cckA*_L391F_	Del Campo et al. (2007)
EA1	AM1 derivative; Δ*ctrA*::*aadA*	Vega‐Baray et al. (2015)
JV14	AM1 derivative; Δ*cerI*::*aadA*	This work
JV15	AM1 derivative; *ΔcerI*::Rif	This work
JV16	AM1 derivative; Δ*cerR*::*aadA*	This work
JV17	AM1 derivative; Δ*cerN*::*aadA*	This work
JV18	AM1 derivative; Δ*cerN::*Hyg	This work
JV19	AM1 derivative; Δ*cerM*::*aadA*	This work
JV20	AM1 derivative; Δ*cerN*::Hyg Δ*cerM*	This work
JV21	AM1 derivative; Δ*gtaR*::*aadA*	This work
JV22	AM1 derivative; Δ*gtaR*::*uidA*‐Hyg	This work
JV23	AM1 derivative; ΔRS15160::*uidA*‐Hyg	This work
JV24	AM1 derivative; Δ15394::*uidA*‐Hyg	This work
JV25	AM1 derivative; Δ*cerOp*::*aadA*	This work
JV26	AM1 derivative; *ΔcerI*::Rif Δ*cerM*::*aadA*	This work
JV27	AM1 derivative; *ΔcerI*::Rif Δ*cerN*::*aadA*	This work
JV28	AM1 derivative; *ΔcerI*::Rif Δ*ctrA*::*uidA*‐*aadA*	This work
JV29	AM1 derivative; Δ*cerN*::*aadA* Δ*gtaR*::*uidA*‐Hyg	This work
JV30	AM1 derivative; Δ*cerN*::*aadA* ΔRS15160::*uidA*‐Hyg	This work
JV31	AM1 derivative; Δ*cerN*::*aadA* Δ15394::*uidA*‐Hyg	This work
JV32	AM1 derivative; Δ*cerI*::*aadA* *cerM*_Q189amb_	This work
JV33	AM1 derivative, Δ*cerN*::Hyg 14710::*aadA*	This work
*E. coli*		
JV33	MC41000 derivative; carries *cerM*BS1::*lacZ* in the chromosome	This work
TOP10	Cloning strain	Invitrogen
Rosetta	Protein expression strain	Novagen
TE2680	F^‐^ λ^‐^ IN(*rrnD*‐r*rnE*)1 D(*lac*)*X74 rpsL galK2* r*ecD1903*::Tn*10d*‐Tet *trpDC700*::*putPA1303*::[Kan^s^‐Cam^r^‐*lac*]	Elliott (1992)
MC4100	F‐, *[araD139]*_*B/r*_, *Δ(argF‐lac)169*, *λ e14‐*, *flhD5301*, *Δ(fruK‐yeiR)725(fruA25)*, *relA1*, *rpsL150*(Str^r^), *rbsR22*, *Δ(fimB‐fimE)632(::IS1)*, *deoC1*	Casadaban (1976)
*A. tumefaciens*		
NTL4	Reporter strain for detecting long‐chain Reporter strain for detecting long‐chain AHL. Contains the plasmid pZLR4, carrying *traG::lacZ* reporter fusion.	Farrand, Qin, and Oger (2002)
Plasmids		
pTZ18R	Cloning vector; Kan^r^	Amersham
pTZ_*cerI*	pTZ1R carrying *cerI*	This work
pTZ_cerRuPdW	pTZ18R carrying upstream and downstream fragments for *cerR* deletion	This work
pTZ_cerNuPdW	pTZ18R carrying upstream and downstream fragments for *cerN* deletion	This work
pTZ_cerMuPdW	pTZ18R carrying upstream and downstream fragments for *cerM* deletion	This work
pTZ_ΔcerN::Hyg‐ΔcerM	pTZ18R carrying upstream and downstream fragments for ΔcerN::Hyg‐ΔcerM mutation	This work
pTZ_gtaRuPdW	pTZ18R carrying upstream and downstream fragments for *gtaR* deletion	This work
pTZ_RS15160uPdW	pTZ18R carrying upstream and downstream fragments for RS15160 deletion	This work
pTZ_15394uPdW	pTZ18R carrying upstream and downstream fragments for 15394 deletion	This work
pTZ_cerRuP‐cerXdW	pTZ18R carrying upstream and downstream fragments for *cerR*‐ORF2‐*cerI* deletion	This work
pTZ_cerM	pTZ18R carrying *cerM*	This work
pTZ_cerR	pTZ18R carrying *cerR*	This work
pTZ_cerN	pTZ18R carrying *cerN*	This work
pTZ_14710mut	pTZ18R carrying a 1276 bp fragment that includes ORF RSWS8N_14710	This work
pTZ_14710	pTZ18R carrying a 430 bp fragment of RSWS8N_14710	This work
pTZ_cerOp	pTZ18R carrying *cerR‐*ORF2*‐cerI*	This work
pTZ_cerMpET	pTZ18R carrying *cerM* for fusion with a 6xHis tag	This work
pTZ_cerNpGEX	pTZ18R carrying *cerN* for fusion with GST	This work
pJQ200mp18	Mobilizable suicide vector for *C. sphaeroides*; Gm^r^	Quandt and Hynes (1993)
pJQ_Δ*cerI::aadA*	pJQ200mp18 carrying Δ*cerI::aadA*	This work
pJQ_Δ*cerI*::Rif	pJQ200mp18 carrying Δ*cerI*::Rif	This work
pJQ*_*Δ*cerR::aadA*	pJQ200mp18 carrying Δ*cerR*::*aadA*	This work
pJQ*_*Δ*cerN::aadA*	pJQ200mp18 carrying Δ*cerN*::*aadA*	This work
pJQ*_*Δ*cerN::*Hyg	pJQ200mp18 carrying Δ*cerI::*Hyg	This work
pJQ*_*Δ*cerM::aadA*	pJQ200mp18 carrying Δ*cerM*::*aadA*	This work
pJQ*_*Δ*cerN::*Hyg‐Δ*cerM*	pJQ200mp18 carrying Δ*cerN*::Hyg‐Δ*cerM*	This work
pJQ*_*Δ*gtaR::aadA*	pJQ200mp18 carrying Δ*gtaR::aadA*	This work
pJQ*_*Δ*gtaR::uidA‐*Hyg	pJQ200mp18 carrying Δ*gtaR*::*uidA‐*Hyg	This work
pJQ_ΔRS15160*::uidA‐*Hyg	pJQ200mp18 carrying ΔRS15160::*uidA‐*Hyg	This work
pJQ_14710::aadA	pJQmp18 carrying RSWS8N_14710::*aadA*	This work
pJQ_Δ15394*::uidA‐*Hyg	pJQ200mp18 carrying Δ15394::*uidA‐*Hyg	This work
pJQ*_*Δ*cerOp::aadA*	pJQ200mp18 carrying Δ*cerOp*::*aadA*	This work
pRK415	Expression vector used in *C. sphaeroides*; Tc^r^	Keen et al. (1988)
pcerM	pRK415 derivative expressing *cerM*	This work
pcerN	pRK415 derivative expressing *cerN*	This work
pcerR	pRK415 derivative expressing *cerR*	This work
p14710	pRK415 derivative expressing ORF RSWS8N_14710	This work
pcerOp	pRK415 derivative expressing *cerR‐*ORF2‐*cerI*	This work
pcerOpΔcerR	pcerOp derivative Δ*cerR*	This work
pcerOpΔorf‐2	pcerOp derivative in which the region from −19 to +3 of the coding region of ORF‐2 was deleted	This work
pcerI	pRK415 derivative expressing *cerI*	This work
pET28a	Expression vector for His6x‐tagged proteins, Kan^r^	Novagen
pET_6xH‐cerM	pET28a derivative expressing CerM fused to His6x at its N‐terminus	This work
pGEX‐4T‐2	Expression vector for GST‐tagged proteins, Ap^r^	Amersham
pGEX_cerN	pGEX‐4T‐2 derivative expressing GST‐CerN	This work
pGEX_cerM	pGEX‐4T‐2 derivative expressing 6xHis‐CerM	This work
pGEX_cerNM	pGEX‐4T‐2 derivative expressing GST‐CerN and 6xHis‐CerM	This work
pRS551	Ap^r^ Km^r^ *lacZ*; operon fusion vector	Simons, Houman, and N. Kleckner (1987)
pcerMBS1::lacZ	PRS551 derivative carrying the transcriptional fusion *cerM*BS1::*lacZ*	This work
pBBR1gus	Plasmid source of the *uidA* gene	Girard et al. (2000)
pIJ963	The plasmid source of the hygromycin cassette	Lydiate et al. (1989)
pWM5	Vector source of the *uidA*‐*aadA* cassette	Metcalf and Wanner (1993)

### 2.2 Mutant Strains and Plasmids Constructed for This Work

Mutant alleles isolated in this work were cloned into the suicide plasmid pJQ200mp18 (Quandt and Hynes 1993) and introduced into the appropriate strain of *C. sphaeroides* by conjugation (Davis, Donohue, and Kaplan 1988; Figurski and Helinski 1979; Hall, Donohue, and Peters 2023). Mutant strains were selected in which double homologous recombination events occurred, resulting in the replacement of the wild‐type chromosomal gene with the mutant allele. Details of the procedures to isolate the required plasmids for obtaining the *C*. *sphaeroides* mutants are included in Supporting File S1.

To isolate the *E. coli* strain MC41000 *cerM*BS1::*lacZ*, the plasmid pRS551_cerMBS1::lacZ was digested with XhoI, and the resulting linear vector was transformed into *E. coli* TE2680 to recombine the DNA fragment corresponding to the *cerM*BS1::*lacZ* fusion into the *trp* locus on the chromosome (Elliott 1992). This fusion was then mobilized from the TE2680 strain to the MC4100 strain through P1vir transduction (Rosenfeld and Brenchley 1980).

### 2.3 Motility Assays

Motility was tested in soft‐agar plates (0.2% agar) containing Sistrom's minimal medium using 0.2% casamino acids as the carbon source. A volume of 2 μL of an overnight culture in the stationary phase was placed on the surface of the agar. Plates were incubated in an anaerobic jar using BD GasPak EZ gas‐generating sachets under continuous illumination. After incubation, plates were illuminated from below using a lighting box covered with a dark circle to create an oblique illumination (Wolfe and Berg 1989), and photographs were taken using a digital camera.

### 2.4 Protein Overexpression and Purification

Affinity purification of 6xHis‐CerM was performed according to previously published protocols suitable for His‐tagged proteins (https://www.qiagen.com/us/resources/resourcedetail?id=79ca2f7d-42fe-4d62-8676-4cfa948c9435&lang=en). In brief, the *E. coli* Rosetta pET28a_6xHis‐cerM strain was grown overnight in LB medium and subcultured in 100 mL of fresh medium to an initial OD_600_ of 0.05. Once it reached an OD_600_ of 0.6, IPTG was added to a final concentration of 0.1 mM, and the culture was incubated for 4 h at 30°C. The cells were collected and resuspended in 5 mL of phosphate buffer (50 mM NaH_2_PO_4_, 300 mM NaCl, 10 mM imidazole, pH 8). Lysozyme (1 mg mL^−1^) was added, and the cells were incubated for 30 min on ice in the presence of a protease inhibitor (*cOmplete Protease Inhibitor*, Roche). After this time, cell protoplasts were sonicated, and the cell debris was removed by centrifugation. The supernatant was incubated with 200 µL Ni‐NTA agarose beads (QIAGEN) with gentle agitation for 1 h at 4°C. The mixture was loaded onto a column and allowed to flow through. The column was washed three times with wash buffer (50 mM NaH_2_PO_4_, 300 mM NaCl, 20 mM imidazole, pH 8), and the protein was eluted with buffer (50 mM NaH_2_PO_4_, 300 mM NaCl, 250 mM imidazole, pH 8). Following this procedure, approximately 26 mg L^−1^ of protein was obtained. The protein was dialyzed in PBS, pH 7.4.

To obtain GST‐CerN, Rosetta pGEX‐4T‐2_GST‐cerN was grown overnight in LB medium and subcultured in 100 mL of fresh medium to an initial OD_600_ of 0.05. Once it reached an OD_600_ of 0.6, IPTG was added to a final concentration of 0.1 mM, and the culture was incubated for 4 h at 30°C. The cells were pelleted and resuspended in 5 mL of PBS buffer (pH 7.4) supplemented with 1 mg mL^−1^ lysozyme and 20% glycerol and incubated for 1 h on ice in the presence of a protease inhibitor (*cOmplete Protease Inhibitor*, Roche). The cells were sonicated, and the cell debris was removed by centrifuging. The supernatant was incubated with 100 μL of glutathione‐agarose beads (Sigma) with gentle agitation for 1 h at 4°C. The mixture was loaded onto a column and allowed to flow through. The column was washed three times with PBS buffer, and the protein was eluted with buffer (50 mM Tris‐HCl, 10 mM reduced glutathione, pH 8). Following this procedure, approximately 12 mg L^−1^ of protein was obtained. The protein was dialyzed in PBS buffer (pH 7.4).

Protein quantification was performed using the Bio‐Rad protein assay dye reagent with BSA as the standard.

### 2.5 Antibodies and Western Blot Analysis

Polyclonal antibodies were raised in female BALB/c mice against the 6xHis‐CerM protein as described (Harlow and Lane 1988). Total cell extracts were subjected to SDS‐PAGE, and proteins were blotted onto nitrocellulose and tested with the indicated antibody: α‐CerM (obtained in this work, 1:10,000), or with α‐CtrA, α‐FlgE2 (laboratory collection), or α‐His (Qiagen), following previously reported protocols (Harlow and Lane 1988). Anti‐mouse IgG AP conjugate (Sigma‐Aldrich) was used as the secondary antibody. Detection was done using the CDP‐Star substrate (Applied Biosystems).

### 2.6 Pull‐Down Experiment

The strains Rosetta/pET_6xH‐cerM and Rosetta/pGEX_cerN were induced as described. Cells were collected and resuspended in 5 mL of PBS buffer (pH 7.4), supplemented with 1 mg mL^−1^ lysozyme and 20% glycerol, and incubated for 1 h on ice in the presence of a protease inhibitor (*cOmplete Protease Inhibitor*, Roche). The cells were sonicated, and cell debris was removed by centrifugation. The supernatants were incubated together in the absence or presence of 100 µL of the AHL obtained from the cell culture supernatant of AM1 and 100 µL of glutathione‐agarose beads (Sigma). The samples were incubated with gentle agitation for 1 h at 4°C. The mixture was then loaded onto a column and allowed to flow through. The column was washed three times with PBS buffer or PBS‐AHL. The proteins were eluted with elution buffer (50 mM Tris‐HCl, 10 mM reduced glutathione, pH 8). Samples were analyzed by Western blot or SDS‐PAGE, stained with Coomassie brilliant blue R250 (Bio‐Rad).

### 2.7 Concentration of AHL From Cell Culture Supernatants

To concentrate AHL from cell culture supernatants, the previously described ethyl acetate method was used (Eberhard et al. 1981). For this, the autoinducer producer strain AM1 and its derivative *ΔcerI* (JV14) were grown photoheterotrophically in Sistrom's minimal medium without succinic acid and supplemented with 0.2% casamino acids as the carbon source. A volume of 20 mL of cell‐free supernatant was mixed with an equal volume of acidified ethyl acetate (0.1% acetic acid), shaken vigorously for 15 min to ensure thorough mixing, and centrifuged for 5 min at 3500 rpm at 4°C. The upper phase was transferred to a new tube and left in the fume hood to evaporate the solvent. Once dried, 200 µL of 100% ethanol was added. The presence of the autoinducer was verified through the *Agrobacterium tumefaciens* NTL4 pZLR4‐*traG*::*lacZ* reporter system (Farrand, Qin, and Oger 2002).

### 2.8 AHL Detection Assay

To assay the activity promoted by the reporter strain *A. tumefaciens* NTL4 (pZLR4‐*traG*::*lacZ*), an overnight culture was diluted to an OD_600_ of 0. 05 in LB, supplemented or not with the autoinducer. *N*‐(3‐hydroxy‐7‐*cis*‐tetradecenoyl)‐l‐homoserine lactone (Cayman) was used at the following concentrations: 100 nM, 1 μM, and 3 μM. Alternatively, 2 μL of the extracts freshly prepared from cell cultures supernatants was added. The cultures were incubated for 18 h at 30°C. The cells were collected, and β‐galactosidase activity was determined.

### 2.9 RT‐PCR

Total RNA was isolated from cells grown to an optical density at 600 nm (OD_600_) of 0.6 in Sistrom's minimal medium using the RiboPure kit (Ambion). Reverse transcription and PCR amplification were performed with the Access RT‐PCR system (Promega) according to the manufacturer's instructions. A control reaction mixture lacking reverse transcriptase was run in parallel. The PCR products were analyzed using acrylamide gel electrophoresis. The oligonucleotides DOWNcerRfw and asRNAcerI were used to test the region between *cerR* and *cerI*, amplifying a product of 379 bp. FwpctrAXbaI and RvctrAEcoRI were used to amplify a region of 285 bp corresponding to the upstream region of *ctrA* as a control.

### 2.10 *In Vivo* Determination of CerM DNA‐Binding Ability

The reporter strain MC4100 carrying the *lacZ* gene under the control of the artificial promoter *cerM*BS1::*lacZp* at the *trp* locus was transformed with the pGEX‐4T‐2 derivative plasmids that expressed 6XHis‐CerM, GST‐CerN, or both proteins simultaneously. As a negative control, the reporter strain was also transformed with empty pGEX‐4T‐2. These strains were grown in 2 mL of LB medium with or without AHL obtained from the cell culture supernatant of AM1 for 3.5 h at 37°C. Cells were collected by centrifugation, and β‐galactosidase activity was determined.

### 2.11 β‐Glucuronidase Assays

Total cell extracts were used to assay β‐glucuronidase activity. These extracts were prepared using cultures in the exponential phase of growth. Cells were concentrated sixfold before lysis. Enzymatic activities were performed following previously reported protocols (Jefferson, Burgess, and Hirsh 1986). Protein content was determined using the Bio‐Rad protein assay dye reagent with BSA as the standard.

### 2.12 β‐Galactosidase Assays

The assays were carried out according to the OpenWetWare protocol: β‐Galactosidase Assay (A better Miller) (http://openwetware.org/mediawiki/index.php?title=Beta-Galactosidase_Assay_(A_better_Miller)&oldid = 620416).

### 2.13 Electrophoretic Mobility Shift Assays (EMSA)

The assays were carried out based on the previously reported protocol (Hellman and Fried 2007) with some modifications. In brief, 50 ng of DNA was incubated in the absence or presence of 50, 100, or 200 ng of 6xHis‐CerM in a Binding Buffer (10 mM Tris [pH 7.5], 1 mM EDTA, 0.1 M KCl, 0.1 mM DTT, 5% v/v glycerol, 0.10 mg mL^−1^ BSA) for 20 min at room temperature. When required, GST‐CerN was added at an equimolar concentration relative to 6xHis‐CerM. Concentrated supernatants or synthetic autoinducers were added if necessary. When 6xHis‐CerM and GST‐CerN were incubated together, they were preincubated for 30 min at room temperature before adding the DNA. The DNA fragments tested in these assays were obtained by PCR using the oligonucleotides listed in Table S1. Mixtures were separated on a 4% Acrylamide/Bisacrylamide (30:0.8) nondenaturing gel using 1X electrophoresis buffer (TAE buffer: 40 mM Tris, 2.5 mM EDTA, brought to pH 7.8 with acetic acid). Gels were stained with ethidium bromide and photographed under UV light.

### 2.14 RNA Seq Experiments

RNA‐seq was conducted as previously described (Hernández‐Valle et al. 2020). *C*. *sphaeroides* AM1/pRK415 and Δ*cerN* ^spc^/pcerM were grown in Sistrom's minimal medium with 0.2% casamino acids under photoheterotrophic conditions. When cultures reached an OD_600_ of 0.6, cells were harvested, and total RNA was isolated using the RiboPure RNA Purification kit (Thermo Fisher) according to the manufacturer's instructions. RNA quality was assessed by capillary electrophoresis using the 2100 Bioanalyzer (Agilent Technologies). rRNA depletion was carried out using the Ribo‐Zero Plus rRNA Depletion Kit according to the manufacturer's instructions. Library preparation was done following the high sample protocol from the TruSeq Stranded mRNA Sample Preparation Guide (Illumina). cDNA libraries were subjected to 2 × 150 bp paired‐end sequencing on the Illumina HiSeq platform. The number of reads that mapped to a unique location on the WS8N genome were 163,336,124 and 208,340,108 for AM1/pRK415 and Δ*cerN* ^spc^/pcerM, respectively. Differential gene expression analysis was carried out with DESeq. 2 (version 1.10.1). Genes with a log_2_ fold change of ≥ 2 or ≤ −2 and an adjusted *p‐*value (*p*adj) of ≤ 0.01 were considered differentially expressed (DE).

### 2.15 In Silico Analysis

To identify novel components of the QS of *C. sphaeroides*, the HMM profiles PF03472.18, PF00196.22, and PF00765.20, corresponding to the Autoind_bind, GerE (HTH), and Autoind_synth domains, respectively, were obtained from the Pfam database using the program hmmfetch from HMMER on terminal (HMMER 3.4, http://hmmer.org/) Subsequently, these profiles were compared against the annotated proteins of *C. sphaeroides* WS8N. The cutoff values were set at scores of 35, 20, and 50, for the Autoind_bind, GerE (HTH), and Autoind_synth domains, respectively. The presence of the Autoind_bind domain was required to define a LuxR regulator, while the presence of the Autoind_synth domain was required to define a LuxI autoinducer synthase. The program OrthoFinder 2.5.5 (Emms and Kelly 2019) was run with default parameters, using Diamond to compare the provided sequences. The input consisted of the complete annotated proteins of *C. sphaeroides* WS8N (GCF_000212605.1), *R. capsulatus* SB 1003 (GCF_000021865.1), *D. shibae* DF12 (GCF_000018145.1), *Ruegeria* sp. KLH11 (*Rhodobacteraceae bacterium* KLH11: GCF_000158135.1), and *Sinorhizobium meliloti* 1021 (GCF_000006965.1), accounting for 22,618 peptide sequences. These sequences were organized into 4375 orthogroups. Orthofinder's output also included the species tree and gene trees for all orthogroups.

To evaluate synteny, the online version of the program SyntTax (https://archaea.i2bc.paris-saclay.fr/SyntTax/) (Oberto 2013) was run with a minimum value score of 10%. The input consisted of the peptide sequences of the QS proteins, which were compared with the genomes of *R. capsulatus* SB 1003, *D. shibae* DF12, and *Ruegeria pomeroyi* DSS3.

### 2.16 In Silico Search for the CerM Binding Site

To identify a motif that could correspond to the CerM binding site, the 381 genes DE between Δ*cerN*/pcerM and AM1/pRK415 (log_2_FC ≥ 2 or ≤ −2) were filtered to eliminate 200 genes known to be controlled by CtrA. Considering a log_2_FC ≥ 1 or ≤ −1, the CtrA regulon consists of 546 genes under the growth conditions reported previously (Hernández‐Valle et al. 2020). This resulted in 181 genes, organized into 105 transcriptional units (operons). An operon was defined as genes of the same orientation that are closer than 50 bp. Analysis of these 105 regions using MEME 5.5.5 with the default parameters (Bailey et al. 2015) did not reveal a conserved motif. To make this analysis more specific, we searched this data set with a position weight matrix (PWM) that describes the GtaR binding site. This was on the rationale that GtaR and CerM are likely paralogous and would recognize somewhat similar sites. Consequently, this strategy would identify the GtaR binding sites that may be part of our data set, as well as those sites recognized by CerM.

This search was done using the program “RSAT matrix scan quick and simple,” with a probability value cutoff of 1*e*‐4 (Turatsinze et al. 2008) and the GtaR PWM. Given the palindromic nature of the GtaR binding site, frequently two hits were computed for each promoter; we also noted that in a single regulatory region, several hits were occasionally found.

To build the PWM modeling of the GtaR binding site, we selected a 300 bp region upstream and 10 bp downstream of the start methionine of GtaR orthologs of 24 species of Rhodobacteraceae (Table S2), including *C. sphaeroides*. Orthologs were identified using the best reciprocal hit method with BLASTp, with a minimum coverage of 70% and a minimum sequence identity of 35%. These sequences were analyzed with MEME and revealed a 22 bp motif, RACMTGTCYWWWWRGACAKGTY, with an *e*‐value of 1.1*e*−048, present in 18 of the 24 sequences.

## 3 Results

### 3.1 *In Silico* Identification of Novel Components of the QS System of *C. sphaeroides* and Their Phylogenetic Relationships

To gain better insight into the QS system of *C. sphaeroides*, we conducted a comprehensive identification of the QS components in this bacterium. For this, we performed a domain search with HMMER, seeking the domains present in LuxI (Autoind_synth [PF00765.20]) and LuxR (Autoind_bind [PF03472.18] and GerE/HTH [PF00196.22]). In both cases, the searches were conducted against the annotated proteins of *C. sphaeroides* WS8N. This analysis led to the identification of five LuxR homologs that possess the distinctive domains of the LuxR regulators (the Autoind_bind and GerE/HTH domains); this group includes RSWS8N_05820 (hereafter referred to as CerM), RSWS8N_13935, RSWS8N_15394, RSWS8N_RS15160 and RSWS8N_05825 formerly described as CerR (Puskas et al. 1997). Interestingly, we also found a LuxR homolog, RSWS8N_05815 (hereafter referred to as CerN) that has the autoinducer binding domain but lacks the HTH domain. For LuxI, we identified a single homolog, RSWS8N_05830, which corresponds to the previously reported CerI (Puskas et al. 1997) (Table 2). The LuxR regulators have six highly conserved residues (W57, Y61, D70, P71, W85, and G113) that according to the reported crystal structure of TraR, are part of the AHL‐binding cavity (Covaceuszach et al. 2013; Fuqua, Winans, and Greenberg 1996; González and Venturi 2013; Lang and Faure 2014; Vannini 2002; Zhang et al. 2002). We observed that, except for CerM that shows a conservative substitution (W57F), and RSWS8N_RS15160 that shows the change Y61S, the remaining positions in the AHL‐binding sites of the LuxR proteins of *C. sphaeroides* are conserved (Table 2).

**Table 2:** LuxR and LuxI homologs in *C. sphaeroides* genome identified by HMMER.

	ORF	Gene*	ID	HMMER						
				Autoind_bind		GerE_HTH		Autoind_synth		AA in AHL pocket**
				Score	*e*‐value	Score	*e*‐value	Score	*e*‐value	
**LuxR**	RSWS8N_05825	*cerR*	WP_002720271.1	128.1	4.30E−38	63.4	2.60E−18			WYDPWG
	RSWS8N_RS16160	RS15160	WP_082242126.1	77.1	2.10E−22	68.2	8.10E−20			W **S** DPWG
	RSWS8N_13935	*gtaR*	WP_011336955.1	51.6	1.60E−14	24.6	3.20E−06			WYDPWG
	RSWS8N_05820	*cerM*	WP_011338009.1	47.5	2.90E−13	42.9	6.50E−12			**F** YDPWG
	RSWS8N_15394	15394	WP_002723109.1	38.7	1.50E−10	37.7	2.70E−10			WYDPWG
	RSWS8N_05815	*cerN*	WP_009563908.1	66.1	5.30E−19					WYDPWG
**LuxI**	RSWS8N_05830	*cerI*	WP_002720272.1					281.5	5.20E−85	

* The genes *cerR* and *cerI* were reported previously (Puskas et al. 1997). The names *gtaR*, *cerM*, and *cerN* were assigned in this work.

** Conserved residues in the AHL pocket include W57, Y61, D70, P71, W85, and G113. Substitutions are in bold and underlined.

**Figure 1:**
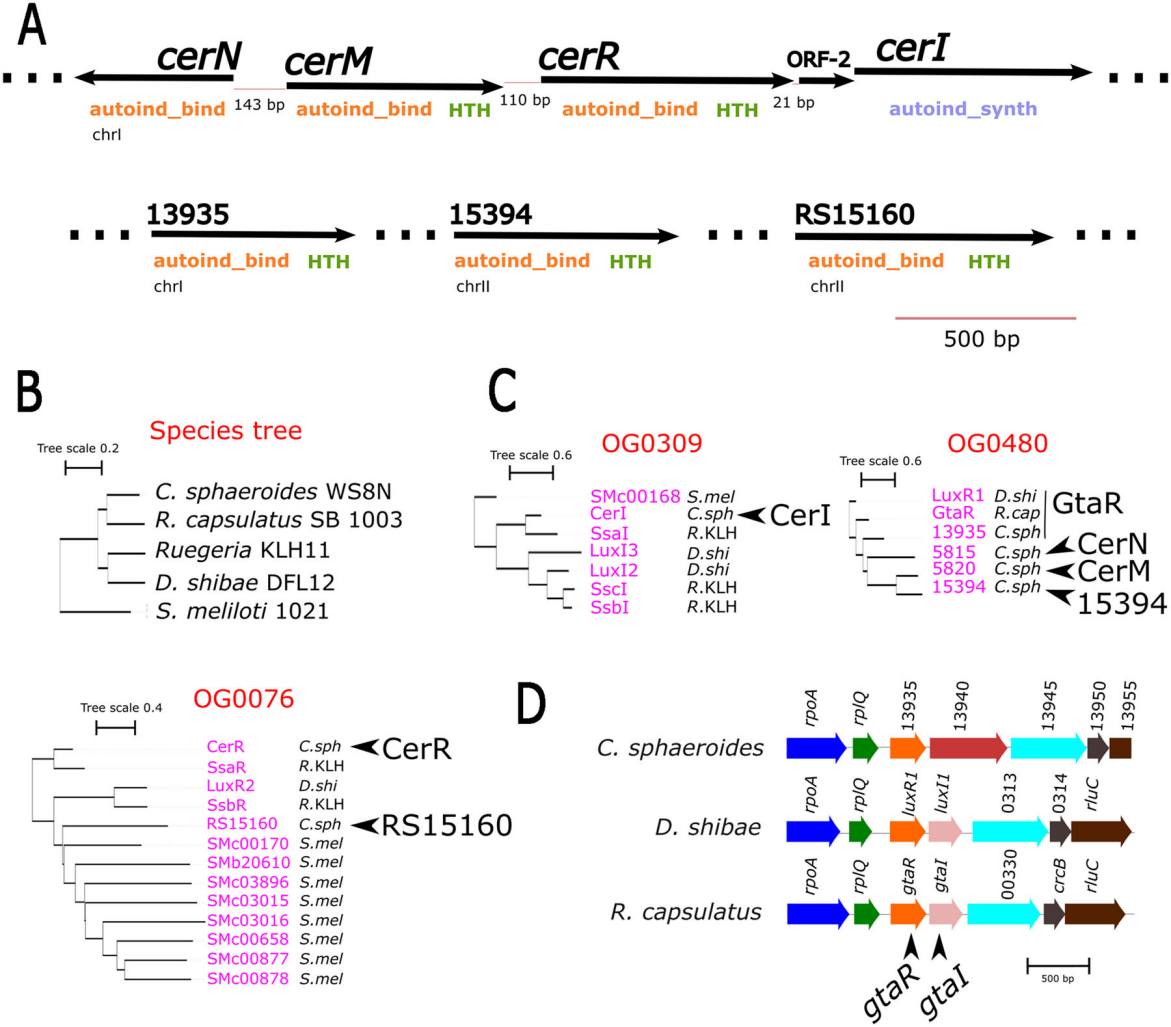
Quorum sensing components of *C*. *sphaeroides*. (A) Genes encoding LuxR and LuxI homologs present in the complete genome of *C. sphaeroides*. *cerI* and *cerR* correspond to the previously characterized homologs of *luxI* and *luxR*, respectively. Three continuous dots represent a brake to another position in chromosome I or II. The distance between continuous genes is indicated. Below each arrow, the domains identified in the proteins encoded by these genes are shown. (B) Species tree showing the phylogenetic relationships among *C. sphaeroides* WS8N, *Rhodobacter capsulatus* SB 1003, *Dinoroseobacter shibae* DF12, *Ruegeria* sp. KLH11, and *Sinorhizobium meliloti* 1021, created with OrthoFinder. (C) Phylogenetic gene trees for orthogroups OG0309, OG0480, and OG0076, which contain the quorum‐sensing components of *C. sphaeroides*, constructed with OrthoFinder using the complete annotated proteins of the above‐mentioned organisms. Gene identifiers are colored in magenta, while species names are in black, each with a specific code: *C. sphaeroides* WS8N (*C*. *sph*), *R. capsulatus* SB 1003 (*R*. *cap*), *D. shibae* DF12 (*D*. *shi*), *Ruegeria* sp. KLH11 (*R*. KLH), and *S. meliloti* 1021 (*S*. *mel*). (D) Synteny conservation analysis of the quorum sensing genes of *C. sphaeroides* in *D. shibae, R. capsulatus*, and *Ruegeria* sp. KLH11, using the program SyntTax. Only the gene RSWS8N_13935 showed synteny conservation with *D. shibae* and *R. capsulatus*. Homologous genes are depicted with arrows of the same color, and gene identifiers are colored in black.

Examining the genomic context of these genes showed that two of the five *luxR* homologs identified in this search are found upstream of *cerR*, and the others are distributed along chromosomes I and II (Figure 1A).

To determine the phylogenetic relationships between the QS components of *C. sphaeroides* and the QS proteins of other bacteria in which a connection between QS and the TCS CckA/ChpT/CtrA has been described, we conducted a genome‐wide orthologous group inference using the program Orthofinder. In this analysis, we used the annotated proteins of *D. shibae* DFL 12, *R. capsulatus* SB 1003, and *Ruegeria* sp. KLH11. The proteins from *Sinorhizobium meliloti* were also included to increase the QS components from another well‐characterized α‐proteobacteria that may reveal other possible relationships, even though no relationship between the TCS CckA/ChpT/CtrA and the QS system has been reported in this specie.

**Figure 2:**
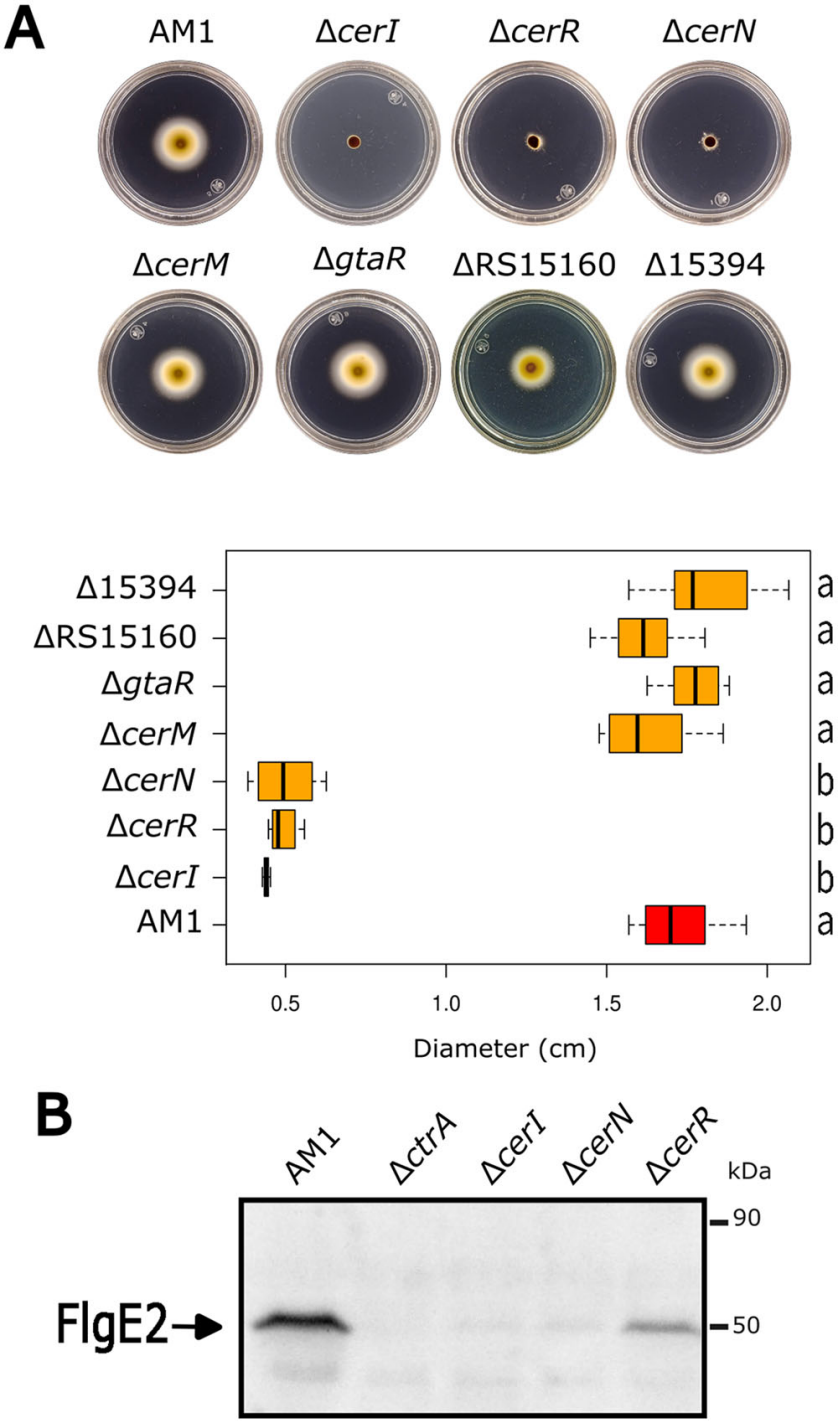
Swimming proficiency and detection of the flagellar hook protein (FlgE2) in mutant strains of the QS system. (A) swimming proficiency of AM1 derivatives strains carrying the indicated mutation. Above, soft‐agar plates containing Sistrom's minimal medium supplemented with 0.2% casamino acids were inoculated and incubated for 60 h under photoheterotrophic conditions. JV14 (Δ*cerI*::*aadA*), JV16 (Δ*cerR*::*aadA*), JV17 (Δ*cerN*::*aadA*), JV19 (Δ*cerM*::*aadA*), JV21 (Δ*gtaR*::*aadA*), JV23 (ΔRS15160::*uidA*‐*aadA*), JV24 (Δ15394::*uidA*‐*aadA*). Below is a boxplot representing the distribution of swimming ring diameters from triplicates of at least three independent experiments. The data for the parental strain AM1 are shown in a red box. (B) Western blot analysis of total cell extracts of the nonmotile strains EA1 (Δ*ctrA*::*aadA*), JV14 (Δ*cerI*::*aadA*), JV17 (Δ*cerN*::*aadA*), and JV16 (Δ*cerR::aadA*) were tested using anti‐FlgE2 antibody. Statistical significance was determined using one‐way ANOVA followed by Tukey's post‐hoc analysis. Groups sharing the same letter (a, b) are not significantly different, while different letters indicate significant differences (*p* < 0.01).

A total of 4983 orthogroups resulted from this analysis, and a species phylogenetic tree was inferred by the same program (Figure 2B). The *C*. *sphaeroides* QS proteins were found in three orthogroups. The autoinducer synthase CerI was grouped into orthogroup OG0309 with the three synthases from *Ruegeria* sp. KLH11 (SsaI, SsbI, and SscI), two from *D. shibae* DFL 12 (LuxI2 and LuxI3), and one from *S. meliloti* (Figure 2C); notably, CerI was on the same branch of the tree as SsaI from *Ruegeria* sp., which has been implicated along with SsaR, in controlling the TCS CckA/ChpT/CtrA. As expected from the close relationship between SsaI and CerI, we found that CerR was clustered with SsaR in orthogroup OG0076, which also includes RSWS8N_RS15160 in a different clade. The remaining 4 LuxR regulators of *C. sphaeroides* were in orthogroup OG0480, together with GtaR from *R. capsulatus* (GtaR_*Rc*_) and LuxR1 from *D. shibae*. The clustering of RSWS8N_05820, RSWS8N_05815, and RSWS8N_15394 in a single clade suggests that these proteins could be paralogous to RSWS8N_13935 and that RSWS8N_13935 is the ortholog of GtaR_Rc_ and LuxR1 (Figure 2C).

Additionally, we conducted a synteny analysis for the QS components of *C. sphaeroides*. We found that RSWS8N_13935 is the only one that exhibits conserved synteny with *luxR1* of *D. shibae* and *gtaR*_*Rc*_, supporting the proposal that these three regulators are orthologues. Therefore, RSWS8N_13935 will be named from here on as *gtaR*. Importantly, this analysis also revealed the loss of the orthologue of *gtaI*/*luxI1* in *C. sphaeroides* (Figure 2D).

### 3.2 Components of the QS System Are Involved in the Control of the TCS CckA/ChpT/CtrA

In *C. sphaeroides*, motility mediated by the Fla2 flagella is dependent on the TCS CckA/ChpT/CtrA. Therefore, to investigate if there is a connection between these systems, we evaluated the swimming ability of AM1 cells in the absence of different QS genes. For this, we isolated single null mutants by interrupting *cerI* and each of the six *luxR* homologs with a resistance cassette (*aadA*‐Spc^R^), and their swimming ability were tested in soft‐agar plates. We observed that strains Δ*cerI*, Δ*cerN*, and Δ*cerR* were unable to swim (Figure 2A). A Western blot using total cell extracts of these three mutant strains revealed the absence of the structural flagellar protein FlgE2 in Δ*cerI* and Δ*cerN* strains, and a significant reduction in Δ*cerR*, suggesting that the expression of the *fla2* is controlled by the QS system (Figure 2B).

**Figure 3:**
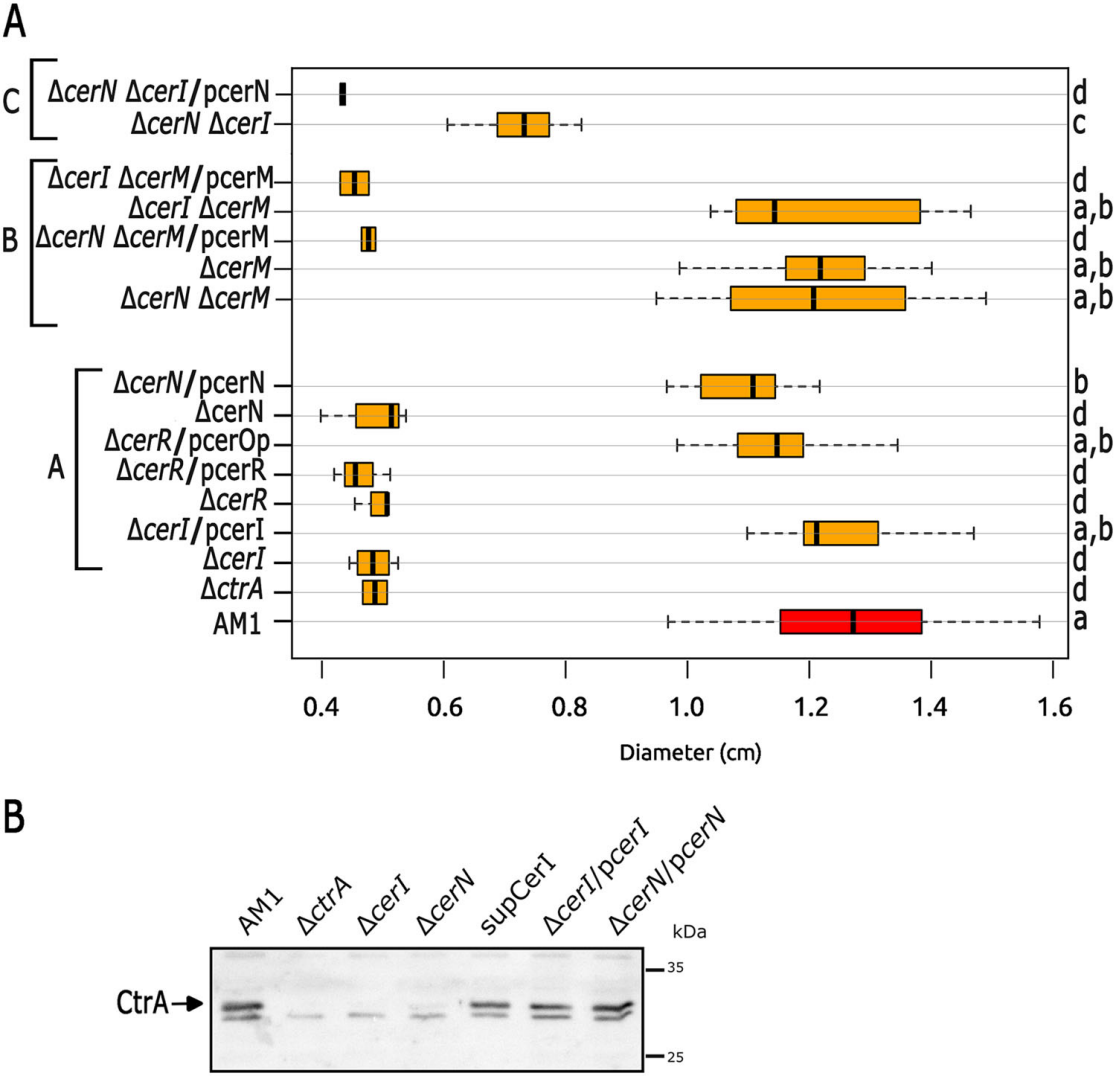
(A) Soft‐agar plates containing Sistrom's minimal medium supplemented with 0.2% casamino acids were inoculated with the indicated strains and incubated for 48 h under photoheterotrophic conditions. EA1 (Δ*ctrA*::*aadA*), JV14 (Δ*cerI*::*aadA*), JV16 (Δ*cerR*::*aadA*), JV17 (Δ*cerN*::*aadA*), JV19 (Δ*cerM*::*aadA*), JV20 (Δ*cerN::*Hyg Δ*cerM*), and JV26 (Δ*cerI::*Rif Δ*cerM::aadA*). Complementation tests were carried out by the introduction of pRK415 plasmid carrying the suitable wild‐type gene; the presence of the plasmid in a particular strain is indicated by a slash followed by the name of the plasmid. A boxplot representing the distribution of swimming ring diameters from triplicates of at least three independent experiments is shown. The data for the parental strain AM1 are shown in a red box. Statistical significance was determined using one‐way ANOVA followed by Tukey's post‐hoc analysis. Groups sharing the same letter (a, b, c, d) are not significantly different, while different letters indicate significant differences (*p* < 0.01). (B) Immunodetection of CtrA in the QS mutant strains. Total cell extracts of the indicated mutants were tested by immunoblotting using an anti‐CtrA antibody (EA1 [Δ*ctrA*::*aadA*], JV14 [Δ*cerI*::*aadA*], JV17 [Δ*cerN*::*aadA*], JV32 [Δ*cerI*::*aadA cerM*_Q189amb_]). Several nonspecific bands were detected on the blot, and identification of CtrA was carried out by including a cell extract of the Δ*ctrA* strain in the experiment. The migration of the molecular weight markers is shown at the right. The signal assigned as CtrA (26.7 kDa) is indicated by an arrow.

The introduction of a plasmid expressing *cerI* or *cerN* restored the swimming proficiency of Δ*cerI* or Δ*cerN* strains, respectively, supporting the role of CerI and CerN in the Fla2‐ phenotype (Figure 3A, Group A). Unexpectedly, no complementation was observed when pcerR plasmid was introduced to strain Δ*cerR*, indicating that this phenotype is caused by another reason beyond the loss of CerR. In agreement with this possibility, we observed that Δ*cerR* strain was complemented using pcerOp plasmid (this plasmid only carries *cerR* and *cerI*) (Figure 3A, Group A), suggesting that the insertion of the resistance cassette in *cerR* affected the expression of *cerI*. Therefore, the role of CerR was evaluated using a different approach described at the end of this section.

Regardless of the role of CerR, our results indicate that CerI and CerN, are required for the expression of *fla2*. It should be noted that CerN does not have a DNA‐binding domain (Table 2), suggesting it may function through a noncanonical mechanism. This led us to investigate the swimming phenotype of double mutant strains in which *cerN* was inactivated along with the other LuxR regulators (Figure S1). The only mutant combination that restored the swimming ability of strain Δ*cerN* was the Δ*cerN* Δ*cerM*. In addition, complementation of this double mutant with pcerM reduced the swimming ring to a similar level of Δ*cerN*, indicating that no other undetected mutation is involved in the observed phenotype (Figure 3A, Group B and also shown in Figure S1). These experiments suggest that CerM is involved, either directly or indirectly, in the negative regulation of the TCS CckA/ChpT/CtrA, with CerN functioning as an antagonist. Importantly, we determined that the double mutant Δ*cerI* Δ*cerM* was able to swim, unlike Δ*cerI* (Figure 3A, Groups B and A, respectively); furthermore, by introducing pcerM into Δ*cerI* Δ*cerM* strain the nonmotile phenotype of the Δ*cerI* mutant was restored (Figure 3A, Group B). These results suggest that the presence of AHL is necessary to override the repressor effect of CerM on swimming. Another line of evidence that supports the proposed regulation model came from the characterization of a suppressor mutant isolated from the flares that emerged around the swimming halo of the Δ*cerI* strain after a prolonged time of incubation. Characterization of this suppressor mutant revealed that it carries a nonsense mutation in *cerM* that changes residue Q189 to a stop codon (TAG, amber), producing a truncated protein lacking the alpha‐helix‐10 of the HTH DNA‐binding domain (Figure S2). This suppressor mutant recapitulates the swimming phenotype of the double mutant Δ*cerI* Δ*cerM*. These results corroborate the notion that CerM exerts a negative effect on swimming, which is nullified by the action of CerN and the autoinducer. Surprisingly, the double mutant Δ*cerI* Δ*cerN* was able to develop a reduced swimming halo (Figure 3A, Group C), raising the possibility that the AHL and CerN may contribute to some degree in stabilizing CerM or its active form.

The swimming behavior of these mutant strains was presumably determined by the expression of CtrA; therefore, we evaluated the steady‐state level of CtrA in strains Δ*cerI*, Δ*cerN*, and the suppressor mutant obtained from Δ*cerI* (SupCerI) (Figure 3B). As expected, CtrA was severely reduced in strains Δ*cerI* and Δ*cerN*. In agreement with the observed phenotype, the suppressor mutant recovered a high level of CtrA, similar to that observed in AM1. Recovery of the CtrA levels was also observed in the complemented strains Δ*cerI*/pcerI and Δ*cerN*/pcerN.

**Figure 4:**
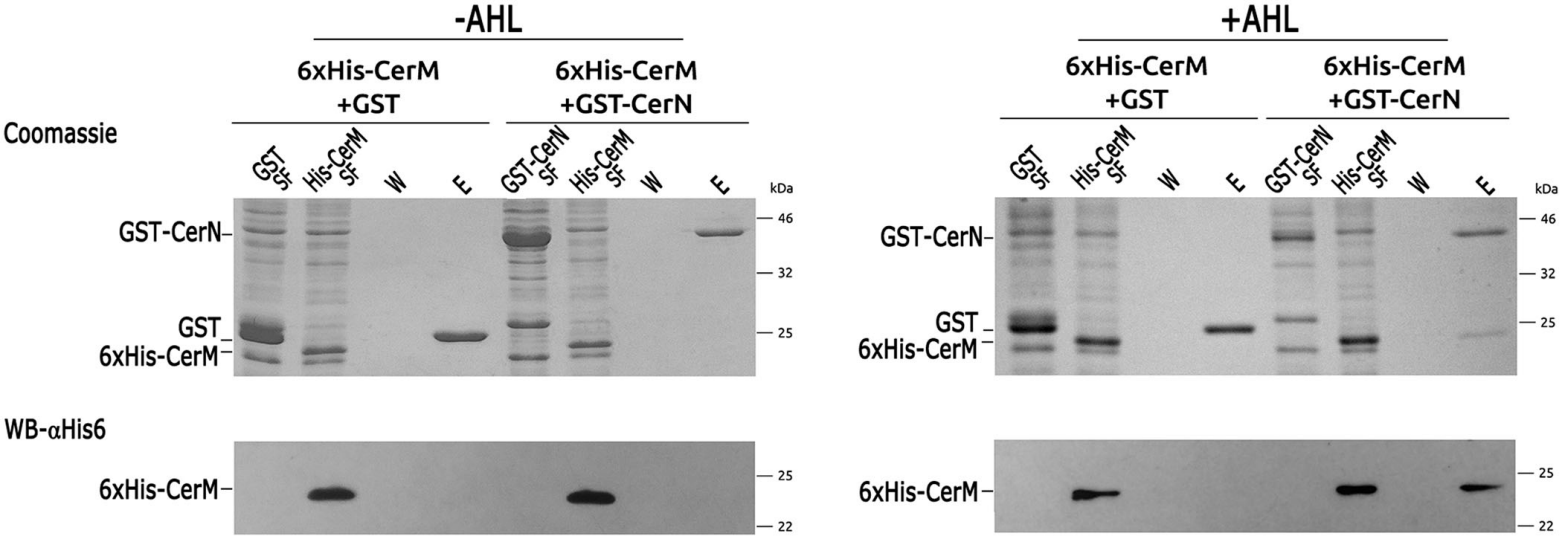
CerM and CerN interaction tested by pull‐down. Total soluble extracts containing GST alone (GST, SF) or GST‐CerN (GST‐CerN, SF), were incubated with total soluble extracts containing 6xHis‐CerM (His‐CerM, SF). After incubation, proteins were captured using glutathione‐sepharose beads, and two different fractions were collected: W (wash) and E (elution). Proteins in each fraction were resolved by SDS‐PAGE and detected by Coomassie brilliant blue R 250 or Western blot using a 6xHis‐tag antibody (image below). The polypeptides GST (29 kDa), GST‐CerN (40 kDa), and 6xHis‐CerM (26 kDa) are indicated.

As mentioned above, the Δ*cerR::aadA* (JV16) strain was complemented by pcerOp (which expresses *cerR* and *cerI*) and not by pcerR, suggesting that the resistance cassette *aadA* could exert a polar effect on the expression of *cerI*. To clarify the role of CerR in the swimming ability of *C. sphaeroides*, and to test the hypothesis concerning the polar effect of the *aadA* cassette, we isolated a *cerR* deletion mutant, without using a resistance cassette. For this, the complete region carrying the genes involved in QS was deleted from the chromosome of AM1. Then, this strain was transformed either with a plasmid carrying the whole intact region or a mutant version in which the *cerR* gene was deleted. It is important to note that a low copy number plasmid was used for all complementation experiments to preserve, as much as possible, the stoichiometry of the QS components. As shown in Figure S3, both strains were able to swim similarly to AM1, indicating that CerR is not related to the control of the TCS CckA/ChpT/CtrA or required for the synthesis of AHL, otherwise, swimming would have been compromised. In agreement, a Western blot assay revealed the presence of CtrA in the absence of *cerR* (FigureS3B). Additionally, by RT‐PCR experiments, we determined that *cerR* and *cerI* are expressed as a single mRNA (Figure S3C), explaining the polar effect of the allele Δ*cerR::aadA* (JV16 strain in Figures 3 and 4). Complementation assays of strain Δ*cerOp* were also useful to determine that the small *orf2*, reported to be between *cerR* and *cerI* (Puskas et al. 1997), does not have a role in the swimming ability of AM1 (Figure S3A).

### 3.3 Autoinducer Production and Detection of CerM in QS Mutant Strains

To evaluate if the absence of CerN could negatively affect the production of the autoinducer, we carried out a functional complementation assay. This experiment determined if Δ*cerI* cells recovered their swimming proficiency when strains Δ*cerN*, Δ*cerM* and AM1 were grown in close proximity. From this assay, it was observed that AHL produced by strains Δ*cerN*, Δ*cerM* and AM1 promoted the swimming of Δ*cerI* (Figure S4). Therefore, neither CerN nor CerM is required for AHL synthesis.

To further characterize these strains, we determined the presence of CerM in total cell extracts of Δ*cerI* and Δ*cerN* cells by Western blot. In Δ*cerI* a slight reduction of CerM was detected when compared with AM1, while a notable reduction of CerM was observed in Δ*cerN* (Figure S5). This result raises the possibility that CerM and CerN could interact, and in this manner, favor CerM stability. The reduced levels of CerM in the Δ*cerI* or Δ*cerN* mutants are still able to completely repress their swimming ability (Figure 3A); however, when combined, we observed a slight recovery in motility, suggesting a shortage of CerM.

### 3.4 CerN Interacts With CerM When the Autoinducer Is Present

The phenotypic analysis of the QS mutants suggests that CerM and CerN may interact with each other, either directly or indirectly. To investigate this possibility, we conducted a pull‐down experiment using 6xHis‐CerM and GST‐CerN. Soluble cell extracts from *E*. *coli* overexpressing these proteins were incubated together, either in the presence or absence of the AHL obtained by extracting the cell culture supernatants of AM1 with ethyl acetate. As a control, the presence of AHL in these samples was determined using *A*. *tumefaciens* NTL4 (pZLR4) as reporter strain (Figure S6); importantly, no signal was detected when the Δ*cerI* supernatant extract was used (Figure S6).

Glutathione affinity‐purified proteins (GST or GST‐CerN) were analyzed by SDS‐PAGE and visualized by Coomassie staining and Western blot. These experiments revealed that in the absence of the autoinducer, only GST‐CerN was recovered (Figure 4, Lane E). In contrast, 6xHis‐CerM was eluted along with GST‐CerN when the AHL was included in the assay (Figure 4, Lane E). Spurious interactions between GST and 6xHis‐CerM were not observed (Figure 4). These results show that CerM and CerN interact in an AHL‐dependent fashion.

### 3.5 CerM Protein Does Not Bind to the Control Region of *cckA chpT* or *ctrA*

If there is a direct regulation of the QS system on the expression of *ctrA*, it would be expected that CerM would be able to bind the control region of *ctrA*, or probably to the control regions of *cckA* or *chpT*. To evaluate this possibility, 6xHis‐CerM protein was purified, and its interaction with the regulatory region of these genes was tested by electrophoretic mobility shift assays (EMSA). None of these regions modified their electrophoretic mobility in the presence of 6xHis‐CerM, suggesting that CerM must indirectly repress the expression of *ctrA* (Figure S7).

### 3.6 Transcriptomic Profiling of Δ*cerN* Strain

Our results suggest that in the absence of AHL, CerM acts indirectly as a negative regulator of *ctrA*; however, in the presence of AHL, CerN antagonizes this action. From this proposal, we hypothesized that among the genes DE in the presence or absence of CerN, it would be possible to identify the transcription factor responsible for directly controlling *ctrA*. With this idea, we compared the global expression profile of strains Δ*cerN*/pcerM and AM1/pRK451, considering that in regard to the expression of CtrA, they represent the repressed and activated states of the QS system. A total of 381 genes were DE between these strains (Figure S8 panels A and B; and Table S3). As expected, this set includes the genes previously reported to be controlled by CtrA, such as *fla2* (flagellar), *cheOp2* (chemotaxis), *gvp* (gas vesicles), *gta* (gene transfer agent), *puc* (photosynthesis), etc. (Hernández‐Valle et al. 2020). In agreement with our observations, a strong downregulation of *ctrA* in Δ*cerN*/pcerM was observed (Table S3). After excluding the genes known to be controlled by CtrA, a total of 181 genes remained, presumably many of these could be directly controlled by CerM/CerN. Therefore, the subsequent analyses were done using this subset.

In general, among the genes regulated by the QS system, we found a large genetic cluster annotated as a Mu‐like prophage (RSWS8N_18039‐RSWS8N_18284) (RS‐Mu) that is downregulated in Δ*cerN*/pcerM. These genes could be directly controlled by the transcription factor encoded by RSWS8N_18024 (putative prophage anti‐repressor), which is also downregulated in this strain and is located upstream of the Rs‐Mu locus (Figure S9). In addition to these genes, a putative operon formed by 7 genes potentially involved in polysaccharide synthesis (RSWS8N_05960‐RSWS8N_05990) is upregulated in this strain. It has been reported that exopolysaccharides are relevant for certain functions such as the sequestration of calcium ions, reactive oxygen species scavenging, prevention of cell agglutination, etc. (Acosta‐Jurado et al. 2021; Aslam et al. 2008; Hawkins, Geddes, and Oresnik 2017; Lehman and Long 2013). In this context, the downregulation of several genes related to oxidative stress protection, such as *sodC* (RSWS8N_14220), RSWS8N_13395 (encoding a putative glutathionyl‐hydroquinone reductase), and *katE* (RSWS8N_02125) appears relevant.

The influence of CerM/CerN on the expression of the *luxR* homologs RSWS8N_RS15160 (log_2_FC = 2.8) and RSWS8N_15394 (log_2_FC = 5.4) was further tested using chromosomally placed transcriptional reporter gene fusions. The expression of *gtaR* (RSWS8N_13935) was also tested using this approach, even though its DE was slightly below the cutoff value (Table S5). These experiments showed that the activity of the reporter gene fusions of RSWS8N_15394 and *gtaR* exhibited an expression pattern consistent with the global expression analysis. Conversely, RSWS8N_RS15160 displayed an inverse pattern to that expected from the RNAseq experiments (Figure S8). For this gene, additional evidence is required to determine whether it should be considered a false positive result or if its expression may be subject to another type of regulation that may be obscured by the reporter gene.

Among the DE genes, we identified 8 genes encoding transcription factors that are upregulated in Δ*cerN*/pcerM, 3 of which do not affect the expression of *fla2*. Two correspond to the LuxR homologs RSWS8N_15394, and RSWS8N_RS15160 (Figure 2), and the third one is the response regulator *dorR* involved in the expression of a DMSO reductase (Mouncey, Choudhary, and Kaplan 1997; Mouncey and Kaplan 1998) (Figure S10). The only downregulated transcriptional factors in the Δ*cerN*/pcerM strain were *ctrA* and a gene that encodes a putative prophage anti‐repressor (RSWS8N_18024). Therefore, we presumed that the product of one of the remaining 5 upregulated transcription factors could be involved in directly controlling the expression of *ctrA* (Table S4).

### 3.7 Identification of the CerM Binding Site

To identify those genes that could be directly controlled by CerM, we aimed to identify the possible binding site of this protein. The binding site of LuxR homologs is highly degenerated and difficult to predict (Fogg 2019). In line with this notion, a motif discovery search conducted on the 105 regulatory regions that correspond to the promoter regions of the 181 DE genes did not reveal a reliable motif sequence. Therefore, to circumvent this problem, we searched for a motif similar to the GtaR binding site because this regulator is closely related to CerM (Figure 1C), and both could recognize somewhat similar sites. Consequently, this strategy would identify the GtaR binding sites that may be part of our data set, as well as those sites recognized by CerM. This search revealed a motif present in *gtaR* and 9 other regulatory regions (Table S5). A LOGO representation of this motif is shown in Figure S11.

**Figure 5:**
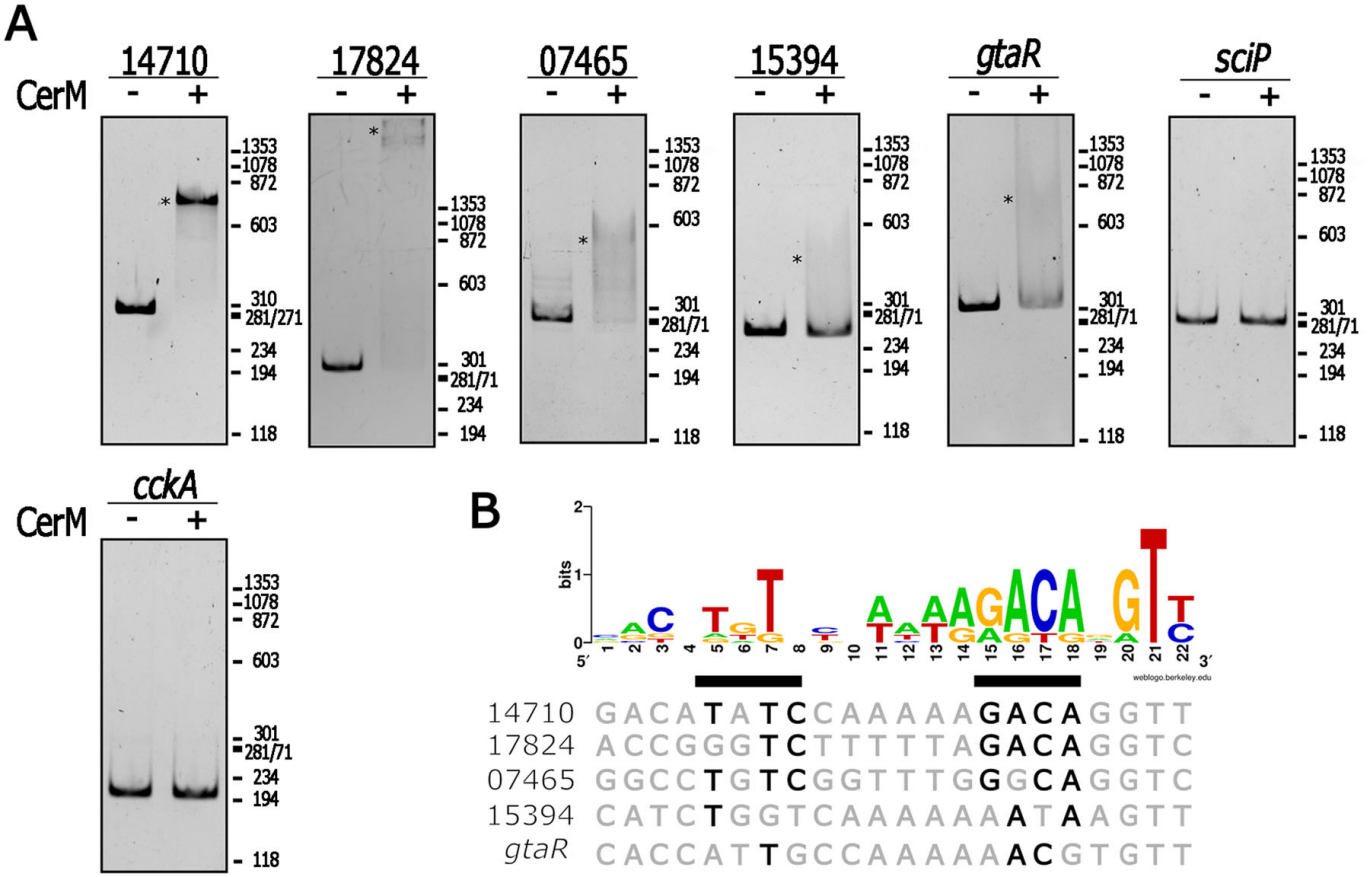
Identification of the CerM binding site. (A) Electrophoretic mobility shift assays of the indicated regulatory regions in the absence or presence of CerM. The reactions included 200 ng of His6X‐CerM (+), or not (−). An asterisk indicates the migration of the CerM–DNA complex. The regulatory region of *sciP* and a fragment of the coding region of *cckA* were used as negative controls. Fragment sizes are: for 14710, 310 bp; for 17824, 306 bp; for 07465, 323 bp; for 15394, 249 bp; for *gtaR*, 337; for *sciP*, 303 bp; for *cckA*, 239 bp. Migration of the HaeIII‐PhiX174 DNA size standards is shown at the right in bp. (B) LOGO sequence of the putative CerM binding site obtained from the alignment of the sequence of the retarded fragments. The nucleotides similar to the logo sequence are shown in bold.

To obtain evidence about the functionality of these sites, we evaluated the possible interaction of five of them with CerM. We observed that the DNA fragments corresponding to the control regions of RSWS8N_14710 (transcription factor, log_2_FC = 5.1), RSWS8N_17824 (transcription factor, log_2_FC = 2.7), RSWS8N_07465 (putative translation factor, log2FC = 3.8) exhibited clear mobility shifts. According to the log_2_FC values from the transcriptomic results, CerM activates these promoters. In contrast, the regulatory regions of RSWS8N_15394 (LuxR regulator, log_2_FC = 5.4), and *gtaR* (LuxR regulator, log_2_FC = −1.58) underwent a weak mobility shift in the presence of CerM (Figure 5A), suggesting that CerM may also act as a repressor. The promoter region of *sciP* (transcriptional regulator controlled by CtrA) and an internal fragment of *cckA* were included as negative controls (Figure 5A). The alignment of the sequences that showed a positive interaction with CerM revealed a fairly conserved motif, **T**N**T**(N5)AAG**ACA**(N2)**T**, which, as expected, resembles the previously reported GtaR binding site (Leung et al. 2012). This motif could represent the CerM binding site (MBS) (Figure 5B).

### 3.8 Mapping the CerM Binding Site

**Figure 6:**
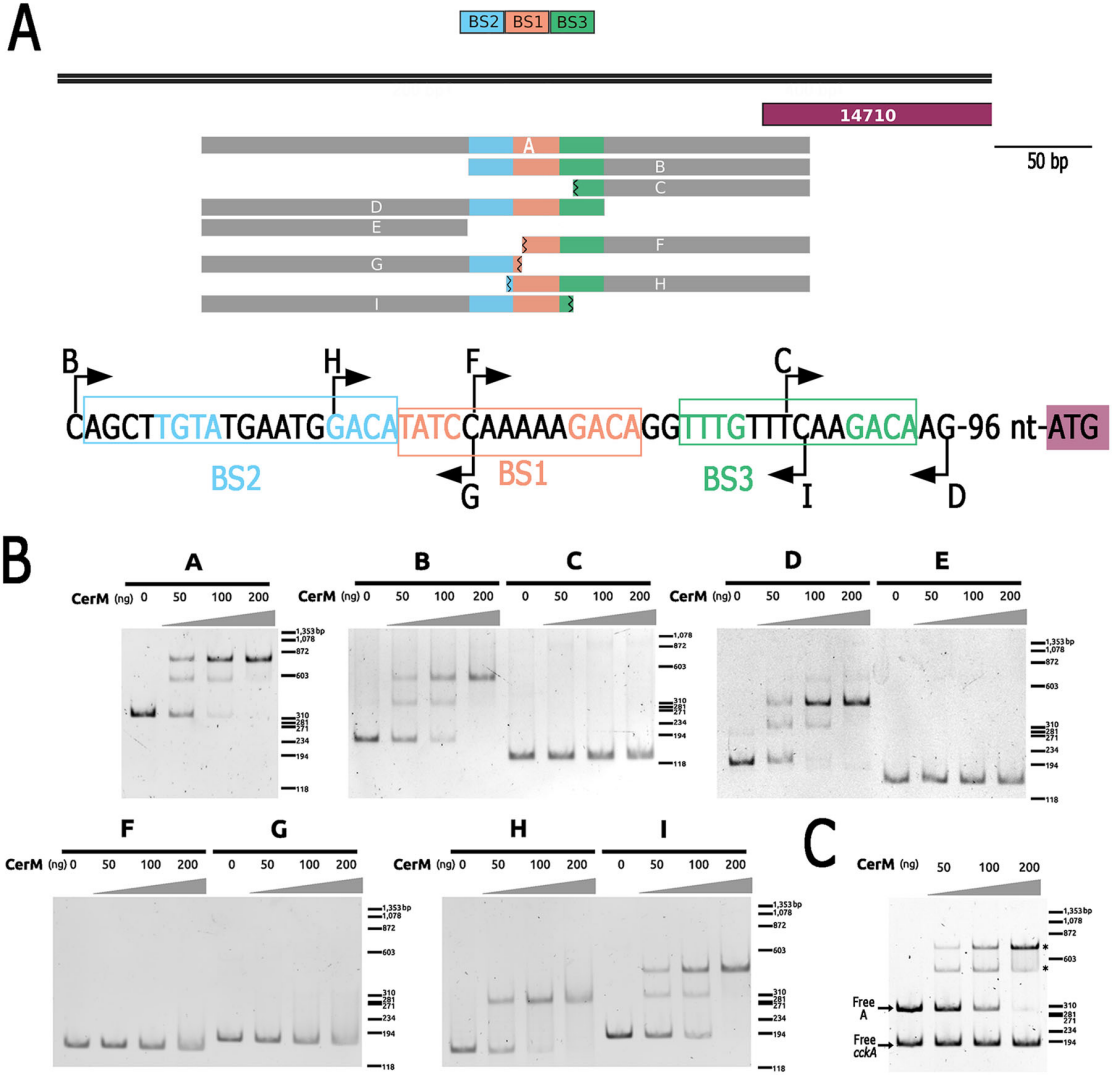
Mapping of the MBS in the control region of RSWS8N_14710. (A) A double horizontal line represents the incomplete coding region of RSWS8N_14710 and 286 bp of its control region; the size scale is indicated on this bar. Below, a purple rectangle indicates where the region of RSWS8N_14710 is located. The PCR fragments tested by EMSA are represented as gray rectangles; they are labeled from A to I. The potential MBS are indicated as blue, orange, and green boxes. An incomplete MBS is represented by a broken line in the color boxes. Below, the enlargement of the region where these sites are located is shown, and the three putative MBS are enclosed in rectangles. The nucleotides in color match the most conserved residues of the putative MBS. (B) Electrophoretic mobility assays of the DNA fragments indicated above each gel, from A to I. In these experiments, increasing amounts of His6X‐CerM were added as indicated. Migration of the molecular weight standards is indicated at the left of each gel. (C) Electrophoretic mobility assay using two different DNA fragments simultaneously: one is a DNA fragment from RSWS8N_14710, and the other is a negative control corresponding to a coding region of *cckA*. These fragments were mixed and incubated in the presence of increased amounts of His6x‐CerM. CerM–DNA complexes are indicated by an asterisk.

To precisely define the region that interacts with CerM, we carried out a deletion analysis of the control region of RSWS8N_14710 (see Figure 6A for a schematic of the different fragments used), starting with the 310 bp region tested in the experiment shown in Figure 5. This region was selected because a stable complex with CerM was observed in the EMSA. Two different complexes were formed when Fragment A and a low concentration of CerM were used (50 ng), but the slow‐migrating complex predominated when the concentration of CerM was increased (200 ng) (Figure 6B,A). The presence of these complexes was not affected by including in the same reaction unspecific DNA (Figure 6C). From these results, we presumed that at least two sites with different affinities for CerM are present in this fragment.

Removing 140 bp of the upstream region of Fragment A (Fragment B) did not affect the binding of CerM, regardless of the concentration of CerM included in the assay (Figure 6B). However, further removal of 41 bp (Fragment C) abolished the binding of CerM (Figure 6B,C). Therefore, the binding site of CerM should be present within this 41 bp region. In agreement with this proposal, the CerM–DNA complexes were also formed when this 41 bp region was included along with the upstream region (Figure 6A, Fragment D, Figure 6B,D). Within this short region of 41 bp, the sequence TwTm(N_6_)GAC is found twice (Figure 6A, BS2, and BS1 see nucleotide sequence), as well as the variation TwTg(N)_6_GAC (BS3), which are similar to the proposed MBS. A DNA fragment carrying BS2 and BS1 and partial BS3 (Figure 6A, Fragment I), showed a migration shift pattern similar to that observed for the 310 bp fragment, that is, two different complexes that vary in their relative abundance depending on the concentration of CerM (Figure 6B,I). In contrast, a DNA region that includes BS3, BS1, and partial BS2 (Figure 6A, Fragment H) only showed a single complex regardless of the concentration of CerM (Figure 6B,H). The fragments that include BS2 or BS3 but not a complete BS1 (Figure 6A, Fragments F and G) did not show a shift in their electrophoretic mobility in the presence of CerM (Figure 6B,F,G). Together, these data suggest that two MBS (BS2 and BS1) are present in the promoter region of RSWS8N_14710, but BS2 is only occupied when BS1 is present. To explore if these MBSs are in a promoter context, we searched for a putative promoter sequence in this fragment using the promoter prediction software SAPPHIRE (Sequence Analyzer for the Prediction of Prokaryote Homology Inferred Regulatory Elements). This search identified one possible hit (TCGGGC (N17) TATGCT, Pval:0.0005), that is, located 41 bp downstream of the BS1, and 44 bp upstream of the ATG, consistent with the idea that CerM activates the expression of RSWS8N_14710.

### 3.9 CerN in the Presence of AHL, Interferes With the Ability of CerM to Bind DNA

**Figure 7:**
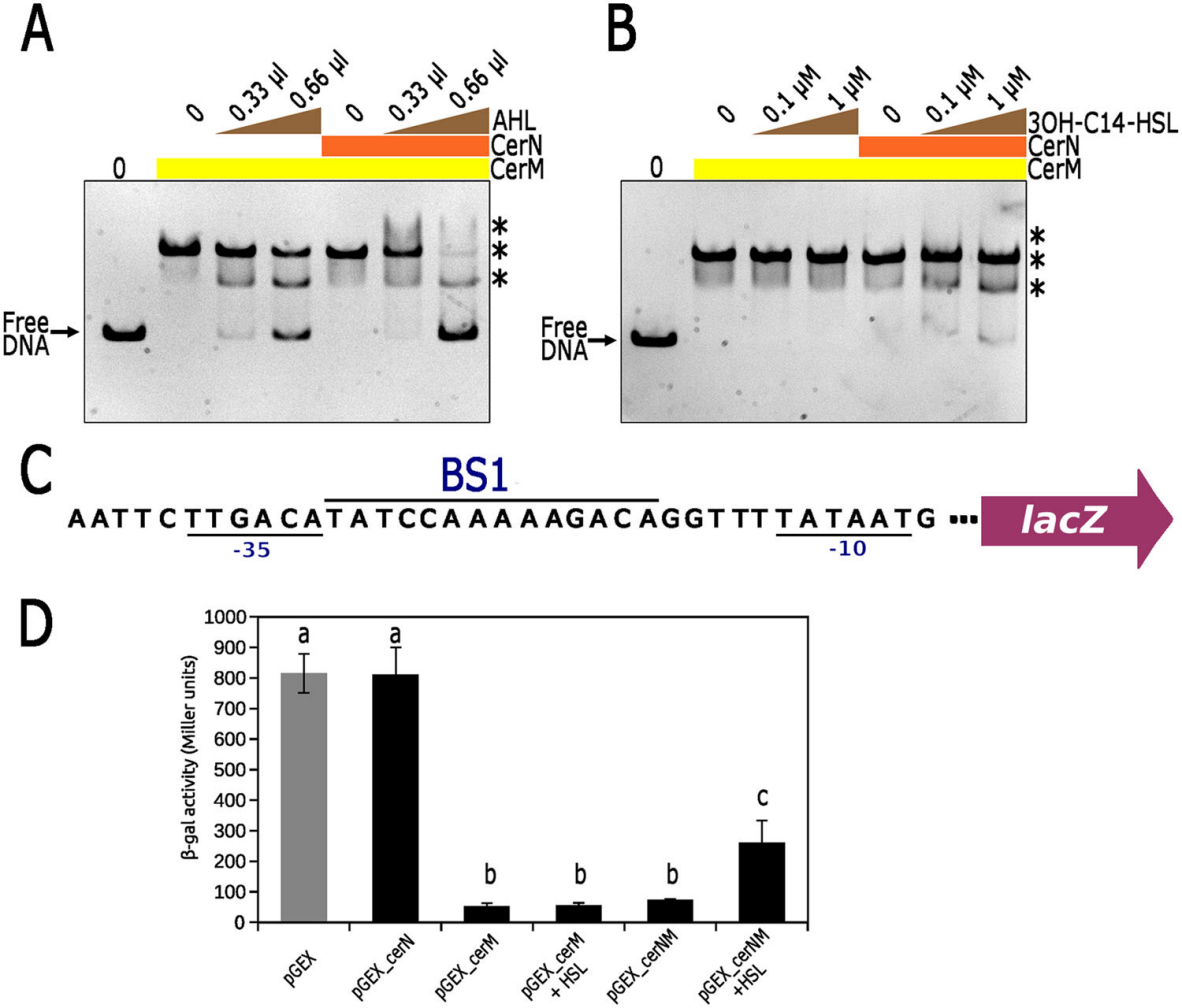
Effect of CerN and the indicated AHLs on the binding of CerM to its DNA target. For both EMSA assays the control region of RSWS8N_14710 of 310 bp was used. The presence of CerM (His6x‐CerM, 200 ng) in the reaction is indicated with a horizontal yellow bar. The inclusion of an equimolar amount of CerN (GST‐CerN) is indicated with a horizontal orange bar. Increasing amounts of C14‐HSL obtained from AM1 supernatants (A), or 3OH‐C14‐HSL (B), are represented with a brown triangle. CerM–DNA complexes are indicated with an asterisk. (C) The nucleotide sequence of the artificial promoter carrying the MBS (BS1) between the conserved − 35 and − 10 elements recognized by the RNA polymerase. (D) β‐galactosidase activities expressed in Miller units. Below each column, the inclusion of AHL and the name of the plasmid is indicated. Data represent mean ± SD (standard deviation). Statistical significance was determined using one‐way ANOVA followed by Tukey's post hoc analysis. Groups sharing the same letter (a, b, c) are not significantly different, while different letters indicate significant differences (*p* < 0.01).

To determine whether CerN and/or the presence of AHL affects the interaction of CerM with its binding site, we examined whether a mixture of these components with CerM impacts the electrophoretic mobility shift of the control region of RSWS8N_14710 (using the Fragment A of 310 bp, illustrated in Figure 6A). As controls, we first verified that, as expected, CerN does not interact with this fragment in the presence or absence of AHL, since it does not have a DNA‐binding domain (Figure S12). Additionally, it was also confirmed that an extract from the cell culture supernatant of the Δ*cerI* strain does not affect the formation of the CerM–DNA complex (Figure S12). Subsequently, we tested the effect of CerN and AHL on the binding of CerM using two different experimental sets. In one set, AHL was obtained from extracts of the culture supernatants of strain AM1 (Figure 7A), and in the other, the synthetic analog 3OH‐C14‐HSL (Cayman Chemical) was used (Figure 7B). These experiments showed that the inclusion of the AHL obtained from extracts of the culture supernatants negatively affected the interaction of CerM with its binding site, increasing the presence of the fast‐migrating complex as well as of free DNA (Figure 7A). The inclusion of CerN by itself did not affect the complex CerM‐DNA in the absence of AHL; however, a substantial amount of free DNA was detected when AHL (0.66 μL) and CerN were included in the reaction (Figure 7A). This suggests that both elements contribute to weakening the interaction of CerM with its binding site. A similar trend was observed when CerN and 3OH‐C14‐HSL were included in the reaction; however, the amount of free DNA and the presence of the fast migration complex were less pronounced than when using the extract from the AM1 supernatant (Figure 7B).

To further support the idea that CerN reduces the ability of CerM to bind its recognition site in the presence of AHL, we assessed the transcriptional activity of the *lacZ* reporter gene controlled by an artificial promoter that includes the proposed MBS (BS1) (TATCCAAAAAGACAG) between the −35 (TTGACA) and −10 (TATAAT) consensus sequences in pcerMBS1::lacZ vector (Figure 7C). In this heterologous system, CerM binding will obstruct the attachment of the RNA polymerase. This plasmid was introduced into *E. coli* MC4100, which was subsequently transformed with another plasmid expressing CerM, CerN, or simultaneously CerM and CerN. β‐galactosidase activity in total cell extracts revealed that the expression of CerN did not affect the basal activity of the artificial promoter, but the expression of CerM severely reduced it. The negative effect caused by CerM was not relieved by the simultaneous expression of CerN. However, the addition of AHL in the culture medium caused an increase in LacZ activity only when CerN and CerM were expressed together (Figure 7D). These results support the idea that the binding of CerM to its recognition site is prevented by the presence of CerN and AHL.

### 3.10 Role of RSWS8N_14710 in Controlling Swimming

**Figure 8:**
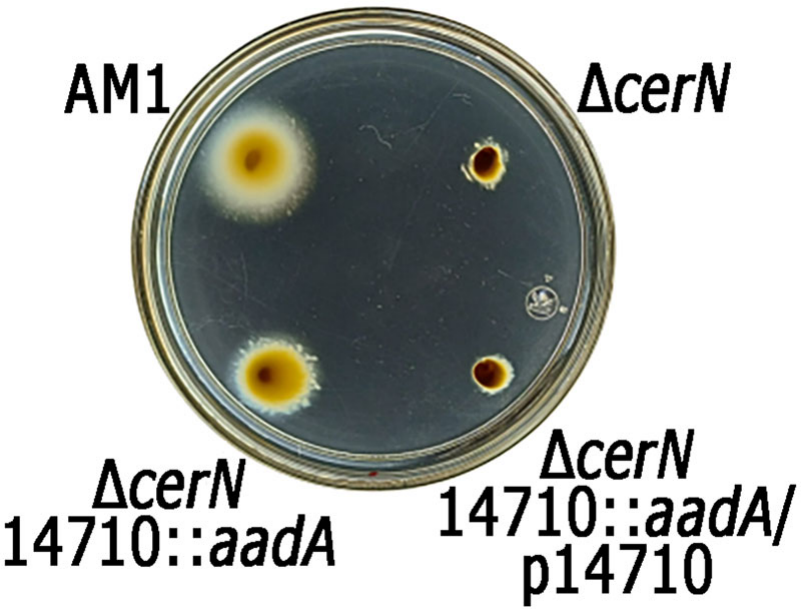
Swimming proficiency of the double mutant Δ*cerN* 14710::*aadA* and the complemented strain Δ*cerN* 14710::*aadA*/p14710. Soft‐agar plates containing Sistrom's minimal medium supplemented with 0.2% casamino acids were inoculated with the indicated strains and incubated for 60 h under photoheterotrophic conditions. JV18 (Δ*cerN::*Hyg), JV33 (Δ*cerN::*Hyg 14710::*aadA*).

Our results indicate that CerM indirectly reduces the expression of the TCS CckA/ChpT/CtrA, suggesting the involvement of an unknown transcription factor. RNAseq experiments revealed that CerM/CerN control the expression of several genes encoding transcription factors. However, only three of them have a putative MBS in their control region and bind CerM. These are *gtaR*, RSWS8N_14710, and RSWS8N_17824. Since GtaR does not affect *fla2* expression, we ponder that the product of the other two genes could have a role in controlling *ctrA*. Based on the better score of its MBS and the stable MBS–CerM complex observed in vitro, we tested if the product of RSWS8N_14710 interconnects the QS system with the TCS CckA/ChpT/CtrA; for this, we isolated the double mutant strain Δ*cerN* RSWS8N_14710::*aadA* and evaluated its swimming ability. In contrast to Δ*cerN*, this strain was able to swim (Figure 8), indicating that the TCS CckA/ChpT/CtrA is active. This result suggests that the transcription factor encoded by RSWS8N_14710 may directly repress the expression of *ctrA* in response to the status of the QS system. It remains to be determined if the product of RSWS8N_17824 also participates in this regulation.

**Figure 9:**
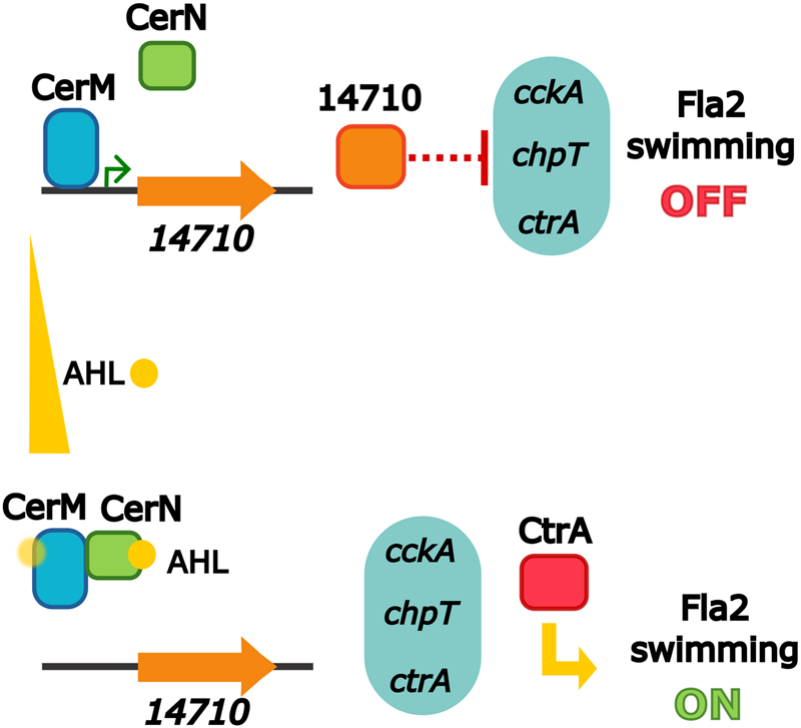
Model of the LuxR‐type regulators CerM and CerN regulating the two‐component system CckA/ChpT/CtrA. The CerN protein, which lacks a DNA‐binding domain, responds to the presence of AHL and the CerN–AHL complex interacts with CerM to promote its dissociation from its DNA‐binding sites. CerM also seems to be able to bind AHL but with less affinity. In the absence of AHL, CerM activates the expression of the RSWS8N_17410 gene, which encodes the probable repressor of the genes that encode the proteins of TCS CckA/ChpT/CtrA.

A model summarizing our main findings is included in Figure 9.

## 4 Discussion

In different bacterial species, the QS system has been shown to control flagellar motility, biofilm formation, virulence, etc. The quorum response has been mainly investigated in species of the γ‐proteobacteria group, particularly in pathogenic bacteria. In contrast, studies in species of the α‐proteobacteria group are much less extensive, despite the plethora of species that belong to this lineage.

In this work, we conducted a comprehensive identification of the QS components of *C. sphaeroides* and determined which of these could be involved in controlling the TCS CckA/ChpT/CtrA, using Fla2 flagellar motility as a readout of the TCS status. Using this approach, we identified two novel components of the QS system of *C*. *sphaeroides*, CerM and CerN, which control the TCS CckA/ChpT/CtrA in response to the AHL synthesized by CerI. We observed that in the absence of CerI or CerN, swimming was severely reduced, but the absence of CerM rescued the wild‐type swimming phenotype of *cerN* and *cerI* single mutants, indicating that in the absence of AHL, CerM is a negative regulator of this trait. As expected, this swimming phenotype mirrors the level of CtrA in these strains. None of the remaining 4 LuxR homologs was required to support swimming or counteract the negative role of CerM, suggesting that these proteins may play a role in controlling another physiological response of *C*. *sphaeroides* or that their role could support the fine‐tuning of the system. Importantly, we showed that CerM and CerN interact with each other in an AHL‐dependent fashion, indicating that CerN could accomplish its role through its conditional interaction with CerM. This result clarifies how a protein without a DNA‐binding domain can be involved in controlling gene expression and provides a molecular explanation for the observed phenotypes of the mutant strains isolated in this work. Given that CerM did not interact with the regulatory region of *cckA*, *chpT*, or *ctrA*, we presumed that the downregulation of CtrA is indirect. This is similar to the situation observed in *Ruegeria* sp. KLH11, where the QS system indirectly controls *ctrA* (Zan et al. 2013), and presumably also occurs in *D*. *shibae* and *R*. *capsulatus*.

To identify the genes regulated by CerM/CerN, the global profiling of Δ*cerN*/pRK_cerM and AM1/pRK415 strains was determined. The analysis of the DE genes between these strains corroborated a reduced expression of *ctrA* and the consequent alteration of the CtrA‐regulon. Importantly, 181 genes were exclusively regulated by CerM/CerN, including genes involved in polysaccharide synthesis, oxidative stress protection, and 9 potential transcription factors besides *ctrA*. Analysis of the regulatory regions of the DE‐expressed genes revealed the presence of a putative MBS in nine regions, suggesting that CerM directly regulates only a few genes. However, the possibility remains that not all the sites that CerM recognizes can be described by the PWM used to discover the possible MBS. Therefore, a more direct approach to identifying these binding sites must be employed in future experiments.

Among the nine genes with an MBS, six of them were positively regulated by CerM, including RSWS8N_14710, suggesting that CerM could mainly act as a transcriptional activator. Most of the LuxR regulators act mainly as transcriptional activators and a few as repressors (Stevens et al. 2011). Nonetheless, it has been shown that depending on the promoter architecture some LuxR activators or repressors could have an opposite role in particular promoters (Egland and Greenberg 2000; Schu et al. 2009; Von Bodman et al. 2003).

Interestingly, at least two LuxR homologs appeared to be controlled by CerN/CerM (RSWS8N_15394 and *gtaR*), indicating the existence of a hierarchical expression pattern that places CerM/CerN at a higher tier.

Considering the information gathered for the nine transcription factors controlled by CerM/CerN, we inferred that only five of them could be involved in controlling the TCS CckA/ChpT/CtrA, and only RSWS8N_14710 and RSWS8N_17824 have promoter regions that interact with CerM. The double mutant Δ*cerN* RSWS8N_14710::*aadA* was able to swim, supporting the idea that the transcription factor encoded by this gene could directly regulate the expression of *ctrA*. Further experiments are underway to demonstrate this possibility and evaluate the possible role of RSWS8N_17824.

Regarding the binding of CerM to its recognition site, our results showed that the regulatory region of RSWS8N_14710 contains at least two MBS, BS1 and BS2. Occupancy of BS2 requires the presence of CerM interacting with BS1. A DNA fragment carrying BS1 binds CerM in vitro and in vivo, supporting the idea that once CerM interacts with BS1, protein–protein interactions favor the binding to BS2. Additional evidence dissecting these sites and measuring their affinity for CerM would support a model to explain this apparent cooperativity.

We propose a model in which CerM binds to its recognition site in the absence of AHL, and CerN‐AHL antagonizes this interaction. However, we noticed in the electrophoretic shift assays that AHL interfered with the binding of CerM, although to a lesser degree than in the presence of CerN‐AHL, suggesting that a high concentration of autoinducer could directly affect CerM and destabilize the CerM–DNA complex. However, it is important to stress that in vivo, a Δ*cerN* strain, which can produce AHL, only recovers *ctrA* expression and consequently swimming proficiency when a secondary mutation inactivates *cerM*. This indicates that in vivo, AHL by itself does not relieve the inhibitory effect of CerM. These opposing results, that is, the EMSA showing that AHL by itself slightly interferes with the CerM–DNA complex, and the in vivo results, could be explained if the intracellular level of AHL does not reach the critical concentration to destabilize the CerM–DNA complex, making CerN‐AHL responsible for regulating CerM. This suggestion presumes that CerM has a lower affinity for AHL than CerN. We hypothesize that the W57F change in the AHL‐binding cavity in CerM could be important for this regulation. In this general scenario, an important set of experiments to test our model will involve isolating point mutants in CerN and CerM that disrupt or improve AHL‐binding and evaluating their functionality.

The LuxR regulator EsaR binds to DNA in the absence of AHL, similarly to CerM. It has been shown that in this form, EsaR represses its own expression while activating the expression of *esaS* (Schu et al. 2009). EsaR has an extended linker region between the AHL‐binding N‐terminal domain and the DNA‐binding domain, as well as an extension at the C‐terminus, which are characteristic signatures of class IV LuxR regulators (Stevens et al. 2011). CerM does not possess these sequence features and cannot be grouped into this subfamily of LuxR regulators.

The negative control of CerN on CerM evokes the inhibition of the LuxR homolog TraR mediated by TrlR in *A. tumefaciens*; TrlR is a truncated version of TraR that lacks the DNA‐binding domain (Oger et al. 1998; Zhu and Winans 1998). In this case, purified TraR bound to AHL (TraR‐AHL) can bind DNA and activate transcription. TrlR‐AHL binds TraR‐AHL, forming inactive heterodimers unable to bind DNA (Chai, Zhu, and Winans 2001; Zhu and Winans 1998). Although in both systems, there are truncated LuxR‐type regulators that control full‐length LuxR regulators in an AI‐dependent fashion, TraR requires AHL to bind DNA while CerM does not. TrlR is highly similar to TraR (88% identity); in contrast, CerM and CerN show only 31% identity, suggesting that CerN is an ancient duplication or that a rapid sequence divergence has occurred.

The FleQ protein is another example of a transcriptional regulator that is controlled by the homologous protein FleT, which lacks the DNA‐binding domain. In this case, the synthesis of FleT drives the formation of hetero‐oligomeric complexes (FleQ/FleT) that modify the affinity of FleQ for its binding sites (Peña‐Sánchez et al. 2009; Poggio et al. 2005). This example suggests that this form of control could be more prevalent than expected, considering the energetic cost associated with it.

From a general point of view, it was surprising that CerR, the first LuxR regulator identified in *C*. *sphaeroides* (Puskas et al. 1997), is not involved in controlling the TCS CckA/ChpT/CtrA, especially because *cerR* is part of the same operon from which *cerI* is expressed. CerR was characterized in strain 2.4.1 of *C*. *sphaeroides,* and it was reported that in response to AHL, prevents cell aggregation (McIntosh et al. 2019; Puskas et al. 1997). In contrast, this phenotype was not observed in WS8N. This discrepancy in the observed phenotypes is likely due to genetic differences between these strains. Given that both strains share the same genes related to the QS system (Table 1), these changes should be subtle.

Another surprising aspect of the QS system of *C*. *sphaeroides* is the conservation of *gtaR* and the loss of *gtaI* compared to its counterpart in *R*. *capsulatus* (Leung et al. 2012). In *R*. *capsulatus*, the AHL synthesized by GtaI (C16‐AHL) alleviates the negative effect that GtaR_*RC*_ exerts on two targets, which are *gafA* (activator protein of the genes required for the formation of the phage particle called gene transfer agent) (Fogg 2019) and *gtaR* (Leung et al. 2012). Furthermore, GtaR_Rc_ negatively affects the expression of CtrA through a mechanism that remains to be clarified. Why *C*. *sphaeroides* conserved GtaR and not GtaI could be related to the possible intercommunication of this bacterium with other species that share its environmental niche, allowing GtaR to respond to exogenous AHL (Case, Labbate, and Kjelleberg 2008). Alternatively, if GtaR binds the AHL synthesized by CerI, GtaR may have a specialized role in the QS response of *C*. *sphaeroides*.

## Author Contributions

**José Hernández Valle:** conceptualization, investigation, validation, visualization, formal analysis, data curation, writing–original draft, writing–review, and editing. **Benjamín Vega‐Baray:** conceptualization, investigation, validation, writing–review, and editing. **Sebastian Poggio:** conceptualization (lead), supervision, writing–review, and editing. **Laura Camarena:** conceptualization (lead), supervision, funding acquisition, administration, writing–original draft, writing–review, and editing.

## Conflicts of Interest

The authors declare no conflicts of interest.

## Ethics Statement

The authors have nothing to report.

## Supporting information

Supporting information.

Supporting information.

Supporting information.

Supporting information.

Supporting information.

Supporting information.

Supporting information.

Supporting information.

Supporting information.

Supporting information.

Supporting information.

Supporting information.

Supporting information.

Supporting information.

Supporting information.

Supporting information.

Supporting information.

Supporting information.

Supporting information.

## Data Availability

Raw sequence data obtained in this work are available through the NCBI BioProject PRJNA1154699: https://www.ncbi.nlm.nih.gov/bioproject/PRJNA1154699.
