# Correction to “CerM and Its Antagonist CerN Are New Components of the Quorum Sensing System in *Cereibacter sphaeroides*, Signaling to the CckA/ChpT/CtrA System”

**DOI:** 10.1002/mbo3.70014

**Published:** 2025-02-03

Hernández‐Valle, J., B. Vega‐Baray, S. Poggio, and L. Camarena. 2024. “CerM and Its Antagonist CerN Are New Components of the Quorum Sensing System in *Cereibacter sphaeroides*, Signaling to the CckA/ChpT/CtrA System.” *MicrobiologyOpen* 13, no. 6: e012. https://doi.org/10.1002/mbo3.70012.

In Results:

Section 3.1, pages 9 and 10: The mention of Figures 2B, 2C, and 2D was meant to indicate Figures 1B, 1C, and 1D, respectively.

Section 3.2, page 12: The sentence “explaining the polar effect of the allele ΔcerR::aadA (JV16 strain in Figures 3 and 4)” should have actually referred to Figures 2 and 3.

We apologize for these errors.

