# Supplementary figures and images for: CerM and Its Antagonist CerN Are New Components of the Quorum Sensing System in Cereibacter sphaeroides, Signaling to the CckA/ChpT/CtrA System
Source: Microbiologyopen. 2024 Dec 18;13(6):e012. doi: 10.1002/mbo3.70012 (PMC11655674; doi:10.1002/mbo3.70012)

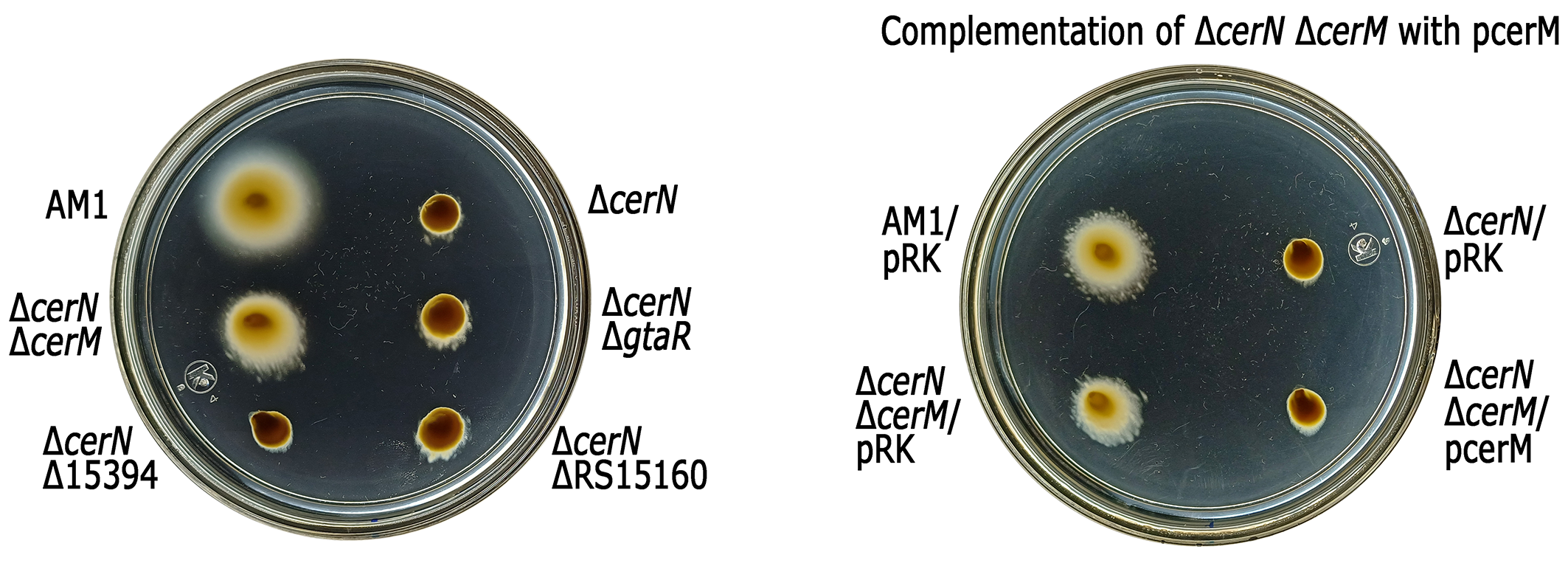

Supplement: Supplementary file 1 — Supporting information. [file MBO3-13-e012-s002.tif]

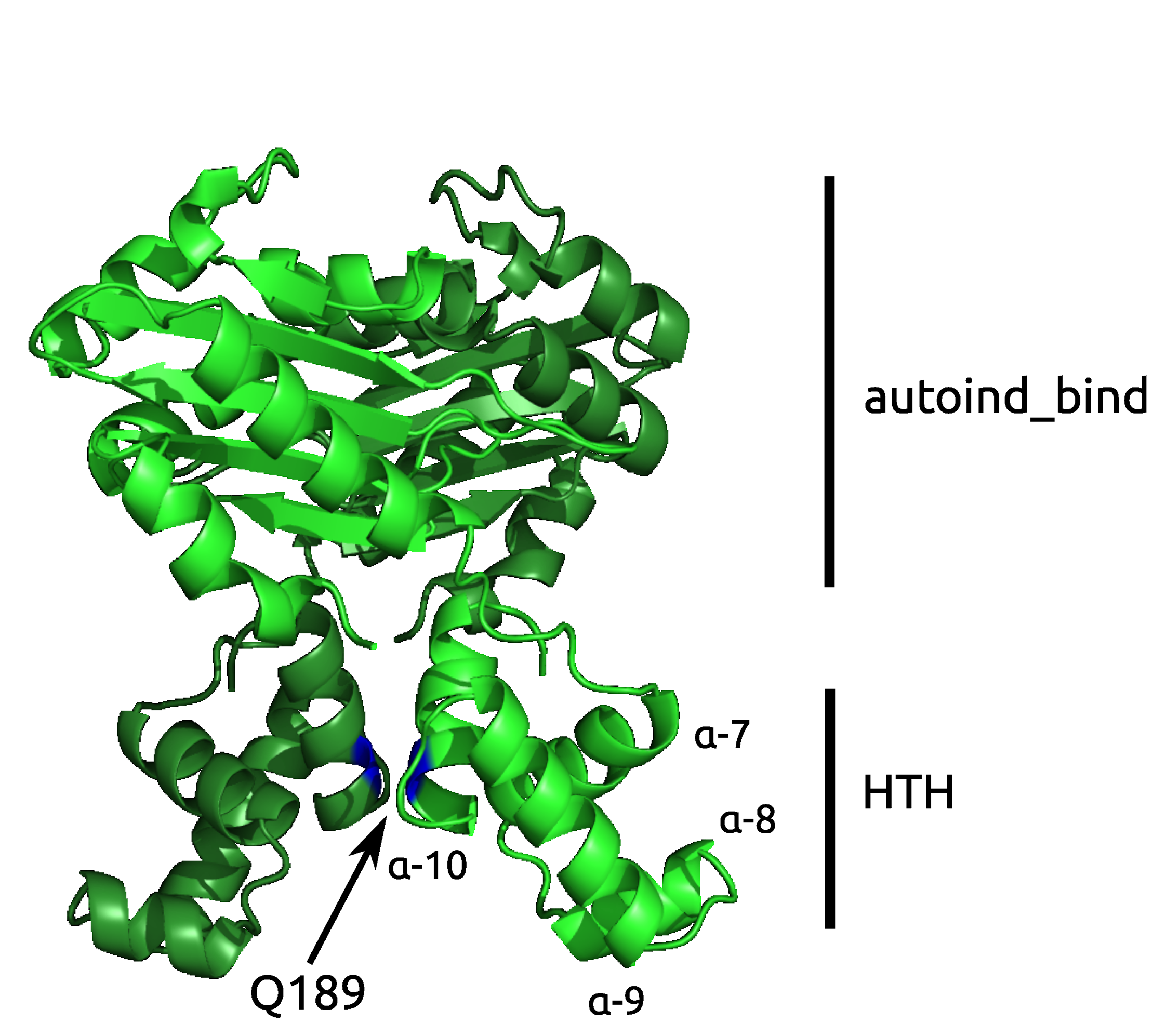

Supplement: Supplementary file 2 — Supporting information. [file MBO3-13-e012-s003.zip › Figure_S2.tif]

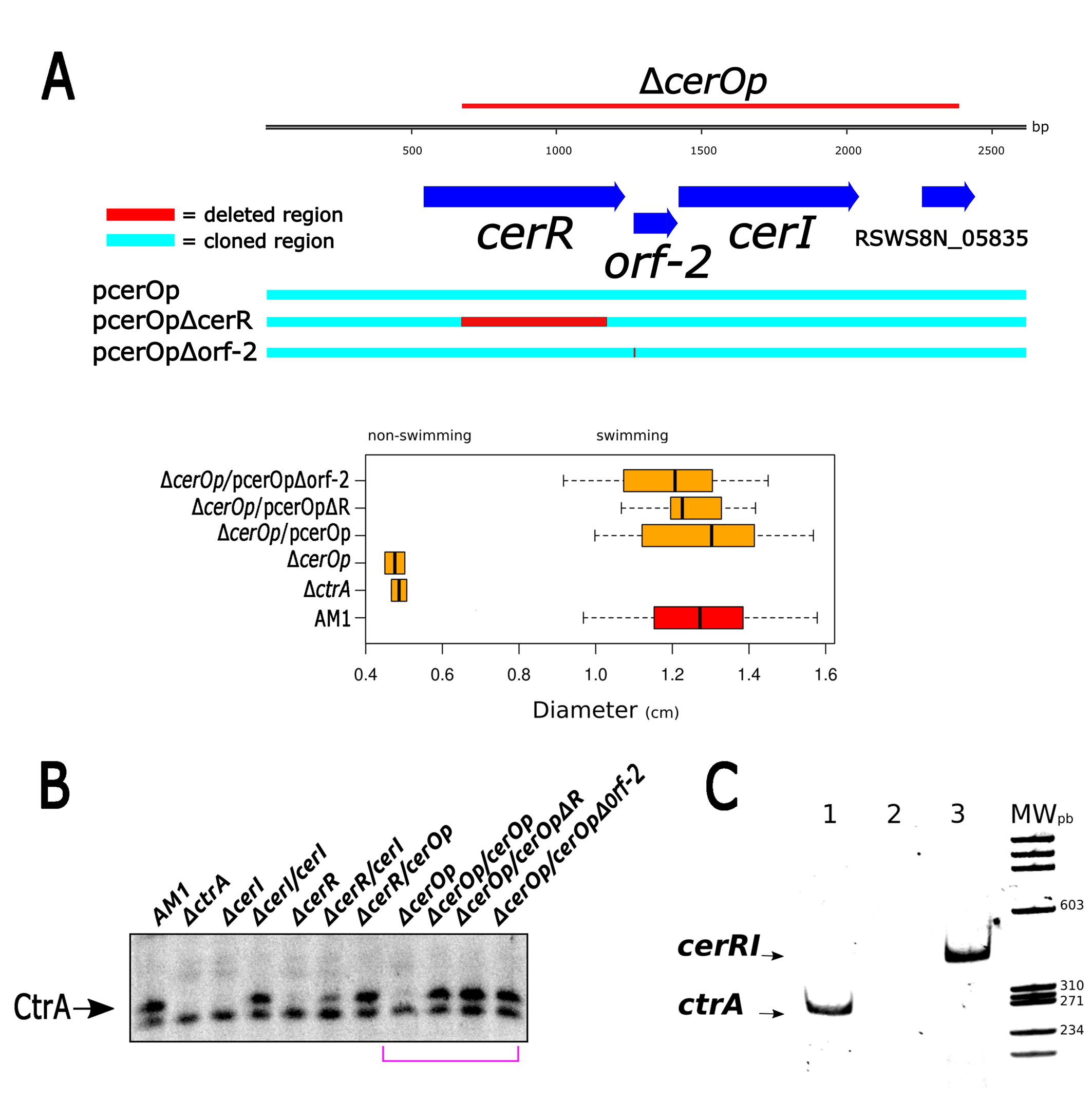

Supplement: Supplementary file 3 — Supporting information. [file MBO3-13-e012-s008.zip › Figure A cerR_2.tif]

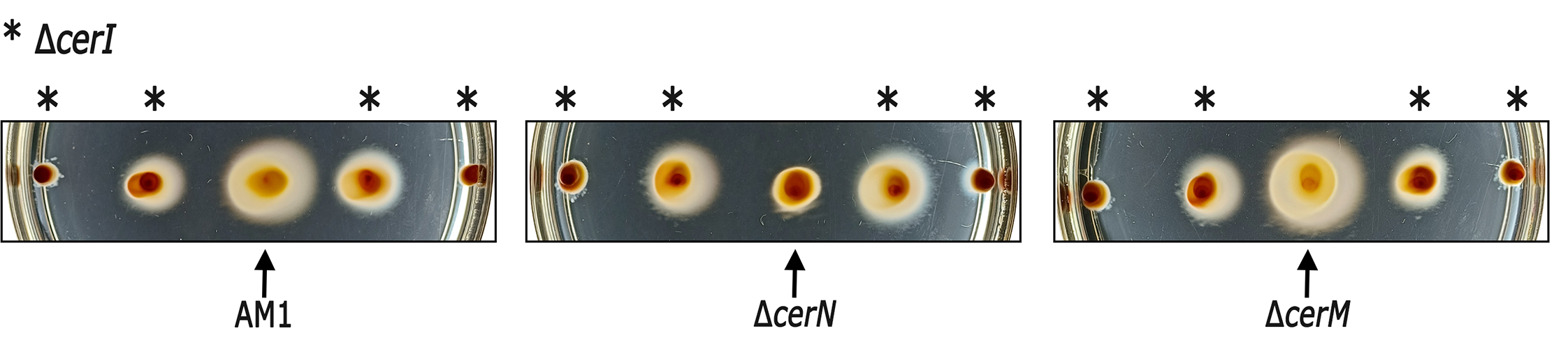

Supplement: Supplementary file 4 — Supporting information. [file MBO3-13-e012-s010.tif]

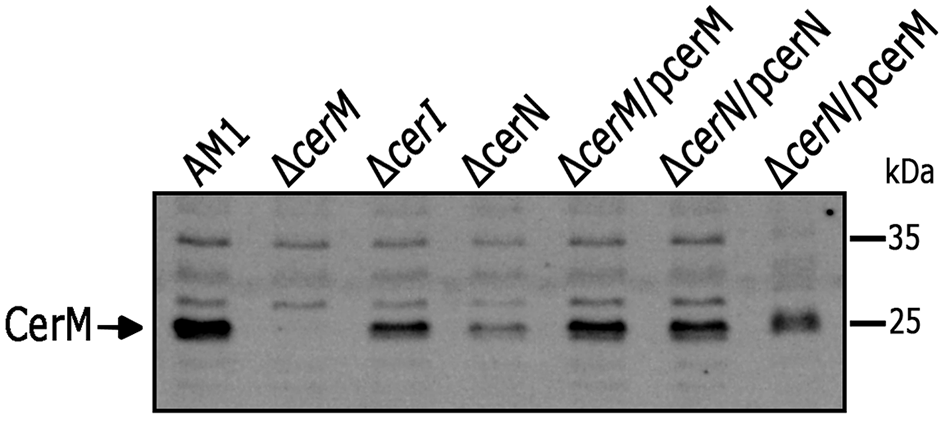

Supplement: Supplementary file 5 — Supporting information. [file MBO3-13-e012-s011.tif]

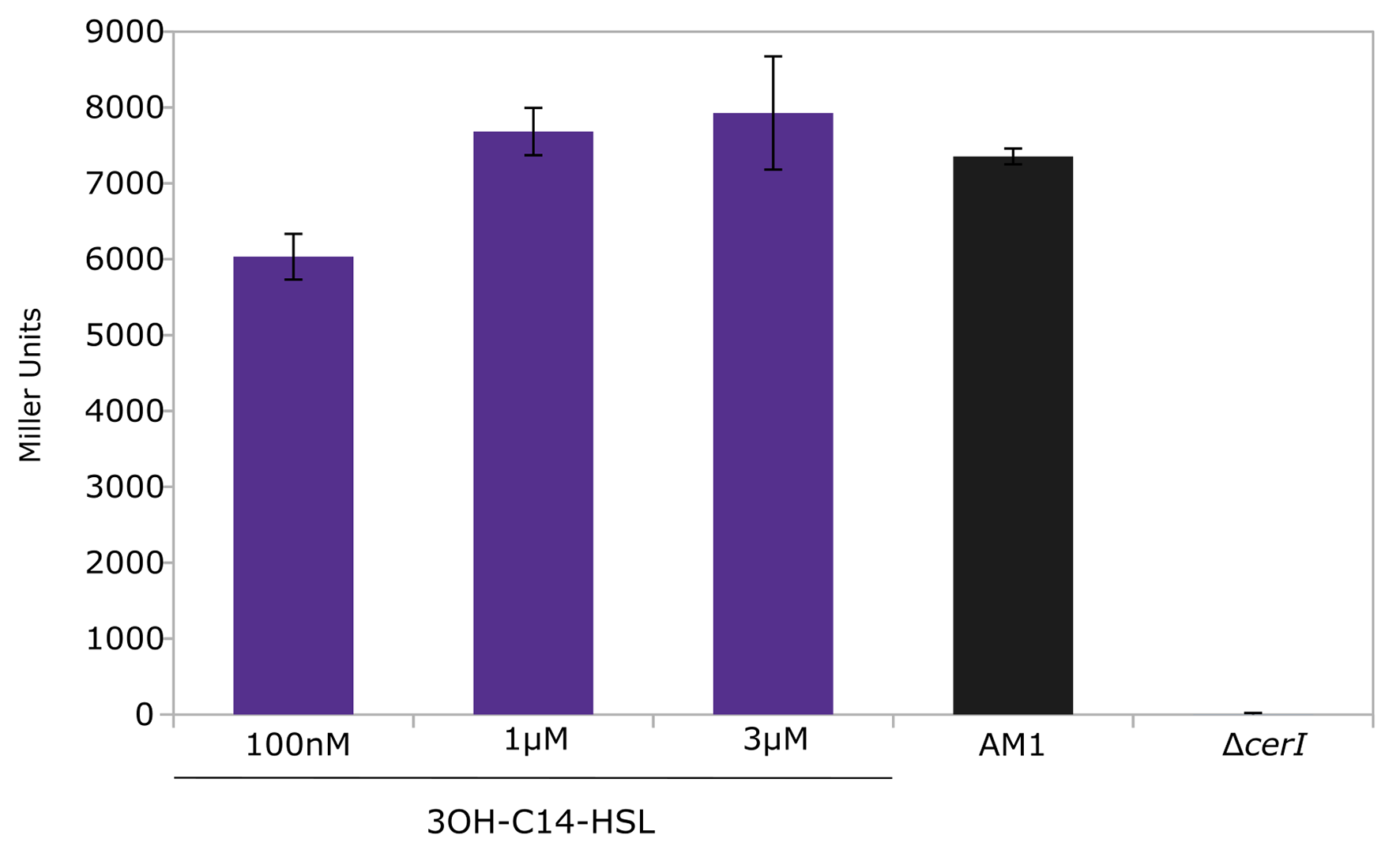

Supplement: Supplementary file 6 — Supporting information. [file MBO3-13-e012-s004.tif]

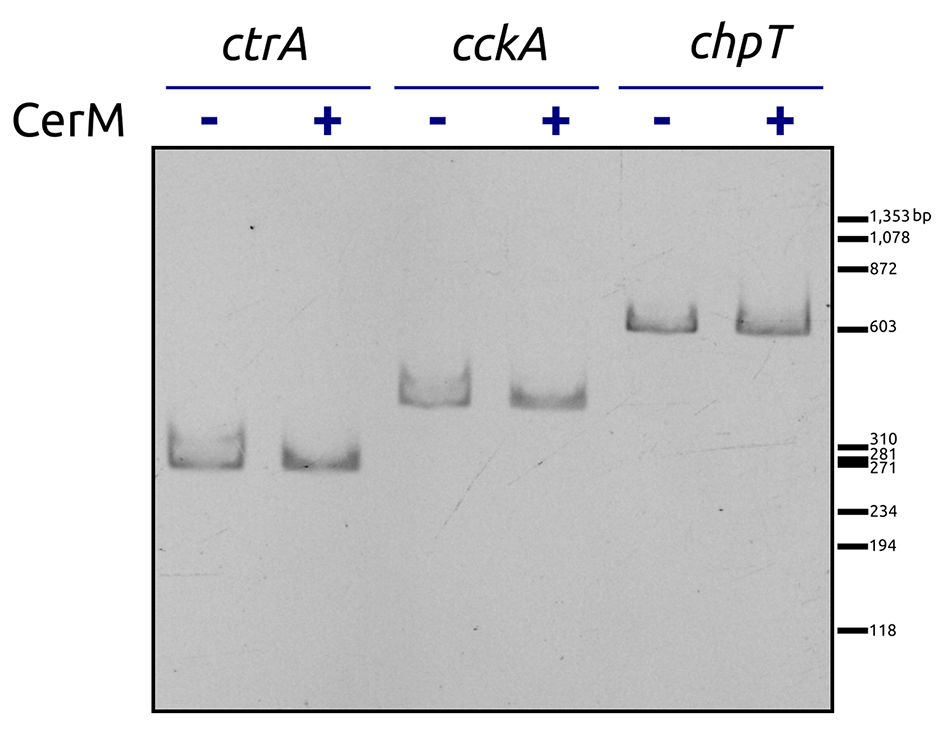

Supplement: Supplementary file 7 — Supporting information. [file MBO3-13-e012-s017.tif]

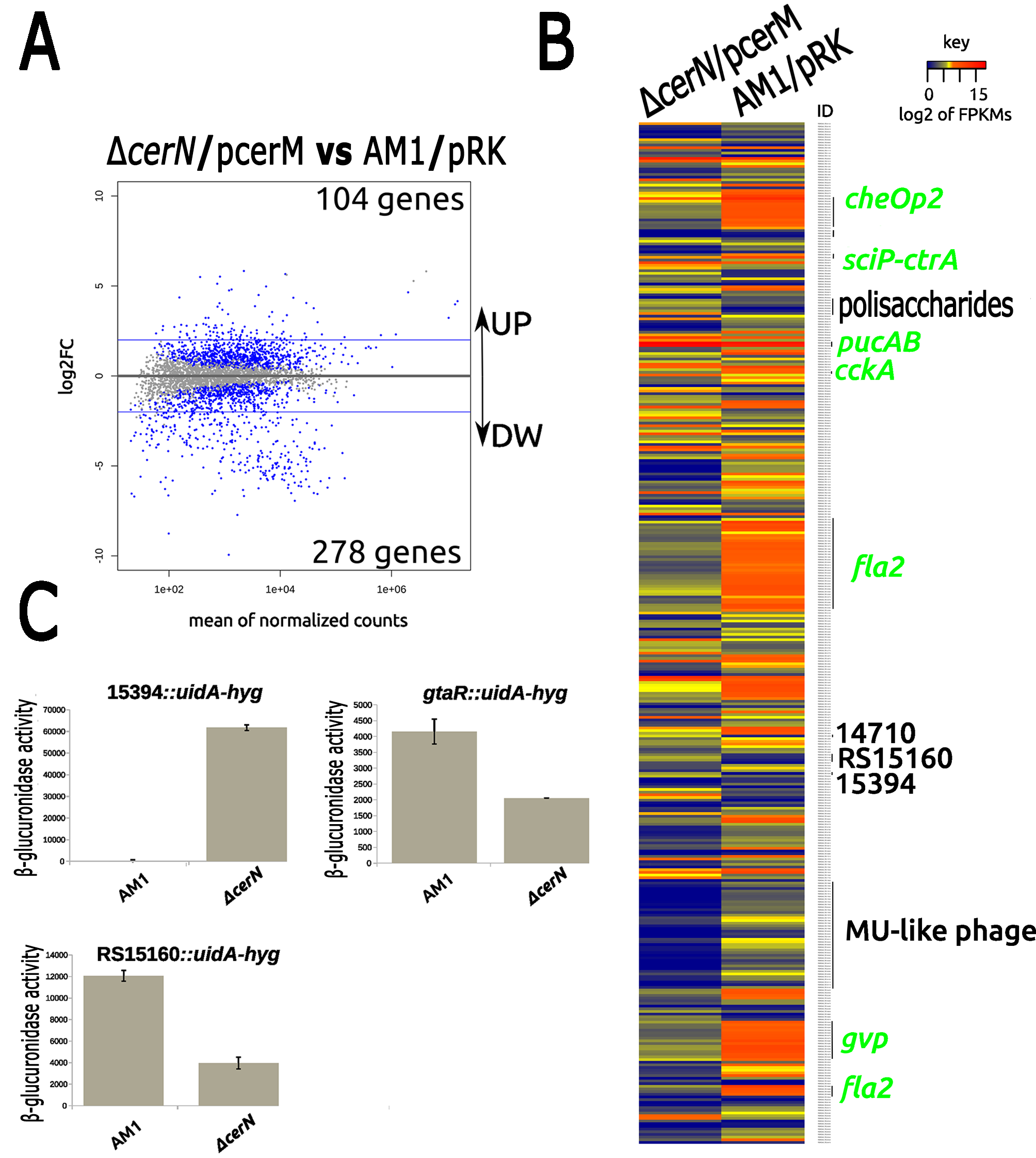

Supplement: Supplementary file 8 — Supporting information. [file MBO3-13-e012-s019.zip › Figure_8.tif]

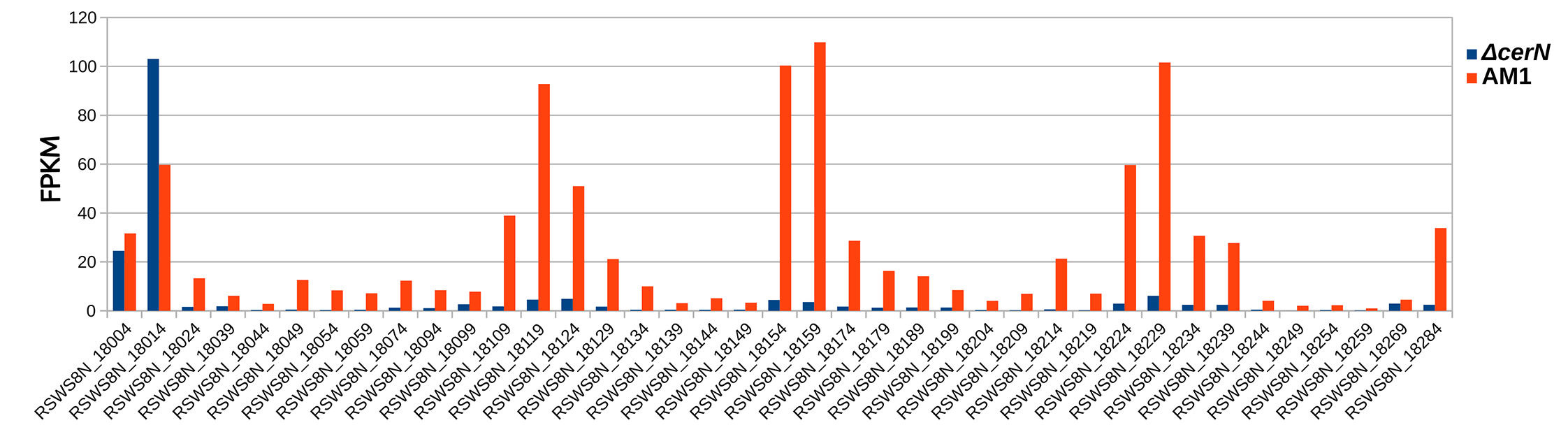

Supplement: Supplementary file 9 — Supporting information. [file MBO3-13-e012-s009.tif]

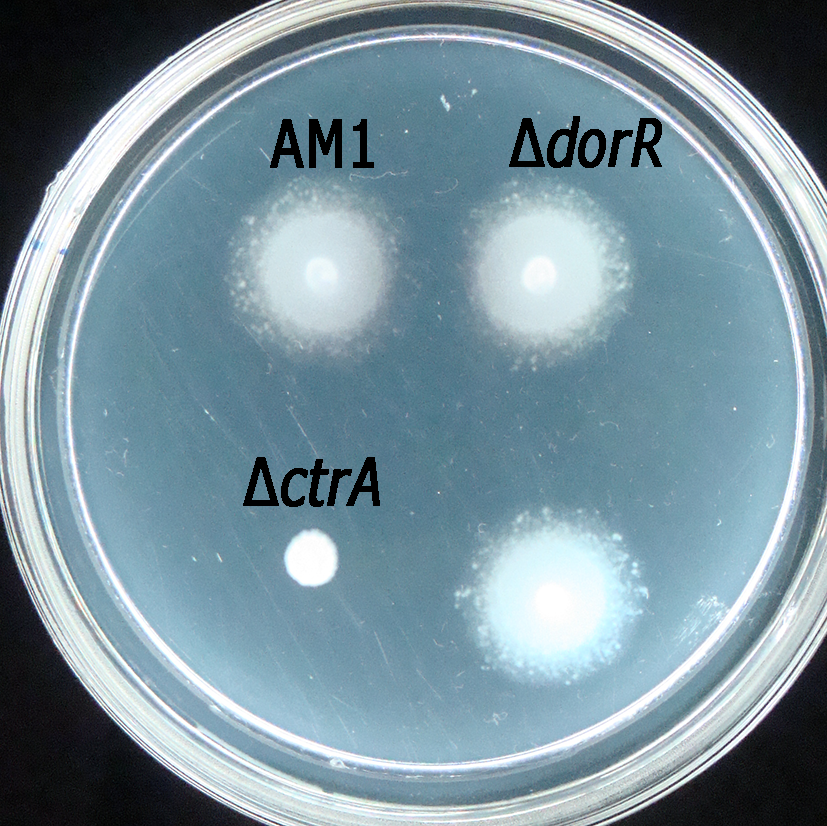

Supplement: Supplementary file 10 — Supporting information. [file MBO3-13-e012-s018.tif]

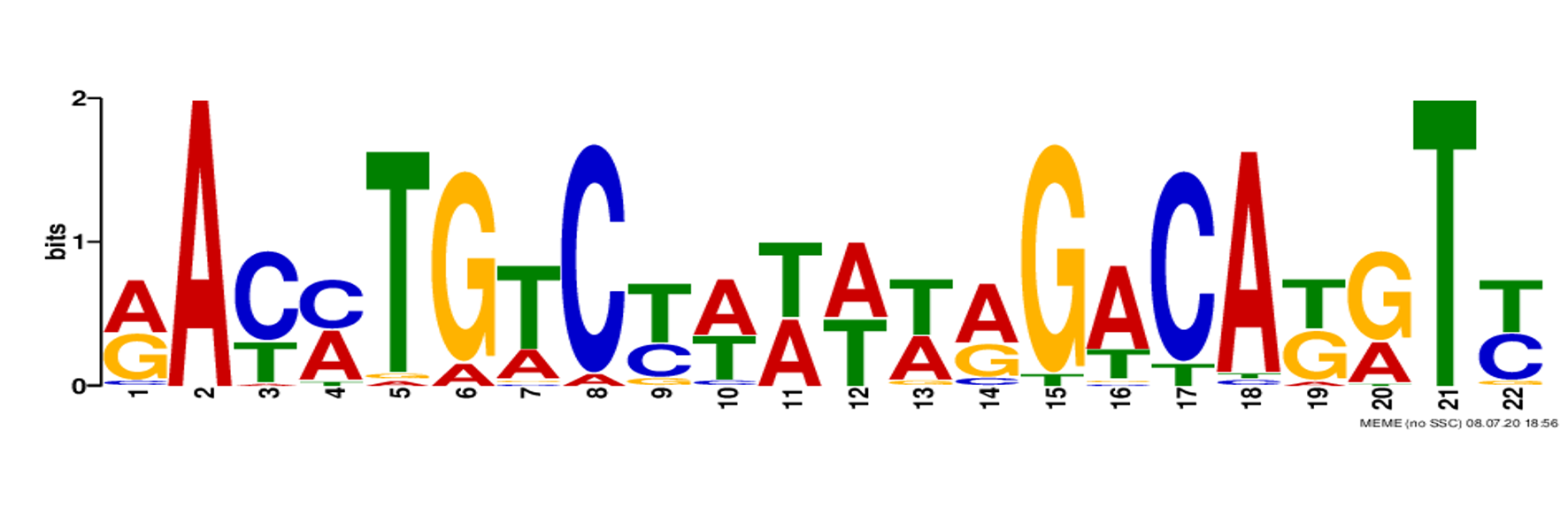

Supplement: Supplementary file 11 — Supporting information. [file MBO3-13-e012-s006.tif]

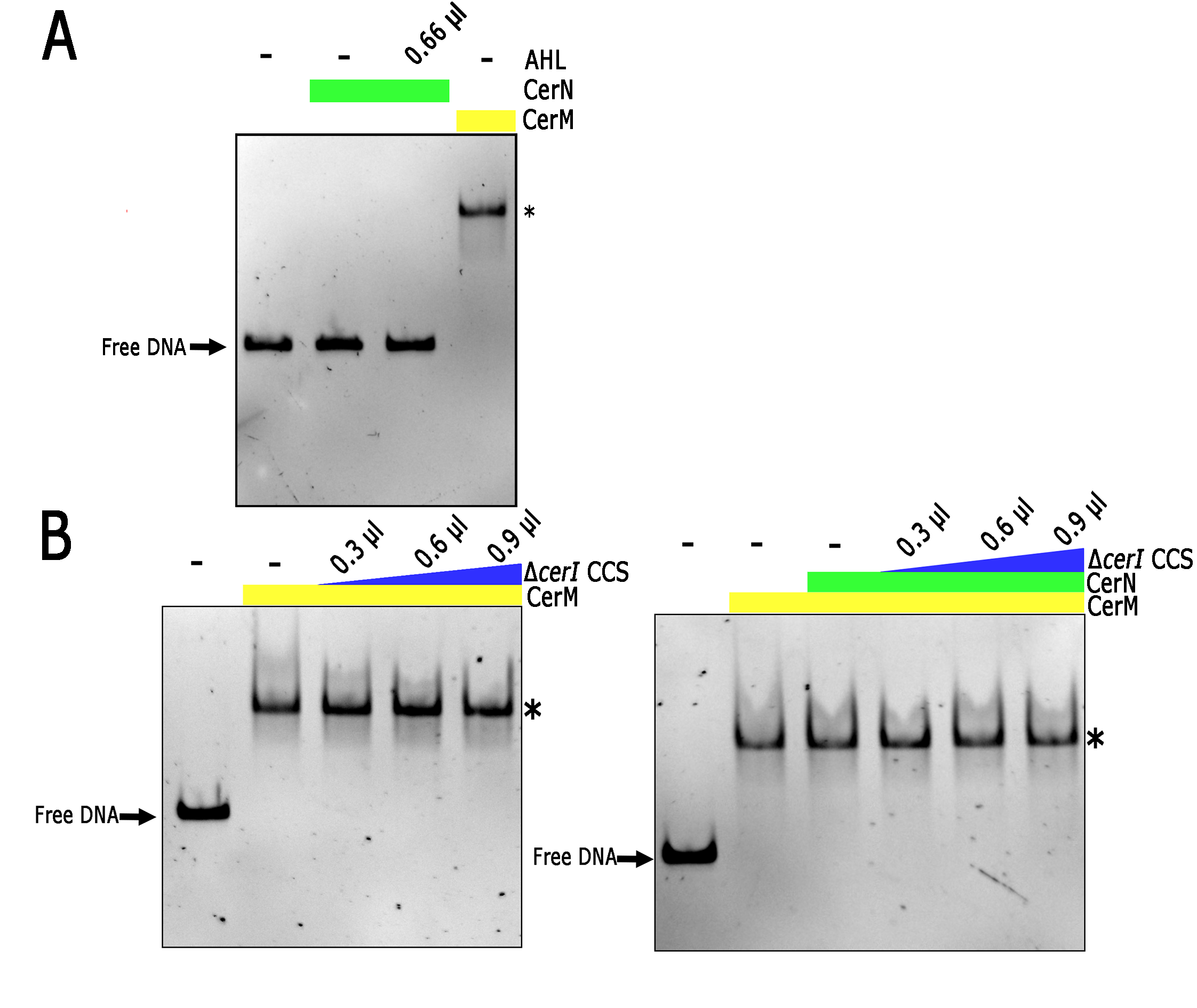

Supplement: Supplementary file 12 — Supporting information. [file MBO3-13-e012-s016.tif]
