# Supplementary material for: CerM and Its Antagonist CerN Are New Components of the Quorum Sensing System in Cereibacter sphaeroides, Signaling to the CckA/ChpT/CtrA System
Source: Microbiologyopen. 2024 Dec 18;13(6):e012. doi: 10.1002/mbo3.70012 (PMC11655674; doi:10.1002/mbo3.70012)
Supplement: Supplementary file 13 — Supporting information. [file MBO3-13-e012-s005.docx]

**Table_A1_M.** Oligonucleotides used in this work.

| **Oligonucleotide** | | | **Sequence** |
| --- | --- | --- | --- |
| CerIfor1 | | | GCTCTAGAGGGCCTTCTGTCGGTGAC |
| CerIRV1 | | | GCTCTAGAAAATGAGCGCACCTTCTCG |
| UPcerRfw | | | GTCTAGAGCTGGACCTTCACCAACCAC |
| UPcerRrv | | | GGAATTCTGGACTCGTTGTCGCATAGG |
| DOWNcerRfw | | | GGAATTCAGAAGCTCGGCACCCTCTC |
| DOWNcerRrev | | | GTCTAGATCGTGACATAGGCGATCGAG |
| 05815DOWNrv | | | GCTCTAGACCGTGTAAGTGATCGTCTCG |
| 05815DOWNfw | | | GCGAATTCGCTGCGTCACTGATTCGTC |
| 05815UPrv | | | GCGAATTCCGAAGTGCCAGTCGGAAT |
| 05815UPfw | | | GCTCTAGAGCATAGGCCATCTGCTTGTA |
| 05820FWup | | | GCTCTAGAAACGGCGAGTCTCAGGATG |
| 05820RVup | | | GCGAATTCCAGGAATGCTTCCTTCATGT |
| 05820FWdown | | | GCGAATTCGAGTCGCGGCATCCTTTA |
| 05820RVdown | | | GCTCTAGACGATGTCCGTCTGCGACT |
| 13935UPfw | | | GCTCTAGACACGAGCAGATCAAGACGAC |
| 13935UPrv | | | GCGAGCTCGTGCAGGCCCAGAAAGTAGC |
| 13935DOWNfw | | | GCGAGCTCAGGAATACCGGCTGATCTGA |
| 13935DOWNrv | | | GCTCTAGAGAGTTGCCGGGGTTGATG |
| UPrvRS15160 | | | GCTCTAGATTCATAATGCACCGTGCCGC |
| UPfwRS15160 | | | GCGAGCTCTTCGCGGTAACTGCAAGAAG |
| DWfwRS15160 | | | GCTCTAGATCACGAAGCGCGAAATCGAC |
| DWrvRS15160 | | | GCGAGCTCATCTATACAGGTCGCTGACG |
| UPfw15394 | | | GCTCTAGATTCAAGCTCGGAACCGGAGC |
| UPrv15394 | | | GCGAATTCCGAGAGGGTTCCACGCTTCA |
| DWfw15394 | | | GCGAATTCTGAAGGCCCGCTTCAAGGCC |
| DWrv15394 | | | GCTCTAGACTTCCTCAGGTCTCTAGGTC |
| fw_compl_5815 | | | GCGAATTCAAGCTTGTGCTGTTCCATTG |
| rv_compl_5815 | | | GCTCTAGAGCCCGACGAATCAGTGAC |
| Rv_cerR_compl | | | GCGAATTCTCACCTCGGATAGACCAGAC |
| Fw_5820_BamH1 | | | GGGATCCCATCTGTCAGACATGAAGG |
| 5815_rv_salI_pGEX | | | GCGTCGACTCAGTGACGCAGCGTCGCGC |
| 5815_fw_ecoRI_pGEX | | | GCGAATTCCCATGGTGAACGTTCCGAGCGT |
| FW_artifprom_CerM | | | AATTCTTGACATATCCAAAAAGACAGGTTTTATAATG |
| RV_artifprom_CerM | | | GATCCATTATAAAACCTGTCTTTTTGGATATGTCAAG |
| T7promBglII | | | GCAGATCTTTAATACGACTCACTATAGGG |
| T7terBglII | | | GCAGATCTGATATAGTTCCTCCTTTCAGC |
| fwUpCerX | | | GGAATTCCGAGCGGCTGTTCTATCTCC |
| rvUpCerX | | | GCTCTAGAGGTGGACAGTTCGTTGCG |
| cerAmutEcoR1 | | | GGAATTCGGCACTTTGAGCCCTTTCCC |
| FwpctrAXbaI | | | CCTCTAGAAAGGTCACGATGATCCAGA |
| RvctrAEcoRI | | | CCGAATTCAGCAGGATGAGATCGTAATC |
| Fw_hygrouniv | | | GCGGATCCCGGGCCAGCTCCGCCATCGCC |
| Rv_hygrouniv | | | GCGGATCCGGCGGCCCGGGGCGTCAGGC |
| Fwcro_Xba | | | GCTCTAGAAGCCGAAGGCCGCGACGCGCG |
| Rvcro_Sac | | | GCGAGCTCCGCCGCCGCGCGAGGATGGCG |
| Fw14710_XbaI | | | GCTCTAGAGGGGTTTTGGCACACAATATG |
| Rv14710_HindIII | | | GCAAGCTTCTAGGCGGCTTCGGAGAGCAC |
| **Oligonucleotides used for EMSAs** | | | |
| **Region** | | **Oligonucleotide** | **Sequence** |
| *cckA*  _(239 bp)_ | | 454A | GTCTAGACGATTTCAGGGCCGAATGTCC |
|  |  | RvCckATMPst | GCCTGCAGCATGAGCCCCCGGGCGATCAGC |
| *cckA*  _(396 bp)_ | | cckAprom | GGTCTAGAGCTTTCCTCGAGCGGACCTCC |
|  |  | RvCckATMPst | GCCTGCAGCATGAGCCCCCGGGCGATCAGC |
| *chpT*  _(603 bp)_ | | ChpMutUp1 | GGATCCAGCAGGAGATGGTCGCAGA |
|  |  | ChpTmutUp2 | GATATCAGATGCGCGAACCCAGAA |
| *sciP*  _(303 bp)_ | | sciPfXbal | CCTCTAGACGAAACCCAGCGAATTGA |
|  |  | RACEsciP146Rv | AGCCCGTGGATCACCGCCTTCACGAC |
| *ctrA*  _(281 bp)_ | | FwpctrAEco | GCGAATTCAAGGTCACGATGATCCAGA |
|  |  | RvpctrASac | GCGAGCTCAGCAGGATGAGATCGTAATC |
| 14710_RR_ | A  (310 bp) | FW14710EbpLargoXbaI | CCCGAATATTCGCGACTTGCA |
|  |  | RV14710Prom | GTGGGCGTCGACGGGATGTTTC |
|  | B  (170 bp) | FW14710GtaRbox | CAGCTTGTATGAATGGACATATC |
|  |  | RV14710Prom | GTGGGCGTCGACGGGATGTTTC |
|  | C  (129 bp) | Fw14710Dbox | TCAAGACAAGTTTTTCTGGAT |
|  |  | RV14710Prom | GTGGGCGTCGACGGGATGTTTC |
|  | D  (191 bp) | FW14710LargoXbaI | CCCGAATATTCGCGACTTGCA |
|  |  | RvGTARbox | CTTGTCTTGAAACAAACCTGTC |
|  | E  (135 bp) | FW14710LargoXbaI | CCCGAATATTCGCGACTTGCA |
|  |  | RV_d5820_BS | GTTCGGCGTCCGCCGCTGTGGA |
|  | F  (147 bp) | FW_CerM_BS | CAAAAAGACAGGTTTGTTTCAA |
|  |  | RV14710Prom | GTGGGCGTCGACGGGATGTTTC |
|  | G  (163 bp) | FW14710LargoXbaI | CCCGAATATTCGCGACTTGCA |
|  |  | RV_CerM_BS | GATATGTCCATTCATACAAGCT |
|  | H  (155 bp) | FW_CerM_BS_F_extend_raso | GACATATCCAAAAAGACAGGTT |
|  |  | RV14710Prom | GTGGGCGTCGACGGGATGTTTC |
|  | I  (182 bp) | FW14710LargoXbaI | CCCGAATATTCGCGACTTGCA |
|  |  | RV_CerM_BS_G_extend | AAACAAACCTGTCTTTTTGGAT |
| 07465  (323 bp) | | FW_07465 | CGCCACATCCGTCGCCATCG |
|  |  | RV_07465 | GAGACAGGCCGAGAACCCAG |
| 15394  (249 bp) | | FW_RR_15394 | GCGAATTCCCATCTGCACATTTTCGGTAAC |
|  |  | UP_RV_15394 | GCGAATTCCGAGAGGGTTCCACGCTTCA |
| gtaR  (337 bp) | | 13935UPrv | GCGAGCTCGTGCAGGCCCAGAAAGTAGC |
|  |  | FW_RR_13935 | GCGAGCTCGTTTCCGGTCTTGGCGATTT |
| 17824  (306 bp) | | FW_RSWS8N_17824 | CCCTCCCCTGATATCGGGAACTCG |
|  |  | RV_RSWS8N_17824 | CGTAAAAATGCCCACCGACCGGAAG |
