# Supplementary material for: CerM and Its Antagonist CerN Are New Components of the Quorum Sensing System in Cereibacter sphaeroides, Signaling to the CckA/ChpT/CtrA System
Source: Microbiologyopen. 2024 Dec 18;13(6):e012. doi: 10.1002/mbo3.70012 (PMC11655674; doi:10.1002/mbo3.70012)
Supplement: Supplementary file 14 — Supporting information. [file MBO3-13-e012-s001.docx]

**Table_A2_M.** Species and accession numbers of the sequences used for predicting GtaR binding sites.

| **Organism** | **Genome (RefSeq)** | **GtaR homologue**  **(Protein ID)** |
| --- | --- | --- |
| *Rubellimicrobium thermophilum* DSM 16684 | GCF_000442315.1 | WP_021098544.1 |
| *Ketogulonicigenium vulgare* WSH-001 | GCF_000223375.1 | WP_014538005.1 |
| *Ketogulonicigenium vulgare* Y25 | GCF_000164885.1 | ADO43647.1 |
| *Wenxinia marina* DSM 24838 | GCF_000836695.1 | KIQ70708.1 |
| *Roseobacter sp.* CCS2 | GCF_000169435.1 | WP_008235250.1 |
| *Paracoccus denitrificans* PD1222 | GCF_000203895.1 | WP_011747126.1 |
| *Paracoccus sp.* J39 | GCF_000518925.1 | [WP_028713550.1](https://www.ncbi.nlm.nih.gov/protein/WP_028713550.1?report=genbank&log$=prottop&blast_rank=1&RID=DD7983G4013) |
| *Paracoccus sp.* J55 | GCF_000513975.1 | [WP_028713550.1](https://www.ncbi.nlm.nih.gov/protein/WP_028713550.1?report=genbank&log$=prottop&blast_rank=1&RID=DD7FE2NC016) |
| *Paracoccus sp.* TRP | GCF_000185925.1 | [WP_010395680.1](https://www.ncbi.nlm.nih.gov/protein/WP_010395680.1?report=genbank&log$=prottop&blast_rank=1&RID=DD7Z8FSZ016) |
| *Paracoccus sp.* N5 | GCF_000371965.1 | [WP_017999581.1](https://www.ncbi.nlm.nih.gov/protein/WP_017999581.1?report=genbank&log$=prottop&blast_rank=1&RID=DD81DR0V013) |
| *Paracoccus aminophilus* JCM 7686 | GCF_000444995.1 | [WP_020951855.1](https://www.ncbi.nlm.nih.gov/protein/WP_020951855.1?report=genbank&log$=prottop&blast_rank=1&RID=DD82Y0EJ013) |
| *Paracoccus pantotrophus* J40 | GCF_000526615.1 | [WP_011747126.1](https://www.ncbi.nlm.nih.gov/protein/WP_011747126.1?report=genbank&log$=prottop&blast_rank=1&RID=DD84CDTT013) |
| *Paracoccus pantotrophus* J46 | GCF_000518905.1 | [WP_011747126.1](https://www.ncbi.nlm.nih.gov/protein/WP_011747126.1?report=genbank&log$=prottop&blast_rank=1&RID=DD85YGFE013) |
| *Paracoccus zeaxanthinifaciens* ATCC 21588 | GCF_000420145.1 | [WP_022708236.1](https://www.ncbi.nlm.nih.gov/protein/WP_022708236.1?report=genbank&log$=prottop&blast_rank=1&RID=DD89C0M0013) |
| *Rhodobacter capsulatus* SB 1003 | GCF_000021865.1 | [WP_013066073.1](https://www.ncbi.nlm.nih.gov/protein/WP_013066073.1?report=genbank&log$=prottop&blast_rank=1&RID=DD8G5MPH013) |
| *Rhodobacter sp.* SW2 | GCF_000176015.1 | [WP_008032655.1](https://www.ncbi.nlm.nih.gov/protein/WP_008032655.1?report=genbank&log$=prottop&blast_rank=1&RID=DD8KCJD5016) |
| *Pseudorhodobacter ferrugineus* DSM 5888 | GCF_000420745.1 | [WP_022704900.1](https://www.ncbi.nlm.nih.gov/protein/WP_022704900.1?report=genbank&log$=prottop&blast_rank=1&RID=DD8N6HTZ016) |
| *Celeribacter baekdonensis* B30 | GCF_000299875.1 | [WP_009574036.1](https://www.ncbi.nlm.nih.gov/protein/WP_009574036.1?report=genbank&log$=prottop&blast_rank=1&RID=DD938710013) |
| *Maritimibacter alkaliphilus* HTCC2654 | GCF_008124775.1 | [EAQ13017.1](https://www.ncbi.nlm.nih.gov/protein/EAQ13017.1?report=genbank&log$=prottop&blast_rank=3&RID=DD9D27YH016) |
| *Roseibacterium elongatum* DSM 19469 | GCF_000590925.1 | [AHM03967.1](https://www.ncbi.nlm.nih.gov/protein/AHM03967.1?report=genbank&log$=prottop&blast_rank=2&RID=DD9HNMS7016) |
| *Jannaschia sp.* CCS1 | GCF_000013565.1 | WP_011453744.1 |
| *Dinoroseobacter shibae* DFL 12 | GCF_000018145.1 | [WP_012176990.1](https://www.ncbi.nlm.nih.gov/protein/WP_012176990.1?report=genbank&log$=prottop&blast_rank=1&RID=DD9TGTPB016) |
| *Oceaniovalibus guishaninsula* JLT2003 | GCF_000299575.1 | [WP_007427612.1](https://www.ncbi.nlm.nih.gov/protein/WP_007427612.1?report=genbank&log$=prottop&blast_rank=1&RID=DD9WPBE7016) |
| *Cereibacter sphaeroides* WS8N | GCF_000212605.1 | [WP_011336955.1](https://www.ncbi.nlm.nih.gov/protein/WP_011336955.1?report=genbank&log$=prottop&blast_rank=1&RID=DD9YKU9Y013) |
