# Supplementary material for: CerM and Its Antagonist CerN Are New Components of the Quorum Sensing System in Cereibacter sphaeroides, Signaling to the CckA/ChpT/CtrA System
Source: Microbiologyopen. 2024 Dec 18;13(6):e012. doi: 10.1002/mbo3.70012 (PMC11655674; doi:10.1002/mbo3.70012)
Supplement: Supplementary file 15 — Supporting information. [file MBO3-13-e012-s015.docx]

**Table_A3_R. Differentially expressed genes.**

| **old_ID** | **prot_ID** | **log2FC** | **padj** | ****CtrA** | **FPKM** | | **COG** | **Name** |
| --- | --- | --- | --- | --- | --- | --- | --- | --- |
|  |  |  |  |  | **DcerN/**  **cerM** | **AM1/**  **pRK** |  |  |
| RSWS8N_00130 | WP_002719132.1 | 2.5987 | 1.12E-16 | #N/A | 179.531 | 29.648 | T | bolA |
| RSWS8N_00195 | WP_002719145.1 | -2.012 | 7.07E-06 | #N/A | 2.6182 | 10.601 | S | - |
| RSWS8N_00215 | WP_002719149.1 | -3.446 | 2.70E-29 | yes | 2.071953 | 22.8162 | S | - |
| RSWS8N_00345 | WP_002719175.1 | -3.087 | 3.29E-05 | #N/A | 0.53391 | 4.8707 | C | - |
| RSWS8N_00350 | WP_002719176.1 | -2.231 | 1.26E-05 | #N/A | 1.93223 | 9.1988 | C | - |
| RSWS8N_00355 | WP_002719177.1 | -2.517 | 9.56E-06 | #N/A | 0.44021 | 2.565 | C | - |
| RSWS8N_00685 | WP_002719243.1 | 2.1962 | 0.0002654 | #N/A | 147.468 | 32.237 | S | - |
| RSWS8N_00855 | WP_002719277.1 | 2.6553 | 1.17E-13 | #N/A | 34.3405 | 5.4508 | H | mtbC |
| RSWS8N_01005 | WP_002719307.1 | 2.3508 | 0.0004662 | #N/A | 7.30511 | 1.44 | S | - |
| RSWS8N_01090 | WP_002719324.1 | 2.1693 | 1.31E-13 | #N/A | 1113.7 | 247.8 | H | metF |
| RSWS8N_01110 | WP_002719328.1 | 3.29655 | 2.56E-27 | yes | 15.39803 | 1.56893 | H | gabT |
| RSWS8N_01115 | WP_002719329.1 | -3.3725 | 2.92E-17 | yes | 4.963541 | 51.7957 | L | dprA |
| RSWS8N_01155 | WP_037087423.1 | 4.4277 | 2.58E-08 | #N/A | 9.83111 | 0.4572 | L | - |
| RSWS8N_01190 | WP_002719344.1 | 2.1262 | 3.77E-08 | #N/A | 34955 | 8015 | #N/D | #N/D |
| RSWS8N_01315 | WP_002719369.1 | 2.0073 | 4.56E-11 | #N/A | 791.969 | 197.19 | M | tmpC |
| RSWS8N_01355 | WP_002719377.1 | -2.6459 | 7.39E-26 | yes | 14.00105 | 88.0088 | H | msbB |
| RSWS8N_01360 | WP_002719378.1 | -4.5132 | 7.20E-45 | yes | 10.61036 | 242.827 | N | fliG2 |
| RSWS8N_01395 | WP_002719385.1 | -2.525 | 9.78E-29 | #N/A | 2.52738 | 14.525 | G | glgX |
| RSWS8N_01400 | WP_037086806.1 | -2.449 | 9.10E-09 | #N/A | 2.02552 | 11.239 | H | glgC |
| RSWS8N_01905 | WP_002719487.1 | -2.3062 | 4.27E-14 | yes | 9.285266 | 46.2555 | K | - |
| RSWS8N_02125 | WP_002719531.1 | -2.047 | 0.0044554 | #N/A | 1.77903 | 7.4111 | P | katE |
| RSWS8N_02215 | WP_002719549.1 | 4.048 | 1.85E-13 | #N/A | 1056.02 | 63.879 | C | ccpA |
| RSWS8N_02220 | WP_002719550.1 | 2.1061 | 0.0025988 | #N/A | 30.4926 | 7.0915 | V | - |
| RSWS8N_02290 | WP_002719564.1 | -3.0997 | 6.97E-78 | yes | 105.6751 | 906.66 | K | rpoHI |
| RSWS8N_02375 | WP_002719581.1 | 2.0195 | 0.0013135 | #N/A | 12.6404 | 3.1179 | H | cobS |
| RSWS8N_02395 | WP_002719585.1 | -3.8655 | 5.10E-34 | yes | 28.12154 | 409.85 | S | omp28 |
| RSWS8N_02400 | WP_002719586.1 | -2.5964 | 4.52E-18 | yes | 134.3761 | 813.365 | H | metY |
| RSWS8N_02405 | WP_002719587.1 | -5.232 | 1.27E-53 | yes | 93.02619 | 3503.86 | #N/D | #N/D |
| RSWS8N_02410 | WP_002719588.1 | -4.2914 | 6.61E-81 | yes | 657.3507 | 12884 | T | cheY2 |
| RSWS8N_02415 | WP_002719589.1 | -4.1041 | 5.64E-95 | yes | 119.05 | 2049.21 | H | cheR1 |
| RSWS8N_02420 | WP_002719590.1 | -4.3819 | 7.53E-96 | yes | 41.84629 | 874.37 | NT | cheW1 |
| RSWS8N_02425 | WP_002719591.1 | -5.7871 | 6.36E-170 | yes | 18.37415 | 1016.71 | H | cheA1 |
| RSWS8N_02430 | WP_002719592.1 | -5.6972 | 3.88E-115 | yes | 22.0524 | 1153.07 | T | cheY1 |
| RSWS8N_02435 | WP_002719593.1 | -5.0249 | 6.23E-176 | yes | 30.30057 | 989.482 | #N/D | cheX |
| RSWS8N_02440 | WP_082242019.1 | -5.171 | 6.52E-108 | #N/A | 25.4487 | 921.11 | NT | cheD |
| RSWS8N_02445 | WP_002719595.1 | -5.826 | 4.14E-62 | yes | 22.10171 | 1255.7 | T | mcpA |
| RSWS8N_02450 | WP_082242020.1 | -6.9639 | 2.56E-129 | yes | 3.473646 | 438.221 | NT | tlpS |
| RSWS8N_02455 | WP_002719597.1 | -6.1764 | 3.56E-180 | yes | 10.71797 | 778.026 | NT | mcpB |
| RSWS8N_02460 | WP_002719598.1 | -5.3541 | 5.45E-78 | yes | 8.328383 | 344.063 | T | cheY5 |
| RSWS8N_02465 | WP_002719599.1 | -4.1509 | 5.26E-21 | yes | 9.633956 | 168.576 | S | - |
| RSWS8N_02565 | WP_002719619.1 | -3.0538 | 0.0063539 | yes | 0.691888 | 5.9853 | - | - |
| RSWS8N_02585 | WP_011840821.1 | -3.3858 | 0.00015198 | yes | 0.153192 | 1.57827 | S | - |
| RSWS8N_02590 | WP_002719624.1 | -4.8771 | 1.42E-07 | yes | 0.050837 | 1.3144 | S | - |
| RSWS8N_02710 | WP_002719648.1 | 2.729 | 1.91E-12 | #N/A | 41.9982 | 6.3163 | #N/D | #N/D |
| RSWS8N_02995 | WP_002719705.1 | 2.5935 | 6.05E-19 | #N/A | 86.3692 | 14.317 | S | - |
| RSWS8N_03025 | WP_002719711.1 | -2.102 | 7.83E-19 | #N/A | 16.8963 | 72.687 | D | exoP |
| RSWS8N_03035 | WP_002719713.1 | -2.8705 | 2.01E-10 | yes | 1.487497 | 11.1613 | M | exoA |
| RSWS8N_03040 | WP_002719714.1 | -3.015 | 1.55E-13 | #N/A | 2.4411 | 20.027 | M | exoL |
| RSWS8N_03050 | WP_002719716.1 | -2.274 | 0.0077035 | #N/A | 0.5614 | 2.6272 | GM | sqdC |
| RSWS8N_03315 | WP_002719769.1 | -4.6957 | 2.71E-32 | yes | 7.860915 | 207.156 | S | sciP |
| RSWS8N_03320 | WP_002719770.1 | -3.664 | 1.19E-19 | #N/A | 170.295 | 2160.8 | K | ctrA |
| RSWS8N_03435 | WP_002719793.1 | -2.579 | 2.55E-26 | yes | 8.282235 | 49.8008 | G | eda |
| RSWS8N_03660 | WP_002719838.1 | 2.0209 | 2.21E-10 | #N/A | 2514.78 | 620.15 | #N/D | #N/D |
| RSWS8N_03940 | WP_002719894.1 | 2.10924 | 2.42E-06 | yes | 181.8343 | 42.1955 | - | - |
| RSWS8N_RS21230* | WP_082242035.1 | 2.1057 | 1.52E-10 | #N/A | 140.135 | 32.594 | E | - |

| RSWS8N_04320 | WP_002719970.1 | 2.0271 | 3.83E-08 | #N/A | 11.0204 | 2.713 | I | ispD |
| --- | --- | --- | --- | --- | --- | --- | --- | --- |
| RSWS8N_04900 | WP_002720086.1 | 4.8573 | 1.29E-20 | #N/A | 79.2112 | 2.7282 | Q | - |
| RSWS8N_05085 | WP_002720123.1 | 2.0807 | 0.000381 | #N/A | 13.1087 | 3.1073 | K | - |
| RSWS8N_05330 | WP_037086972.1 | -2.1063 | 1.13E-07 | yes | 26.65272 | 114.906 | T | - |
| RSWS8N_05635 | WP_235697896.1 | -3.2366 | 0.0002665 | yes | 0.149652 | 1.50348 | #N/D | #N/D |
| RSWS8N_05640 | WP_002720234.1 | -2.4406 | 5.93E-15 | yes | 46.39785 | 252.147 | T | - |
| RSWS8N_05645 | WP_002720235.1 | -2.3088 | 4.81E-10 | yes | 129.6173 | 643.056 | S | - |
| RSWS8N_t15161 |  | 2.5303 | 0.0001682 | #N/A | 69.205 | 11.97 | #N/D | #N/D |
| RSWS8N_05940 | WP_002720294.1 | -2.589 | 2.45E-27 | #N/A | 3.33997 | 20.155 | S | - |
| RSWS8N_05945 | WP_002720295.1 | -3.409 | 2.87E-18 | #N/A | 0.6339 | 6.666 | S | - |
| RSWS8N_05960 | WP_002720298.1 | 2.4336 | 1.46E-09 | #N/A | 38.4325 | 7.1234 | I | - |
| RSWS8N_05965 | WP_002720299.1 | 2.4862 | 3.45E-10 | #N/A | 63.6795 | 11.378 | C | MA20_2 |
| RSWS8N_05975 | WP_002720301.1 | 3.3827 | 3.42E-22 | #N/A | 46.0364 | 4.4113 | M | - |
| RSWS8N_05980 | WP_002720302.1 | 3.791 | 1.23E-11 | #N/A | 14.392 | 1.0385 | M | - |
| RSWS8N_05985 | WP_002720303.1 | 4.8204 | 4.60E-38 | #N/A | 52.5402 | 1.8605 | M | exoB |
| RSWS8N_05990 | WP_082242044.1 | 4.9405 | 1.90E-26 | #N/A | 203.898 | 6.6459 | M | - |
| RSWS8N_06040 | WP_002720314.1 | -4.8911 | 4.28E-69 | yes | 3.067769 | 92.6046 | N | fliM2 |
| RSWS8N_06115 | WP_002720329.1 | 2.3023 | 8.48E-18 | #N/A | 193.154 | 39.198 | E | amaB |
| RSWS8N_06215 | WP_002720349.1 | -3.14 | 8.13E-12 | #N/A | 2.83696 | 25.729 | S | - |
| RSWS8N_06370 | WP_002720380.1 | -3.319 | 0.0001577 | #N/A | 1.3793 | 14.97 | #N/D | #N/D |
| RSWS8N_06450 | WP_002720395.1 | -3.4362 | 1.90E-07 | yes | 0.697262 | 7.80656 | N | motB1 |
| RSWS8N_06455 | WP_002720396.1 | -4.5223 | 5.58E-38 | yes | 1.907819 | 42.7678 | N | motA1 |
| RSWS8N_06460 | WP_002720397.1 | -5.9771 | 1.20E-246 | yes | 10.21993 | 646.044 | NT | tlpL |
| RSWS8N_06500 | WP_002720405.1 | 2.5772 | 3.73E-15 | #N/A | 167.671 | 28.136 | O | - |
| RSWS8N_06595 | WP_002720424.1 | 2.35808 | 1.78E-09 | yes | 3942.886 | 769.638 | S | pufQ |
| RSWS8N_06625 | WP_037087034.1 | 2.0659 | 6.91E-10 | yes | 202.4714 | 48.4036 | F | crtE |
| RSWS8N_06870 | WP_002720479.1 | 2.2994 | 6.17E-17 | yes | 255543.9 | 51957 | H | pucB1 |
| RSWS8N_06875 | WP_002720480.1 | 2.25569 | 8.85E-16 | yes | 128064.1 | 26839 | H | pucA1 |
| RSWS8N_07350 | WP_002720573.1 | -2.3533 | 8.78E-22 | yes | 7.69748 | 39.4675 | S | - |
| RSWS8N_07355 | WP_002720574.1 | -5.3178 | 1.64E-56 | yes | 6.12857 | 246.653 | T | - |
| RSWS8N_07360 | WP_002720575.1 | -4.8404 | 6.66E-50 | yes | 79.32804 | 2276.59 | H | - |
| RSWS8N_07375 | WP_002720578.1 | 2.9415 | 1.50E-17 | #N/A | 54.8649 | 7.1396 | S | - |
| RSWS8N_07380 | WP_002720579.1 | -4.0563 | 2.77E-12 | yes | 7.371163 | 123.103 | T | pleD |
| RSWS8N_07460 | WP_002720595.1 | 2.372 | 6.11E-22 | #N/A | 68.7883 | 13.301 | V | - |
| RSWS8N_07465 | WP_002720596.1 | 3.8665 | 8.61E-16 | #N/A | 587.959 | 40.34 | K | - |
| RSWS8N_07560 | WP_002720615.1 | -2.7638 | 4.47E-30 | yes | 403.6652 | 2744.11 | L | recA |
| RSWS8N_07565 | WP_002720616.1 | -2.4604 | 1.30E-13 | yes | 67.4904 | 371.971 | - | - |
| RSWS8N_07570 | WP_082242059.1 | -4.2 | 4.50E-55 | yes | 21.40346 | 393.982 | T | cckA |
| RSWS8N_07600 | WP_002720623.1 | 2.214 | 6.25E-06 | #N/A | 383.411 | 82.733 | #N/D | #N/D |
| RSWS8N_07875 | WP_235697877.1 | -4.1305 | 8.92E-21 | yes | 11.14996 | 195.664 | T | - |
| RSWS8N_07940 | WP_002720689.1 | -2.812 | 4.38E-12 | #N/A | 13.193 | 93.042 | S | - |
| RSWS8N_08145 | WP_002720730.1 | -4.3581 | 8.20E-39 | yes | 10.4821 | 216.338 | #N/D | #N/D |
| RSWS8N_08425 | WP_002720786.1 | -4.028 | 3.70E-11 | #N/A | 0.17786 | 2.8179 | S | - |
| RSWS8N_08500 | WP_002720801.1 | 2.3952 | 5.40E-11 | #N/A | 127.308 | 24.217 | #N/D | #N/D |
| RSWS8N_08705 | WP_009561857.1 | 2.3632 | 4.12E-22 | #N/A | 176.901 | 34.429 | E | pchB |
| RSWS8N_08715 | WP_002720844.1 | 4.0505 | 1.30E-13 | #N/A | 3.47417 | 0.2105 | - | - |
| RSWS8N_08795 | WP_002720860.1 | 2.4631 | 1.56E-05 | yes | 47.00191 | 8.53712 | KLT | - |
| RSWS8N_09185 | WP_009561770.1 | 2.1627 | 0.0001054 | #N/A | 52.8723 | 11.831 | K | - |
| RSWS8N_09235 | WP_002720948.1 | -3.8649 | 2.63E-55 | yes | 62.89098 | 918.382 | K | mucS |
| RSWS8N_09490 | WP_002720999.1 | -4.755 | 1.54E-27 | yes | 16.82897 | 456.391 | T | - |
| RSWS8N_09525 | WP_002721006.1 | 2.1174 | 3.01E-12 | #N/A | 1546.75 | 356.8 | - | - |
| RSWS8N_09560 | WP_002721013.1 | 3.3103 | 5.10E-09 | #N/A | 55.6806 | 5.6128 | E | MA20_0 |
| RSWS8N_RS22460* | WP_235697905.1 | 2.7757 | 7.55E-07 | #N/A | 77.1823 | 11.28 | EGP | - |
| RSWS8N_09675 | WP_002721039.1 | 2.3154 | 4.06E-07 | #N/A | 110.693 | 22.263 | K | acnR |
| RSWS8N_09680 | WP_002721040.1 | 2.3837 | 3.73E-07 | #N/A | 249.42 | 47.845 | #N/D | #N/D |
| RSWS8N_09685 | WP_002721042.1 | 2.942 | 6.30E-07 | #N/A | 51.093 | 6.6561 | S | mbfA |
| RSWS8N_09735 | WP_002721053.1 | -2.6222 | 3.95E-05 | yes | 6.701492 | 41.4913 | S | - |

| RSWS8N_09740 | WP_002721054.1 | 2.1035 | 2.11E-10 | #N/A | 392.789 | 91.482 | - | - |
| --- | --- | --- | --- | --- | --- | --- | --- | --- |
| RSWS8N_09775 | WP_002721065.1 | 2.5858 | 8.40E-15 | #N/A | 22.6083 | 3.7739 | L | rnhA |
| RSWS8N_09785 | WP_002721067.1 | -2.8009 | 9.19E-15 | yes | 76.79164 | 536.421 | KT | osp |
| RSWS8N_10360 | WP_002721287.1 | 3.37374 | 6.27E-28 | yes | 1359.01 | 131.233 | #N/D | #N/D |
| RSWS8N_10495 | WP_002721325.1 | 2.3402 | 2.07E-06 | #N/A | 25.2559 | 4.9922 | J | rsmE |
| RSWS8N_10555 | WP_002721341.1 | 2.32192 | 4.08E-20 | yes | 42.71748 | 8.54671 | C | glcE |
| RSWS8N_10675 | WP_002721383.1 | 2.2173 | 6.65E-09 | #N/A | 101.985 | 21.948 | D | mipZ |
| RSWS8N_10780 | WP_002721427.1 | -5.5064 | 2.74E-81 | yes | 3.208667 | 144.293 | T | MA20_2 |
| RSWS8N_RS21485* | WP_009563136.1 | 2.3508 | 8.75E-26 | #N/A | 1618.17 | 317.48 | #N/D | #N/D |
| RSWS8N_10900 | WP_002721461.1 | 2.0022 | 4.75E-27 | #N/A | 253.942 | 63.444 | S | - |
| RSWS8N_10920 | WP_002721470.1 | -3.2709 | 2.50E-34 | yes | 4.314477 | 42.0323 | G | - |
| RSWS8N_10925 | WP_002721472.1 | -2.7841 | 2.51E-19 | yes | 31.93704 | 220.275 | S | - |
| RSWS8N_10930 | WP_002721473.1 | -2.7335 | 1.12E-29 | yes | 67.60428 | 450.13 | S | - |
| RSWS8N_11035 | WP_002721513.1 | -4.2028 | 5.60E-13 | yes | 0.974408 | 17.3519 | Q | - |
| RSWS8N_11040 | WP_002721514.1 | -5.2239 | 1.07E-41 | yes | 1.739389 | 63.13 | Q | - |
| RSWS8N_11045 | WP_002721515.1 | -6.89 | 3.03E-91 | yes | 0.264172 | 33.1529 | #N/D | #N/D |
| RSWS8N_11050 | WP_002721516.1 | -10.732 | 2.12E-41 | yes | 0.020533 | 38.3458 | H | wgeD |
| RSWS8N_11055 | WP_002721517.1 | -6.3234 | 4.69E-37 | yes | 0.467776 | 38.942 | OU | wgeF |
| RSWS8N_11060 | WP_002721518.1 | -5.5007 | 6.25E-22 | yes | 5.306996 | 241.637 | H | - |
| RSWS8N_11065 | WP_002721521.1 | -6.0736 | 2.61E-07 | yes | 1.502447 | 101.628 | V | aprD/wg |
| RSWS8N_11070 | WP_002721523.1 | -6.6799 | 5.06E-06 | yes | 0.663335 | 68.6269 | M | aprE |
| RSWS8N_11075 | WP_002721525.1 | -5.2527 | 7.29E-51 | yes | 30.66367 | 1172.23 | M | - |
| RSWS8N_11080 | WP_002721526.1 | -4.5256 | 1.56E-131 | yes | 11.62576 | 267.523 | #N/D | #N/D |
| RSWS8N_11085 | WP_002721528.1 | -4.7671 | 1.04E-143 | yes | 7.037707 | 191.976 | L | dnaE2 |
| RSWS8N_11090 | WP_002721530.1 | -2.6501 | 4.53E-34 | yes | 13.00068 | 81.4191 | M | mltB |
| RSWS8N_11170 | WP_002721563.1 | 3.2525 | 3.15E-16 | #N/A | 1303.29 | 136.9 | - | - |
| RSWS8N_11320 | WP_235697881.1 | -3.5049 | 5.09E-41 | yes | 5.670368 | 64.0162 | MP | - |
| RSWS8N_11325 | WP_002721631.1 | -3.7821 | 3.82E-20 | yes | 13.95339 | 192.923 | L | radC |
| RSWS8N_11440 | WP_002721670.1 | -2.8464 | 6.09E-05 | yes | 2.114987 | 15.4713 | S | comF |
| RSWS8N_11510 | WP_002721694.1 | -2.4333 | 2.95E-05 | yes | 1.362689 | 7.19175 | T | - |
| RSWS8N_11685 | WP_002721756.1 | 2.10064 | 1.17E-25 | yes | 90.31898 | 21.085 | G | - |
| RSWS8N_11690 | WP_002721758.1 | 2.17399 | 5.91E-12 | yes | 37.94805 | 8.42542 | S | - |
| RSWS8N_11710 | WP_002721767.1 | 3.94508 | 3.47E-29 | yes | 68.12362 | 4.41679 | C | pazS |
| RSWS8N_11720 | WP_002721771.1 | 2.0426 | 2.05E-08 | #N/A | 1445.44 | 351.21 | S | hvrA |
| RSWS8N_11755 | WP_002721783.1 | -2.024 | 0.0010576 | #N/A | 1.34172 | 5.4776 | I | - |
| RSWS8N_11990 | WP_002721864.1 | -4.7109 | 1.36E-46 | yes | 3.119961 | 83.051 | N | motB2 |
| RSWS8N_11995 | WP_002721867.1 | -5.6801 | 1.44E-105 | yes | 78.68085 | 4038.94 | N | flgE2 |
| RSWS8N_12000 | WP_002721869.1 | -6.5634 | 4.35E-91 | yes | 11.65463 | 1106.43 | N | flgK2 |
| RSWS8N_12005 | WP_002721871.1 | -6.5083 | 1.22E-294 | yes | 20.215 | 1845.38 | N | flgL2 |
| RSWS8N_12010 | WP_002721873.1 | -5.9678 | 1.21E-217 | yes | 13.43844 | 843.99 | N | flgI2 |
| RSWS8N_12015 | WP_002721875.1 | -2.7829 | 3.23E-42 | yes | 14.94633 | 103.024 | T | - |
| RSWS8N_12025 | WP_002721877.1 | -4.4618 | 9.02E-54 | yes | 10.13577 | 224.856 | N | fliP2 |
| RSWS8N_12030 | WP_002721878.1 | -4.7548 | 1.74E-66 | yes | 7.87073 | 209.385 | N | fliN2 |
| RSWS8N_12035 | WP_002721879.1 | -4.8402 | 5.07E-40 | yes | 9.050152 | 261.877 | N | fliH2 |
| RSWS8N_12040 | WP_037087267.1 | -4.563 | 1.08E-56 | yes | 29.3209 | 694.354 | N | fliF2 |
| RSWS8N_12045 | WP_002721881.1 | -4.2576 | 8.55E-19 | yes | 27.08426 | 519.912 | N | fliL2 |
| RSWS8N_12050 | WP_002721882.1 | -4.7941 | 6.03E-34 | yes | 31.84248 | 887.391 | - | - |
| RSWS8N_12055 | WP_002721883.1 | -4.7958 | 1.73E-48 | yes | 18.31196 | 511.568 | S | - |
| RSWS8N_12060 | WP_002721884.1 | -4.9106 | 1.89E-101 | yes | 17.79905 | 536.489 | N | motA2 |
| RSWS8N_12065 | WP_002721885.1 | -6.0882 | 1.37E-192 | yes | 7.036285 | 480.575 | O | - |
| RSWS8N_12070 | WP_011338874.1 | -4.9133 | 1.91E-35 | yes | 16.36952 | 495.728 | M | - |
| RSWS8N_12075 | WP_002721887.1 | -5.4064 | 3.51E-117 | yes | 6.973652 | 297.206 | N | flhA2 |
| RSWS8N_12080 | WP_002721888.1 | -5.2862 | 9.73E-36 | yes | 5.619879 | 221.624 | N | fliR2 |
| RSWS8N_12085 | WP_002721889.1 | -5.01 | 3.04E-49 | yes | 8.130193 | 263.758 | N | flhB2 |
| RSWS8N_12090 | WP_002721890.1 | -5.2571 | 3.63E-98 | yes | 6.010938 | 225.045 | - | - |
|  | WP_017140007.1 | -5.1238 | 4.76E-159 | yes | 9.697451 | 339.287 | N | fliL |
| RSWS8N_12095 | WP_002721891.1 | -5.2594 | 1.84E-56 | yes | 16.92296 | 651.332 | N | flgH2 |
| RSWS8N_12100 | WP_002721892.1 | -5.3402 | 2.83E-67 | yes | 17.56217 | 716.513 | N | flgA2 |

| RSWS8N_12105 | WP_002721893.1 | -4.8057 | 4.55E-107 | yes | 28.11192 | 786.895 | N | flgG2 |
| --- | --- | --- | --- | --- | --- | --- | --- | --- |
| RSWS8N_12110 | WP_002721894.1 | -4.6145 | 2.27E-47 | yes | 31.24549 | 767.568 | N | flgF2 |
| RSWS8N_12115 | WP_002721895.1 | -4.7823 | 1.75E-28 | yes | 49.725 | 1373.63 | N | fliQ2 |
| RSWS8N_12120 | WP_002721896.1 | -4.4144 | 9.79E-65 | yes | 62.76789 | 1343.67 | N | fliE2 |
| RSWS8N_12125 | WP_002721897.1 | -3.5363 | 5.53E-26 | yes | 74.34577 | 864.656 | N | flgC2 |
| RSWS8N_12130 | WP_002721898.1 | -3.5793 | 2.20E-20 | yes | 67.31331 | 806.539 | N | flgB2 |
| RSWS8N_12135 | WP_002721899.1 | -4.9202 | 1.21E-29 | yes | 4.957637 | 151.131 | NU | fliI2 |
| RSWS8N_12140 | WP_002721900.1 | -5.063 | 7.01E-19 | yes | 9.982866 | 337.404 | - | - |
| RSWS8N_12145 | WP_002721901.1 | -4.9933 | 0.00011202 | yes | 8.418463 | 269.322 | N | flgJ2 |
| RSWS8N_12155 | WP_002721903.1 | -5.421 | 5.95E-140 | #N/A | 32.4297 | 1398.2 | N | - |
| RSWS8N_12160 | WP_002721904.1 | -4.9906 | 1.92E-137 | yes | 37.97258 | 1209.79 | N | flgD2 |
| RSWS8N_12165 | WP_002721905.1 | -2.5708 | 1.32E-30 | yes | 36.57997 | 217.709 | H | ubiB |
| RSWS8N_12170 | WP_002721909.1 | 2.402 | 1.95E-22 | #N/A | 132.868 | 25.157 | H | ubiE |
| RSWS8N_12260 | WP_002721942.1 | -5.655 | 1.82E-71 | yes | 2.278198 | 115.498 | T | pleC |
| RSWS8N_12265 | WP_002721945.1 | -3.9027 | 8.88E-37 | yes | 1.655224 | 23.9515 | S | - |
| RSWS8N_12270 | WP_002721947.1 | -2.038 | 1.43E-15 | yes | 21.47125 | 88.2243 | L | recQ |
| RSWS8N_12310 | WP_002721957.1 | 2.47907 | 1.38E-30 | yes | 33.98036 | 6.09466 | H | pncB |
| RSWS8N_12315 | WP_002721958.1 | 2.7621 | 6.26E-23 | yes | 39.40056 | 5.79925 | Q | pncA |
| RSWS8N_12365 | WP_002721977.1 | -5.5555 | 5.79E-05 | yes | 1.446981 | 69.1607 | - | - |
| RSWS8N_12515 | WP_002722035.1 | -2.113 | 1.84E-08 | #N/A | 8.77817 | 38.368 | M | - |
| RSWS8N_12705 | WP_002722111.1 | -3.4937 | 1.53E-05 | yes | 7.304956 | 83.5939 | #N/D | #N/D |
| RSWS8N_12710 | WP_002722113.1 | -2.3179 | 3.17E-14 | yes | 2.079927 | 10.4358 | L | - |
| RSWS8N_12760 | WP_002722127.1 | 3.75863 | 3.25E-12 | yes | 1172.686 | 86.6909 | C | alkB2 |
| RSWS8N_12805 | WP_037087298.1 | 2.126 | 1.54E-14 | yes | 60.9069 | 13.968 | P | - |
| RSWS8N_12810 | WP_002722137.1 | 2.7651 | 3.90E-07 | #N/A | 59.248 | 8.6993 | #N/D | #N/D |
| RSWS8N_12815 | WP_002722141.1 | 2.1645 | 5.93E-06 | #N/A | 38.8169 | 8.6646 | P | - |
| RSWS8N_12825 | WP_002722145.1 | 2.0657 | 3.70E-17 | #N/A | 68.0722 | 16.271 | EP | oppB |
| RSWS8N_12830 | WP_002722147.1 | 3.0676 | 3.82E-45 | #N/A | 253.62 | 30.277 | E | oppA |
| RSWS8N_12930 | WP_002722176.1 | -4.5978 | 2.05E-31 | yes | 11.57711 | 280.964 | H | rsbW |
| RSWS8N_12935 | WP_002722179.1 | -4.4873 | 5.12E-37 | yes | 30.83566 | 694.298 | T | rsbV |
| RSWS8N_13025 | WP_002722219.1 | 2.2015 | 3.37E-21 | #N/A | 1603.01 | 348.83 | K | hvrA |
| RSWS8N_13045 | WP_002722234.1 | -4.3384 | 0.00013755 | yes | 5.841178 | 118.343 | T | divL |
| RSWS8N_13080 | WP_002722248.1 | -4.4577 | 5.22E-58 | yes | 7.581759 | 168.035 | - | - |
| RSWS8N_13100 | WP_002722256.1 | -2.388 | 0.0010994 | #N/A | 6.17554 | 32.725 | IQ | acpP |
| RSWS8N_13105 | WP_002722258.1 | -3.372 | 3.13E-40 | #N/A | 2.04031 | 21.267 | Q | - |
| RSWS8N_13110 | WP_002722260.1 | -2.287 | 0.0003347 | #N/A | 0.98557 | 4.7748 | S | - |
| RSWS8N_13225 | WP_002722292.1 | 2.99732 | 1.33E-32 | yes | 10821.33 | 1356.6 | H | pucB2 |
| RSWS8N_13230 | WP_002722294.1 | 2.31025 | 1.45E-23 | yes | 2446.526 | 493.762 | M | pucA2 |
| RSWS8N_13365 | WP_037087324.1 | -3.983 | 9.93E-46 | yes | 76.00024 | 1202.94 | NT | cheA2 |
| RSWS8N_13370 | WP_002722333.1 | -4.4883 | 2.45E-63 | yes | 92.71433 | 2083.13 | NT | cheW2 |
| RSWS8N_13375 | WP_002722335.1 | -3.8666 | 1.32E-31 | yes | 96.46267 | 1407.95 | NT | cheW3 |
| RSWS8N_13380 | WP_002722338.1 | -3.9918 | 1.45E-35 | yes | 95.27393 | 1516.62 | H | cheR2 |
| RSWS8N_13385 | WP_002722341.1 | -4.4255 | 1.69E-47 | yes | 36.98315 | 795.643 | NT | cheB1 |
| RSWS8N_13390 | WP_002722343.1 | -4.0321 | 9.09E-27 | yes | 50.29441 | 823.384 | NT | tlpC |
| RSWS8N_13395 | WP_037087337.1 | -3.092 | 5.15E-45 | #N/A | 25.6565 | 218.78 | O | yqjG |
| RSWS8N_13465 | WP_002722374.1 | 2.78759 | 3.45E-12 | yes | 50.37026 | 7.29894 | H | - |
| RSWS8N_13630 | WP_002722426.1 | -2.746 | 6.06E-20 | #N/A | 4.52965 | 30.511 | C | mcd |
| RSWS8N_14220 | WP_002722645.1 | -2.404 | 0.0057754 | #N/A | 0.59073 | 3.2784 | P | sodC |
| RSWS8N_14310 | WP_002722669.1 | -2.8069 | 7.45E-37 | yes | 8.651147 | 60.835 | H | rsmI |
| RSWS8N_14315 | WP_002722671.1 | -4.4924 | 6.63E-83 | yes | 13.49235 | 307.293 | L | - |
| RSWS8N_14325 | WP_002722674.1 | -3.2392 | 9.82E-24 | yes | 4.772706 | 45.4016 | O | comM |
| RSWS8N_14330 | WP_002722676.1 | 2.50057 | 1.87E-21 | yes | 535.0727 | 94.6507 | S | - |
| RSWS8N_14425 | WP_002722711.1 | -2.141 | 0.0004935 | #N/A | 2.3407 | 10.496 | S | - |
| RSWS8N_14555 | WP_002722757.1 | -2.996 | 1.78E-37 | yes | 5.121308 | 40.6872 | M | sleB |
| RSWS8N_14620 | WP_002722781.1 | -3.0831 | 1.71E-29 | yes | 6.671774 | 56.3453 | M | pulE/tfp |
| RSWS8N_14665 | WP_002722799.1 | -3.001 | 3.09E-23 | yes | 184.3752 | 1477.27 | E | potD |
| RSWS8N_14670 | WP_002722801.1 | -3.7679 | 1.50E-27 | yes | 90.60149 | 1235.12 | P | potB |
| RSWS8N_14675 | WP_002722803.1 | -3.6289 | 1.13E-28 | yes | 59.80503 | 740.334 | P | potC |

| RSWS8N_14710 | WP_002722818.1 | 5.1042 | 8.65E-17 | #N/A | 78.4146 | 2.2853 | K | - |
| --- | --- | --- | --- | --- | --- | --- | --- | --- |
| RSWS8N_14745 | WP_002722830.1 | -2.7535 | 5.97E-28 | yes | 13.96732 | 94.1088 | O | - |
| RSWS8N_14770 | WP_002722840.1 | -2.159 | 1.78E-09 | yes | 37.52399 | 167.596 | U | tadB |
| RSWS8N_14775 | WP_002722842.1 | -2.3504 | 5.12E-12 | yes | 40.27368 | 205.495 | U | cpaF |
| RSWS8N_14780 | WP_002722844.1 | -2.8166 | 1.99E-26 | yes | 14.59526 | 103.032 | U | cpaE/min |
| RSWS8N_14885 | WP_002722876.1 | -2.231 | 4.14E-09 | #N/A | 5.45306 | 25.247 | S | - |
| RSWS8N_14895 | WP_002722880.1 | -2.4769 | 1.06E-19 | yes | 7.474543 | 41.3305 | H | dusA |
|  | WP_082242126.1 | 2.8677 | 3.28E-11 | #N/A | 23.2779 | 3.1873 | K | - |
| RSWS8N_15314 | WP_002723078.1 | 4.3496 | 0.0012815 | #N/A | 60.4043 | 2.9663 | M | - |
| RSWS8N_15319 | WP_002723080.1 | 4.4958 | 5.94E-12 | #N/A | 44.5577 | 1.9773 | I | - |
| RSWS8N_15364 | WP_002723098.1 | 2.2776 | 4.20E-05 | #N/A | 6.38417 | 1.3211 | P | - |
| RSWS8N_15379 | WP_002723104.1 | -3.1015 | 1.04E-30 | yes | 6.241559 | 53.7785 | C | stcD |
|  | WP_082242128.1 | -4.23 | 7.33E-55 | #N/A | 6.318 | 121.07 | #N/D | #N/D |
| RSWS8N_15389 | WP_002723108.1 | -4.1084 | 2.97E-13 | yes | 2.889107 | 50.4896 | V | - |
| RSWS8N_15394 | WP_011339530.1 | 5.4349 | 4.15E-39 | #N/A | 46.3245 | 1.0701 | K | - |
|  | WP_082242129.1 | 2.2238 | 1.19E-06 | #N/A | 65.1406 | 13.977 | #N/D | #N/D |
| RSWS8N_15564 | WP_002723164.1 | -2.622 | 1.80E-06 | #N/A | 0.52271 | 3.1965 | E | - |
| RSWS8N_15764 | WP_002723238.1 | -3.034 | 0.0007778 | #N/A | 0.33989 | 3.0176 | S | - |
| RSWS8N_RS22515* | WP_235697917.1 | -3.154 | 8.31E-08 | #N/A | 1.78678 | 16.425 | L | rep |
| RSWS8N_16384 | WP_002723456.1 | 2.416 | 0.0013957 | #N/A | 5.0099 | 0.9446 | K | - |
| RSWS8N_16454 | WP_002723486.1 | 3.5671 | 4.76E-29 | #N/A | 108.792 | 9.1878 | T | dorS |
| RSWS8N_16459 | WP_002723488.1 | 2.4612 | 5.48E-25 | #N/A | 26.5584 | 4.8347 | K | torR |
| RSWS8N_16464 | WP_002723490.1 | 5.6613 | 6.69E-24 | #N/A | 414.059 | 8.1888 | C | - |
| RSWS8N_16469 | WP_002723492.1 | 5.8863 | 8.57E-08 | #N/A | 133.093 | 2.2515 | S | - |
| RSWS8N_16479 | WP_002723495.1 | 5.5414 | 0.0021085 | #N/A | 12.7462 | 0.2739 | H | moeA |
| RSWS8N_16569 | WP_002723525.1 | -2.258 | 0.0001636 | #N/A | 1.39529 | 6.7761 | - | - |
| RSWS8N_16574 | WP_002723528.1 | -2.485 | 0.0012637 | #N/A | 0.41895 | 2.4614 | S | - |
| RSWS8N_16599 | WP_002723538.1 | -4.01 | 5.17E-24 | #N/A | 4.17318 | 67.318 | S | - |
| RSWS8N_16654 | WP_002723558.1 | -2.435 | 2.63E-19 | #N/A | 3.08425 | 16.838 | NT | mcrA |
| RSWS8N_16684 | WP_002723567.1 | 2.5486 | 1.41E-22 | #N/A | 148.902 | 25.454 | P | phnA |
| RSWS8N_16769 | WP_002723595.1 | -5.1781 | 2.95E-95 | yes | 5.941074 | 215.802 | P | - |
| RSWS8N_16774 | WP_002723596.1 | -5.6004 | 5.44E-65 | yes | 21.54171 | 1054.91 | S | - |
| RSWS8N_16784 | WP_002723600.1 | -6.1432 | 3.39E-158 | yes | 23.49908 | 1664.62 | NT | mcpR |
| RSWS8N_16914 | WP_002723642.1 | -2.159 | 1.10E-10 | #N/A | 13.6398 | 60.768 | T | - |
| RSWS8N_16919 | WP_002723644.1 | -3.224 | 5.09E-19 | #N/A | 1.86937 | 17.726 | KLT | - |
| RSWS8N_16924 | WP_037087852.1 | -2.907 | 5.12E-25 | #N/A | 2.0277 | 15.423 | M | - |
| RSWS8N_16929 | WP_002723649.1 | -2.854 | 3.03E-18 | #N/A | 1.58515 | 11.54 | V | - |
| RSWS8N_16934 | WP_002723651.1 | -2.581 | 1.08E-09 | #N/A | 2.00685 | 12.156 | V | - |
| RSWS8N_16939 | WP_002723652.1 | -3.23 | 4.72E-16 | #N/A | 3.21483 | 30.178 | S | - |
| RSWS8N_16944 | WP_002723655.1 | -2.476 | 0.0013613 | #N/A | 8.41016 | 46.715 | #N/D | #N/D |
| RSWS8N_16949 | WP_002723657.1 | -2.654 | 4.91E-06 | #N/A | 3.20518 | 20.147 | KLT | - |
| RSWS8N_16954 | WP_002723660.1 | -2.308 | 1.75E-11 | #N/A | 8.88011 | 44.005 | S | - |
| RSWS8N_16959 | WP_002723661.1 | -5.1409 | 1.39E-96 | yes | 6.940574 | 244.454 | Q | - |
| RSWS8N_16969 | WP_002723664.1 | -2.6685 | 0.00975228 | yes | 0.180048 | 1.16997 | G | - |
| RSWS8N_17019 | WP_002723680.1 | -3.904 | 0.0060445 | #N/A | 0.14034 | 2.0412 | C | - |
| RSWS8N_17349 | WP_037087877.1 | -5.4061 | 7.74E-60 | yes | 10.1935 | 434.55 | #N/D | #N/D |
| RSWS8N_17719 | WP_002723944.1 | 2.158 | 1.70E-06 | #N/A | 240.206 | 53.878 | E | MA20_2 |
| RSWS8N_17729 | WP_037087907.1 | -2.205 | 2.77E-12 | #N/A | 2.38307 | 11.086 | S | - |
| RSWS8N_17734 | WP_002723950.1 | -3.558 | 6.08E-17 | #N/A | 3.84926 | 45.834 | S | - |
| RSWS8N_17739 | WP_002723952.1 | -2.07 | 0.0003752 | #N/A | 1.24515 | 5.2385 | S | - |
| RSWS8N_17774 | WP_002723961.1 | -2.616 | 8.56E-27 | #N/A | 158.522 | 973.35 | - | - |
| RSWS8N_17779 | WP_037088030.1 | -2.477 | 1.07E-26 | #N/A | 337.403 | 1880.1 | #N/D | #N/D |
| RSWS8N_17824 | WP_002723978.1 | 2.7237 | 1.28E-16 | #N/A | 96.5638 | 14.631 | KT | - |
| RSWS8N_17844 | WP_002723982.1 | 2.4464 | 8.25E-32 | #N/A | 216.47 | 39.746 | M | - |
| RSWS8N_17974 | WP_002724023.1 | -7.188 | 3.54E-05 | #N/A | 0 | 2.1026 | #N/D | #N/D |
| RSWS8N_18019 | WP_002724032.1 | -3.163 | 0.0003777 | #N/A | 2.79098 | 25.603 | - | - |
| RSWS8N_18024 | WP_002724033.1 | -3.018 | 0.0010576 | #N/A | 1.60949 | 13.303 | K | - |

| RSWS8N_18044 | WP_002724037.1 | -3.2008 | 6.12E-10 | yes | 0.297033 | 2.82721 | L | - |
| --- | --- | --- | --- | --- | --- | --- | --- | --- |
| RSWS8N_18049 | WP_002724038.1 | -4.644 | 3.33E-10 | #N/A | 0.47886 | 12.582 | S | - |
| RSWS8N_18054 | WP_235697909.1 | -4.8 | 9.81E-10 | #N/A | 0.26612 | 8.3497 | - | - |
| RSWS8N_18059 | WP_002724042.1 | -3.967 | 2.99E-06 | #N/A | 0.4306 | 7.1646 | - | - |
| RSWS8N_18064 | WP_002724044.1 | -4.889 | 4.25E-07 | #N/A | 0.25126 | 7.8029 | #N/D | #N/D |
| RSWS8N_18069 | WP_002724046.1 | -3.648 | 1.90E-05 | #N/A | 1.09075 | 14.885 | #N/D | #N/D |
| RSWS8N_18074 | WP_002724047.1 | -3.33 | 2.45E-06 | yes | 1.26168 | 12.3271 | S | - |
| RSWS8N_18079 | WP_002724049.1 | -5.064 | 1.45E-10 | #N/A | 0.43551 | 17.23 | #N/D | #N/D |
| RSWS8N_18084 | WP_002724051.1 | -2.6964 | 0.00029535 | yes | 2.413804 | 15.1007 | #N/D | #N/D |
| RSWS8N_18094 | WP_002724054.1 | -3.0404 | 0.0082009 | yes | 1.071269 | 8.41716 | S | - |
| RSWS8N_18109 | WP_002724060.1 | -4.45 | 8.64E-51 | #N/A | 1.80466 | 38.956 | V | - |
| RSWS8N_18114 | WP_002724062.1 | -4.366 | 7.11E-58 | #N/A | 5.89643 | 122.07 | - | - |
| RSWS8N_18119 | WP_002724064.1 | -4.327 | 2.27E-36 | #N/A | 4.53611 | 92.811 | S | - |
| RSWS8N_18124 | WP_002724066.1 | -3.357 | 7.89E-22 | #N/A | 4.87589 | 51.012 | K | - |
| RSWS8N_18129 | WP_002724067.1 | -3.66 | 3.74E-20 | #N/A | 1.69578 | 21.133 | S | - |
| RSWS8N_18134 | WP_037087927.1 | -4.479 | 1.70E-19 | #N/A | 0.43191 | 10.008 | S | - |
| RSWS8N_18139 | WP_002724071.1 | -2.915 | 0.0002862 | #N/A | 0.43356 | 3.116 | S | - |
| RSWS8N_18144 | WP_002724073.1 | -3.767 | 2.61E-17 | #N/A | 0.39925 | 5.124 | S | - |
| RSWS8N_18149 | WP_002724075.1 | -2.795 | 3.29E-06 | #N/A | 0.47046 | 3.3169 | L | - |
| RSWS8N_18154 | WP_002724077.1 | -4.505 | 9.76E-82 | #N/A | 4.43016 | 100.34 | S | - |
| RSWS8N_18159 | WP_002724078.1 | -4.931 | 6.97E-28 | #N/A | 3.55467 | 109.85 | S | - |
| RSWS8N_18164 | WP_037087931.1 | -6.852 | 1.25E-35 | #N/A | 0.55179 | 64.702 | #N/D | #N/D |
| RSWS8N_18169 | WP_166486352.1 | -5.158 | 2.26E-29 | #N/A | 1.69775 | 65.457 | #N/D | #N/D |
| RSWS8N_18174 | WP_002724084.1 | -4.041 | 3.05E-32 | #N/A | 1.72691 | 28.649 | S | - |
| RSWS8N_18179 | WP_002724085.1 | -3.715 | 1.94E-21 | #N/A | 1.26993 | 16.289 | S | - |
| RSWS8N_18184 | WP_002724087.1 | -7.183 | 1.50E-15 | #N/A | 0.05231 | 7.1135 | - | - |
| RSWS8N_18189 | WP_002724089.1 | -3.4398 | 0.00011241 | yes | 1.342572 | 14.153 | S | - |
| RSWS8N_18204 | WP_002724096.1 | -3.768 | 2.40E-09 | #N/A | 0.29429 | 4.0602 | S | J |
| RSWS8N_18209 | WP_002724098.1 | -4.968 | 1.26E-08 | #N/A | 0.19258 | 6.9156 | S | - |
| RSWS8N_18214 | WP_002724100.1 | -5.135 | 4.83E-21 | #N/A | 0.5836 | 21.316 | S | - |
| RSWS8N_18219 | WP_002724103.1 | -5.578 | 7.54E-15 | #N/A | 0.17828 | 7.0116 | - | - |
| RSWS8N_18224 | WP_002724105.1 | -4.343 | 1.86E-12 | #N/A | 2.91041 | 59.645 | S | - |
| RSWS8N_18229 | WP_002724107.1 | -4.036 | 1.28E-16 | #N/A | 6.10809 | 101.58 | S | gpFII |
| RSWS8N_18234 | WP_002724109.1 | -3.665 | 1.18E-25 | #N/A | 2.45195 | 30.656 | S | - |
| RSWS8N_18239 | WP_002724111.1 | -3.56 | 2.27E-12 | #N/A | 2.43931 | 27.723 | #N/D | #N/D |
| RSWS8N_18244 | WP_002724113.1 | -3.181 | 7.16E-14 | #N/A | 0.4523 | 4.0896 | S | - |
| RSWS8N_18249 | WP_002724115.1 | -6.679 | 2.01E-05 | #N/A | 0.01166 | 2.0563 | S | gpU1 |
| RSWS8N_18284 | WP_002724120.1 | -3.806 | 6.81E-18 | #N/A | 2.46568 | 33.835 | H | dam |
| RSWS8N_18574 | WP_002724213.1 | -4.9163 | 3.23E-56 | yes | 24.13492 | 730.311 | T | mcpV |
| RSWS8N_RS22555* | WP_082242159.1 | -5.032 | 3.13E-62 | #N/A | 30.4814 | 1001 | #N/D | #N/D |
| RSWS8N_18584 | WP_002724217.1 | -5.333 | 1.88E-73 | #N/A | 10.6937 | 439.95 | - | - |
| RSWS8N_18589 | WP_002724219.1 | -5.3315 | 6.77E-31 | yes | 6.112292 | 248.364 | L | xth |
| RSWS8N_18594 | WP_002724221.1 | -2.6311 | 9.08E-21 | yes | 4.942029 | 30.9291 | IQ | csgA |
| RSWS8N_18609 | WP_002724226.1 | -2.14 | 3.25E-05 | #N/A | 7.34042 | 32.455 | S | - |
| RSWS8N_18619 | WP_002724230.1 | -2.339 | 0.0007798 | #N/A | 0.34651 | 1.7015 | S | - |
| RSWS8N_18644 | WP_002724241.1 | 2.4158 | 0.0003392 | #N/A | 11.3938 | 2.145 | #N/D | #N/D |
| RSWS8N_18979 | WP_002724349.1 | 2.03672 | 1.22E-09 | yes | 52.42344 | 12.7797 | G | - |
| RSWS8N_18994 | WP_002724352.1 | 2.0328 | 1.61E-06 | #N/A | 2.46009 | 0.6039 | G | - |
| RSWS8N_19014 | WP_002724359.1 | 3.7289 | 5.09E-60 | #N/A | 26.9741 | 2.0365 | C | MA20_0 |
| RSWS8N_19364 | WP_002724495.1 | 2.0627 | 3.74E-09 | #N/A | 19.218 | 4.6073 | C | dmgdh2 |
| RSWS8N_19394 | WP_002724507.1 | -4.0864 | 7.90E-63 | yes | 48.9222 | 835.923 | S | gvpA1 |
| RSWS8N_19399 | WP_002724509.1 | -4.3273 | 1.01E-34 | yes | 14.88785 | 299.713 | F | hutH |
| RSWS8N_19404 | WP_002724511.1 | -5.5356 | 5.13E-56 | yes | 14.16343 | 651.785 | S | gvpK |
| RSWS8N_19409 | WP_002724512.1 | -5.792 | 4.49E-50 | yes | 8.039109 | 446.626 | #N/D | gvpL/F |
| RSWS8N_19414 | WP_002724513.1 | -6.1674 | 4.96E-28 | yes | 9.826217 | 717.279 | S | gvpS |
| RSWS8N_19419 | WP_002724514.1 | -6.5484 | 4.85E-47 | yes | 9.460239 | 891.486 | S | gvpL/F |
| RSWS8N_19424 | WP_002724515.1 | -6.2015 | 3.41E-117 | yes | 13.14384 | 972.751 | O | gvpL/F |

| RSWS8N_19429 | WP_002724516.1 | -5.8087 | 3.44E-110 | yes | 35.69001 | 1992.75 | S | gvpG |
| --- | --- | --- | --- | --- | --- | --- | --- | --- |
| RSWS8N_19434 | WP_002724517.1 | -5.8927 | 4.93E-129 | yes | 12.29521 | 729 | J | gvpL/F |
| RSWS8N_19439 | WP_002724518.1 | -5.7095 | 1.27E-93 | yes | 40.93124 | 2149 | O | gvpH |
| RSWS8N_19444 | WP_002724519.1 | -5.5597 | 8.70E-67 | yes | 46.45205 | 2200.6 | S | gvpA2 |
| RSWS8N_19449 | WP_011339430.1 | -6.1725 | 2.95E-103 | yes | 24.11721 | 1753.15 | S | gvpO |
| RSWS8N_RS21870* | WP_081597915.1 | -5.692 | 4.72E-96 | #N/A | 20.1309 | 1053 | #N/D | #N/D |
| RSWS8N_19459 | WP_082242171.1 | -4.7675 | 3.01E-41 | yes | 43.09839 | 1176 | S | gvpN |
| RSWS8N_19519 | WP_002724556.1 | -2.007 | 1.70E-05 | #N/A | 73.2935 | 295.06 | M | - |
| RSWS8N_19709 | WP_002724636.1 | -2.7869 | 1.57E-13 | yes | 1.840649 | 12.7964 | #N/D | #N/D |
| RSWS8N_19719 | WP_002724638.1 | -5.1221 | 4.94E-51 | yes | 9.959062 | 350.139 | S | - |
| RSWS8N_19724 | WP_002724640.1 | -5.4937 | 1.18E-69 | yes | 2.67181 | 118.462 | M | - |
| RSWS8N_19729 | WP_002724642.1 | -4.8379 | 2.33E-96 | yes | 3.660327 | 104.84 | V | - |
| RSWS8N_19734 | WP_002724645.1 | -5.3227 | 8.62E-132 | yes | 4.264884 | 171.817 | O | - |
| RSWS8N_19739 | WP_002724648.1 | -6.565 | 6.69E-71 | #N/A | 6.78437 | 643.47 | #N/D | #N/D |
| RSWS8N_19744 | WP_002724649.1 | -5.1979 | 1.52E-106 | yes | 0.921718 | 33.3761 | Q | - |
| RSWS8N_19804 | WP_002724672.1 | 2.8613 | 0.00101041 | yes | 6.836253 | 0.95104 | S | - |
| RSWS8N_RS19635* |  | 2.0777 | 0.0016789 | #N/A | 2.10783 | 0.4945 | #N/D | #N/D |
| RSWS8N_19864 | WP_002724684.1 | -6.4705 | 1.85E-128 | yes | 47.90039 | 4255.85 | N | flaA |
| RSWS8N_19869 | WP_012642306.1 | -6.59 | 4.11E-131 | yes | 13.93183 | 1349.07 | N | flaF |
| RSWS8N_19874 | WP_002724687.1 | -6.3949 | 4.18E-234 | yes | 9.136171 | 769.791 | N | flbT |
| RSWS8N_19879 | WP_002724689.1 | -6.7425 | 1.14E-92 | yes | 3.278629 | 358.521 | S | - |
| RSWS8N_20139 | WP_002724790.1 | -2.8362 | 0.00037226 | yes | 0.400024 | 2.70251 | S | - |
| RSWS8N_20234 | WP_002724827.1 | 2.94817 | 8.60E-05 | yes | 5.621438 | 0.72625 | C | - |
| RSWS8N_20404 | WP_002724880.1 | -2.875 | 1.22E-07 | #N/A | 0.38149 | 2.8212 | EP | - |
| RSWS8N_20409 | WP_002724882.1 | -2.261 | 0.0001398 | #N/A | 1.21926 | 5.8896 | F | - |
| RSWS8N_20524 | WP_002724921.1 | -2.711 | 8.91E-15 | yes | 5.391843 | 35.6502 | L | - |
| RSWS8N_20584 | WP_002724945.1 | -3.3426 | 1.23E-10 | yes | 4.085498 | 42.3365 | #N/D | mcp |
| RSWS8N_20589 | WP_002724946.1 | -3.1754 | 7.57E-13 | yes | 10.29864 | 93.8106 | T | - |
| RSWS8N_20674 | WP_002724964.1 | 2.4739 | 4.42E-39 | #N/A | 325.608 | 58.659 | T | - |
| RSWS8N_20679 | WP_002724966.1 | 2.8646 | 1.32E-38 | #N/A | 240.551 | 33.058 | K | - |
| RSWS8N_20744 | WP_002724990.1 | 3.02086 | 0.00035301 | yes | 9.398547 | 1.15762 | - | - |
| RSWS8N_20904 | WP_002725022.1 | -5.264 | 1.04E-06 | #N/A | 0.03706 | 1.7228 | P | glnP |
| RSWS8N_21104 | WP_002725087.1 | -2.381 | 0.0011328 | #N/A | 0.27592 | 1.5198 | C | - |
| RSWS8N_21139 | WP_082242204.1 | -3.4748 | 1.72E-16 | yes | 2.226491 | 25.0966 | M | - |
| RSWS8N_21144 | WP_002725103.1 | -3.0461 | 2.82E-23 | yes | 5.916975 | 48.5649 | - | - |
| RSWS8N_21149 | WP_002725106.1 | -3.2974 | 1.09E-37 | yes | 2.482765 | 24.48 | M | - |
| RSWS8N_21154 | WP_002725108.1 | -3.4 | 2.48E-45 | #N/A | 6.04037 | 64.465 | #N/D | #N/D |
| RSWS8N_21159 | WP_011331295.1 | -4.842 | 2.79E-54 | #N/A | 11.5208 | 332.31 | M | - |
| RSWS8N_21189 | WP_002725122.1 | -6.865 | 1.26E-05 | #N/A | 0.04547 | 2.9211 | - | - |

* new_ID

**genes part of the CtrA regulon (log_2_FC ≥ 1) are highlighted
