# Supplementary material for: CerM and Its Antagonist CerN Are New Components of the Quorum Sensing System in Cereibacter sphaeroides, Signaling to the CckA/ChpT/CtrA System
Source: Microbiologyopen. 2024 Dec 18;13(6):e012. doi: 10.1002/mbo3.70012 (PMC11655674; doi:10.1002/mbo3.70012)
Supplement: Supplementary file 16 — Supporting information. [file MBO3-13-e012-s013.docx]

**Table_A4_R. Transcription factors controlled by CerM/CerN.**

|  |  |  |  | **FPKM** | |  |  |
| --- | --- | --- | --- | --- | --- | --- | --- |
| **old_ID** | **prot_ID** | **log2FC** | **padj** | ***ΔcerN*/**  **pcerM** | **AM1/**  **pRK** | **Name** | **Description** |
| RSWS8N_03320 | WP_002719770.1 | -3.664 | 1.19E-19 | 170.29 | 2160.84 | ctrA | Two component transcriptional regulator, winged helix family |
| RSWS8N_09675 | WP_002721039.1 | 2.3154 | 4.06E-07 | 110.69 | 22.26 | acnR | AefR-like transcriptional repressor, C-terminal region |
| RSWS8N_14710 | WP_002722818.1 | 5.1042 | 8.65E-17 | 78.41 | 2.28 | - | Transcriptional regulator |
|  | WP_082242126.1 | 2.8677 | 3.28E-11 | 23.27 | 3.18 | - | LuxR transcriptional regulator |
| RSWS8N_15394 | WP_011339530.1 | 5.4349 | 4.15E-39 | 46.32 | 1.07 | - | LuxR transcriptional regulator |
| RSWS8N_16384 | WP_002723456.1 | 2.416 | 0.0013 | 5.01 | 0.94 | - | Transcriptional regulator, gntR family |
| RSWS8N_16459 | WP_002723488.1 | 2.4612 | 5.48E-25 | 26.55 | 4.83 | torR | Transcriptional regulatory protein, C terminal |
| RSWS8N_17824 | WP_002723978.1 | 2.7237 | 1.28E-16 | 96.56 | 14.63 | - | Transcriptional regulator with a LuxR-type HTH domain |
| RSWS8N_20679 | WP_002724966.1 | 2.8646 | 1.32E-38 | 240.55 | 33.05 | - | Transcriptional regulator |
| RSWS8N_18024 | WP_002724033.1 | -3.018 | 0.00105 | 1.60 | 13.30 | - | Prophage anti-repressor |
