# Supplementary material for: CerM and Its Antagonist CerN Are New Components of the Quorum Sensing System in Cereibacter sphaeroides, Signaling to the CckA/ChpT/CtrA System
Source: Microbiologyopen. 2024 Dec 18;13(6):e012. doi: 10.1002/mbo3.70012 (PMC11655674; doi:10.1002/mbo3.70012)
Supplement: Supplementary file 17 — Supporting information. [file MBO3-13-e012-s007.docx]

**Table_A5_R. Prediction of the MBS using the position weight matrix describing the GtaR binding site.**

| **RegRegion** | **Old_ID*** | **start** | **end** | **score** | **strand** | **sequence** | **log2FC** |
| --- | --- | --- | --- | --- | --- | --- | --- |
| 1 | RSWS8N_14710 | 170 | 191 | 14.3 | + | GACATATCCAAAAAGACAGGTT | 5.104 |
|  | RSWS8N_14710 | 170 | 191 | 14.2 | - | AACCTGTCTTTTTGGATATGTC | 5.104 |
|  | RSWS8N_14710 | 156 | 177 | 10.7 | - | GATATGTCCATTCATACAAGCT | 5.104 |
|  | RSWS8N_14710 | 156 | 177 | 10.6 | + | AGCTTGTATGAATGGACATATC | 5.104 |
|  | RSWS8N_14710 | 186 | 207 | 8 | - | AACTTGTCTTGAAACAAACCTG | 5.104 |
|  | RSWS8N_14710 | 186 | 207 | 7.5 | + | CAGGTTTGTTTCAAGACAAGTT | 5.104 |
| 2 | RSWS8N_17824 | 129 | 150 | 11.4 | + | ACCGGGTCTTTTTAGACAGGTC | 2.724 |
|  | RSWS8N_17824 | 129 | 150 | 11.8 | - | GACCTGTCTAAAAAGACCCGGT | 2.724 |
| 3 | RSWS8N_07465 | 197 | 218 | 8.8 | + | GGCCTGTCGGTTTGGGCAGGTC | 3.866 |
|  | RSWS8N_07465 | 197 | 218 | 8.6 | - | GACCTGCCCAAACCGACAGGCC | 3.866 |
| 4 | RSWS8N_06370 | 18 | 39 | 8.3 | - | GACATGACGGTCTCGACCGGTC | -3.319 |
|  | RSWS8N_06370 | 18 | 39 | 8.2 | + | GACCGGTCGAGACCGTCATGTC | -3.319 |
|  | RSWS8N_06370 | 51 | 72 | 8.1 | - | TATGTGTCCACTTAACCTTCTT | -3.319 |
|  | RSWS8N_06370 | 51 | 72 | 8 | + | AAGAAGGTTAAGTGGACACATA | -3.319 |
| 5 | RSWS8N_09740 | 157 | 178 | 7.8 | + | TATACCTCACAAAGGTTAGCTT | 2.104 |
|  | RSWS8N_09740 | 157 | 178 | 7.8 | - | AAGCTAACCTTTGTGAGGTATA | 2.104 |
| 6 | RSWS8N_15394 | 175 | 196 | 7.7 | - | CATCTGGTCAAAAAAATAAGTT | 5.435 |
|  | RSWS8N_15394 | 163 | 184 | 7.6 | - | AAAATAAGTTATTACATGGGCT | 5.435 |
|  | RSWS8N_15394 | 163 | 184 | 7.3 | + | AGCCCATGTAATAACTTATTTT | 5.435 |
| 7 | RSWS8N_18019 | 240 | 261 | 7.6 | + | TACCTATCCTTTAACCGTAATC | -3.163 |
|  | RSWS8N_18019 | 240 | 261 | 7.5 | - | GATTACGGTTAAAGGATAGGTA | -3.163 |
| 8 | RSWS8N_21154 | 106 | 127 | 7.1 | - | AACATGTGATGAAAGTTCTGGT | -3.4 |
|  | RSWS8N_21159 | 215 | 236 | 7.1 | + | AACATGTGATGAAAGTTCTGGT | -3.4 |
| 9 | RSWS8N_02995 | 22 | 43 | 6.9 | - | TCTACGGCCTATAGGACCGATC | 2.594 |
| INCLUDED | RSWS8N_13935 | 127 | 148 | 5.1 | + | CACCATTGCCAAAAAACGTGTT | -1.582 |

*Regulatory regions tested by EMSA are highlighted.
