# Supplementary material for: CerM and Its Antagonist CerN Are New Components of the Quorum Sensing System in Cereibacter sphaeroides, Signaling to the CckA/ChpT/CtrA System
Source: Microbiologyopen. 2024 Dec 18;13(6):e012. doi: 10.1002/mbo3.70012 (PMC11655674; doi:10.1002/mbo3.70012)
Supplement: Supplementary file 18 — Supporting information. [file MBO3-13-e012-s012.docx]

**Figure Legends of Supplementary Figures**

**Figure_A1.** Role of the LuxR homologues on the swimming behavior of strain JV18 (Δ*cerN*::Hyg). At the left side of the figure, a soft-agar plate containing Sistrom’s minimal medium supplemented with 0.2% casamino acids was inoculated with the indicated strains and incubated for 60 h under photoheterotrophic conditions. Δ*cerN*, (JV18, Δ*cerN*::Hyg), Δ*cerN* Δ*cerM* (JV20, Δ*cerN*::Hyg Δ*cerM*), Δ*cerN* Δ*gtaR* (JV29, Δ*cerN*::*aadA* Δ*gtaR*::*uidA*-*aadA*), Δ*cerN* Δ1539 (JV31, Δ*cerN*::*aadA* Δ15394::*uidA*-Hyg) and Δ*cerN* ΔRS15160 (JV30, Δ*cerN*::*aadA* ΔRS15160::*uidA*-Hyg). At the right side of the figure, a soft agar plate of the complemented strain Δ*cerN* Δ*cerM* (JV20, Δ*cerN*::Hyg Δ*cerM*) with the plasmid pcerM. AM1, Δ*cerN* (JV18, Δ*cerN*::Hyg) and Δ*cerN* Δ*cerM* (JV20, Δ*cerN*::Hyg Δ*cerM*) carrying the empty vector pRK415 (labeled as pRK) were included.

**Figure_A2.** Ribbon view of the dimeric CerM structure predicted by AlphaFold 2. The autoinducer-binding domain (autoind-bind) and DNA binding domain (HTH) are indicated. The glutamine residue 189 (Q189), which is mutated in strain SupCerI, is shown in blue.

**Figure_A3.** Evaluation of the role of CerR and *orf-2* on the swimming behavior of AM1. A, Schematic representation of the deleted region in strain JV25 (Δ*cerOp*::*aadA*). A set of plasmids was obtained in which different versions of the *cer* operon were cloned into the pRK415 plasmid. The three constructions used in this work are represented below the operon architecture. The DNA fragment included in each plasmid is shown in blue, while the region that was deleted is shown in red. The name of each plasmid is indicted on the left. The swimming ability of the Δ*cerOp* strain (JV25) carrying each of these plasmids was tested on soft-agar plates. Below, a boxplot representing the distribution of the swimming ring diameters from triplicates of at least three independent experiments is shown. The data for the parental strain AM1 are shown in a red box. B, Detection of CtrA in total cell extracts of the indicated mutant strains by immunoblotting, using α-CtrA antibodies. Δ*ctrA*, is strain EA1 (*ΔctrA*::*aadA*), Δ*cerI*, JV14 (Δ*cerI*::*aadA*), Δ*cerR*, JV16 (Δ*cerR*::*aadA*), Δ*cerOp*, JV25 (Δ*cerOp*::*aadA*) Controls corresponding to AM1 and Δ*ctrA* samples are on the left, while samples from Δ*cerOp* strains carrying the indicated plasmid are shown on the right (labeled by a pink shape below). C, RT-PCR assay using total RNA from AM1 cells and the oligonucleotides DOWNcerRfw and asRNAcerI, which are complementary to *cerR* and *cerI*, respectively. These amplify a short region of these genes and the intercistronic region between them. Lane 1 shows the RT-PCR reaction corresponding to an internal region of *ctrA*; lanes 2 and 3 show the RT-PCR reaction corresponding to the *cerR*-*cerI* region without reverse transcriptase or with reverse transcriptase, respectively. On the right, the MW lane corresponds to HaeIII-PhiX174 DNA size standard.

**Figure_A4.** Functional complementation of the non-motile phenotype of strain Δ*cerI* [JV14 (Δ*cerI*::*aadA*)] by the quorum sensing mutants Δ*cerN* and Δ*cerM*. Soft-agar plates were inoculated with the strains AM1 (positive control), Δ*cerN* [JV17 (Δ*cerN*::*aadA*)] and Δ*cerM* [JV19 (Δ*cerM*::*aadA*)] at the center of the plate. The inoculation point of these strains is indicated by an arrow. The non-motile strain Δ*cerI* (asterisks) was inoculated near and farther of the central inoculum. After 60 h of incubation, swimming of Δ*cerI* was evident for the cells grown near AM1, Δ*cerN* and Δ*cerN*.

**Figure_A5.** Immunodetection of CerM in Δ*cerI* and Δ*cerN* strains. Total cell extracts of the indicated mutant were tested by immunoblotting using an α-CerM antibody. JV18 (Δ*cerM*::*aadA*), JV14 (Δ*cerI*::*aadA*), JV17 (Δ*cerN*::*aadA*). CerM antiserum detects several nonspecific polypeptides on the blot, positive identification of CerM was done by including a cell extract of Δ*cerM*; in this sample, a single band of the expected size for CerM (22.4 kDa) was missing as compared with AM1. Furthermore, the signal was recovered by the introduction of pcerM. The signal assigned as CerM is indicated by an arrow.

**Figure_A6.** β-galactosidase activities promoted by AHL in *A. tumefaciens* NTL4 (pZLR4) strain. Cultures of the reporter strain were grown in the presence of different concentrations of 3-OH-C14-HSL (purple bars) or by adding 2 μl of extracts from the cell cultures supernatants of strains AM1 or Δ*cerI* (JV14) (black bars). Three independent assays were performed, and the average value is shown. The standard deviation is included.

**Figure_A7.** Electrophoretic mobility shift of the regulatory regions of *ctrA*, *cckA* and *chpT*. 200 ng of the purified protein 6xHis-CerM was included in the reaction. On the right, the migration of the HaeIII- PhiX174 DNA size standard is shown.

**Figure_A8.** Global expression analysis. A, MA-plot comparing the global gene expression profiles of strains Δ*cerN*/pcerM (JV17) vs AM1/pRK415 (AM1/pRK). Each gene is represented as a dot, y-axis represents log_2_ fold change (FC), x-axis represents the mean of normalized counts. The parallel lines to the x-axis represent the cut-off value to consider a gene as differentially regulated (log_2_FC ≥ 2 or ≤ -2). B, Heat map of the differentially regulated genes. Δ*cerN*/pcerM and AM1/pRK415 (AM1/pRK), expression data are depicted for the DE genes as log_2_FPKMs values. The color bar represents the expression level in log_2_ scale. At the right of the AM1 column selected genes are annotated to emphasize their regulation pattern. In green are indicated the genes of the CtrA-regulon; in black are indicated genes regulated by CerM-CerN. C, β-glucuronidase activity promoted by transcriptional fusions of the reporter gene *uidA* with three genes presumably regulated by CerM/CerN. These fusions were chromosomally placed in AM1 or Δ*cerN*/pcerM strains. Activity is expressed as pmoles of methylumbelliferone min^-1^ mg protein^-1^. Standard deviations of three independent assays are shown.

**Figure_A9.** Expression of the genes in the genetic locus encoding components for formation of RS-Mu phage. The expression of each ORF, represented as the number of fragments per kilobase million (FPKM), is shown. The orange bars represent the expression in strain AM1/pRK415, and the blue bars represent the expression in Δ*cerN*/pcerM. RSWS8N_18024 encodes the putative anti-repressor that follows the same expression pattern as the genes identified as components of RS-Mu (RSWS8N_18039-RSWS8N_18284). Interestingly, an inverse regulation is observed for RSWS8N_18014 that is annotated as a transcriptional phage repressor (COG2932) and is divergently oriented relative to the rest of the genes.

**Figure_A10.** Swimming ability of the AM1 derivative Δ*dorR*::*aadA*. A soft-agar plate containing Sistrom’s minimal medium supplemented with 0.2% casamino acids was inoculated with the indicated strains and incubated for 60 h under heterotrophic conditions. Strain EA1 (Δ*ctrA*::*aadA*) was included as a negative control.

**Figure_A11.** Logo representing the best motifs identified in the regulatory regions of the DE genes controlled by CerM/CerN using the PWM of GtaR.

**Figure_A12.** A, Electrophoretic mobility shift assay of the regulatory region of RSWS8N_14710 and CerN. Inclusion of GST-CerN (200 ng) in the reaction is represented by a green bar. AHL was included (0.66 μl) or not (-) in the binding reactions. As a positive control, a reaction containing the same DNA fragment and 200 ng of 6xHis-CerM was included (yellow bar). An asterisk indicates the migration of the CerM-DNA complex. B, Electrophoretic mobility shift assays of the regulatory region of RSWS8N_14710 and CerM (yellow bar) were conducted in the presence of increasing amounts of cell culture supernatant (CCS) obtained from strain JV14 (Δ*cerI*::*aadA*) (blue triangle). The binding reactions included GST-CerN (green bar) or were performed in its absence.
