# Supplementary material for: CerM and Its Antagonist CerN Are New Components of the Quorum Sensing System in Cereibacter sphaeroides, Signaling to the CckA/ChpT/CtrA System
Source: Microbiologyopen. 2024 Dec 18;13(6):e012. doi: 10.1002/mbo3.70012 (PMC11655674; doi:10.1002/mbo3.70012)
Supplement: Supplementary file 19 — Supporting information. [file MBO3-13-e012-s014.docx]

**File_A1_M_Supplementary_Methods.**

**Procedures to obtain the plasmids used in this work.**

**pTZ_*cerI*:** A 1714 bp region containing the *cerI* gene was amplified by PCR using the oligonucleotides CerIfor1 and CerIRV1 and cloned into the pTZ18R vector (EcoRI-/BamHI-/SmaI-) at the XbaI site.

**pTZ_cerRuPdW:** A 610 bp product corresponding to the upstream region of *cerR* was amplified using the oligonucleotides UPcerRfw and UPcerRrv, whereas a downstream product of 623 bp was obtained using the oligonucleotides DOWNcerRfw and DOWNcerRrev. These PCR products were joined through an EcoRI site designed in the oligonucleotides and cloned into pTZ18R at the XbaI site.

**pTZ_cerNuPdW:** A 662 bp product corresponding to the upstream region of *cerN* was amplified using the oligonucleotides 05815UPrv and 05815UPfw, whereas a downstream product of 711 bp was obtained using the oligonucleotides 05815DOWNrv and 05815DOWNfw. These PCR products were joined through an EcoRI site designed in the oligonucleotides and cloned into pTZ18R at the XbaI site.

**pTZ_cerMuPdW:** A 733 bp product corresponding to the upstream region of *cerM* was amplified using the oligonucleotides 05820FWup and 05820RVup, whereas a downstream product of 701 bp was obtained using the oligonucleotides 05820FWdown and 05820RVdown. These PCR products were joined through an EcoRI site designed in the oligonucleotides and cloned into pTZ18R at the XbaI site.

**pTZ_ΔcerN::Hyg-ΔcerM**: A 2,301 bp product corresponding to the downstream region of *cerN*, the Hyg resistance cassette interrupting the deleted *cerN*, the intercistronic region between *cerN* and *cerM*, and 33 bp corresponding to the coding region of *cerM* was amplified using the oligonucleotides 05815DOWNrv and 05820RVup, and chromosomal DNA from JV18 (Δ*cerN*::Hyg) as template. The downstream region of *cerM* (701 bp) was amplified by PCRS using the oligonucleotides 05820FWdown and 05820RVdown using chromosomal DNA of AM1 as template. These PCR products were joined through an EcoRI site designed in the oligonucleotides and cloned into pTZ18R at the XbaI site.

**pTZ_gtaRuPdW:** A 670 bp product corresponding to the upstream region of *gtaR* was amplified using the oligonucleotides 13935UPfw and 13935UPrv, whereas a downstream product of 704 bp was obtained using the oligonucleotides 13935DOWNfw and 13935DOWNrv. These PCR products were joined through an SacI site designed in the oligonucleotides and cloned into pTZ18R at the XbaI site.

**pTZ_RS15160uPdW**: An 822 bp product corresponding to the upstream region of RS15160 was amplified using the oligonucleotides UPrvRS15160 and UPfwRS15160, whereas a downstream product of 753 bp was obtained using the oligonucleotides DWfwRS15160 and DWrvRS15160. These PCR products were joined through an SacI site designed in the oligonucleotides and cloned into pTZ18R at the XbaI site.

**pTZ_15394uPdW**: An 800 bp product corresponding to the upstream region of 15394 was amplified using the oligonucleotides UPfw15394 and UPrv15394, whereas a downstream product of 750 bp was obtained using the oligonucleotides DWfw15394 and DWrv15394. These PCR products were joined through an EcoRI site designed in the oligonucleotides and cloned into pTZ18R at the XbaI site.

**pTZ_cerRuP-cerXdW**: A 661 bp product corresponding to the upstream region of *cerR* was amplified using the oligonucleotides UPcerRfw and UPcerRrv, whereas a downstream product of 834 bp was obtained using the oligonucleotides fwUpCerX and rvUpCerX. These PCR products were joined through an EcoRI site designed in the oligonucleotides and cloned into pTZ18R at the XbaI site.

**pTZ_cerM**: A 1001 bp fragment corresponding to *cerM* and its upstream intercistronic region was amplified using the oligonucleotides 5815UPrv and UPcerRrv and cloned into the pTZ18R vector at the SmaI site.

**pTZ_cerR**: A 1248 bp fragment corresponding to *cerR* and its upstream intercistronic region was amplified using the oligonucleotides UPcerRfw and Rv_cerR_compl and cloned into the pTZ18R vector at the SmaI site.

**pTZ_cerN**: A 597 bp fragment corresponding to *cerN* and its upstream intercistronic region was amplified using the oligonucleotides fw_compl_5815 and rv_compl_5815 and cloned into the pTZ18R vector at the SmaI site.

**pTZ_14710mut**: A 1276 bp fragment comprising 363 bp corresponding to ORF RSWS8N_14710, 622 bp of its upstream region, and 291 bp of its downstream region was amplified using the oligonucleotides Fwcro_Xba and Rvcro_Sac and cloned into the pTZ18R vector at the SmaI site. The selected plasmid released the 1276 bp fragment upon digestion with XbaI.

**pTZ_14710**: A 430 bp fragment comprising 363 bp corresponding to ORF RSWS8N_14710 and 67 bp of its upstream region was amplified using the oligonucleotides Fw14710_XbaI and Rv14710_HindIII and cloned into the pTZ18R vector at the SmaI site.

**pTZ_cerOp**: A 2614 bp fragment corresponding to *cerR*-ORF2-*cerI* and the upstream intercistronic region of *cerR* was amplified using the oligonucleotides UPcerRfw and Rv_cerR_compl and cloned into the pTZ18R vector at the SmaI site.

**pTZ_cerMpET**: An 827 bp fragment corresponding to *cerM* without its start codon was amplified using the oligonucleotides Fw_5820_BamH1 and UPcerRrv and cloned into the pTZ18R vector at the SmaI site.

**pTZ_cerNpGEX**: A 491 bp fragment corresponding to *cerN* without its start codon was amplified using the oligonucleotides 5815_rv_salI_pGEX and 5815_fw_ecoRI_pGEX and cloned into the pTZ18R vector at the SmaI site.

**pJQ_ΔcerI::aadA**: The plasmid pTZ_*cerI* was digested with EcoRI and BamHI, resulting in the deletion of a 247 bp region corresponding to the coding sequence of *cerI*. The ends were repaired with T4 DNA polymerase and interrupted with the *aadA* cassette, which confers resistance to the antibiotic spectinomycin. The Δ*cerI*::*aadA* fragment was released from pTZ18R with XbaI and cloned into the pJQ200mp18 vector in the XbaI site.

**pJQ_ΔcerI::Rif**: The pTZ_*cerI* was digested with EcoRI and BamHI, resulting in the deletion of a 247 bp region of the coding sequence of *cerI*. The ends were repaired with T4 DNA polymerase and ligated with the 678 bp Rif cassette, which confers resistance to the antibiotic rifampicin. The Δ*cerI*::Rif fragment was released from pTZ18R with XbaI and cloned into the pJQ200mp18 vector.

**pJQ_ΔcerR::aadA**: The *aadA* cassette was cloned into pTZ_cerRuPdW previously digested with EcoRI. The DNA fragment carrying Δ*cerR::aadA* was excised from pTZ_ΔcerR::*aadA* by digestion with XbaI and subsequently subcloned into the XbaI site of pJQ200mp18 vector.

**pJQ_ΔcerN::aadA**: The *aadA* cassette was cloned into pTZ_cerNuPdW previously digested with EcoRI. The DNA fragment carrying Δ*cerN*::*aadA* was excised from pTZ18R by digestion with XbaI and subsequently subcloned into the XbaI site of pJQ200mp18 vector.

**pJQ_ΔcerN::Hyg**: A DNA fragment of 1390 bp corresponding to the Hyg cassette was cloned into pTZ_cerNuPdW previously digested with EcoRI. The DNA fragment corresponding to Δ*cerN*::Hyg was released from pTZ18R with XbaI and subcloned into the XbaI site of pJQ200mp18 vector.

**pJQ_ΔcerM::aadA**: The *aadA* cassette was cloned into pTZ_cerMuPdW previously digested with EcoRI. The DNA fragment corresponding to Δ*cerM*::*aadA* was released from the pTZ18R with XbaI and subcloned into the XbaI site of pJQ200mp18 vector.

**pJQ_Δ*cerN*::Hyg-Δ*cerM***: The fragment Δ*cerN*::Hyg-Δ*cerM*, was released with XbaI from the pTZ_ ΔcerN::Hyg-ΔcerM and subcloned into the XbaI site of pJQ200mp18 vector.

**pJQ*_ΔgtaR::aadA*:** The *aadA* cassette was cloned into pTZ_gtaRuPdW previously digested with SacI. The DNA fragment corresponding to *ΔgtaR:aadA* was released from pTZ18R with XbaI and subcloned into the XbaI site of pJQ200mp18 vector.

**pJQ_ΔgtaR::uidA-Hyg**: The fragment containing the *uidA*-Hyg cassette was generated by joining the *uidA* fragment from pBBR1gus and the Hyg resistance cassette from pIJ963. The resulting fragment was cloned into pTZ_gtaRuPdW**,** which had been previously digested with SacI and end-repaired using T4 DNA polymerase. After selecting the correct orientation of the reporter gene, this plasmid was digested with XbaI to obtain the Δ*gtaR::uidA*-Hyg fragment and subcloned into the XbaI site of pJQ200mp18 vector.

**pJQ_ΔRS15160::uidA-Hyg**: The *uidA*-Hyg cassette was cloned into pTZ_RS15160uPdW previously digested with SacI. The DNA fragment corresponding to ΔRS15160::*uidA*-Hyg was released from the pTZ18R with XbaI and subcloned into the XbaI site of pJQ200mp18 vector.

**pJQ_Δ15394::uidA-Hyg**: The *uidA*-Hyg cassette was cloned into pTZ_15394uPdW previously digested with EcoRI. The DNA fragment corresponding to Δ*15394*::*uidA*-Hyg was released from the pTZ18R with XbaI and subcloned into the XbaI site of pJQ200mp18 vector.

**pJQ_14710::aadA:** The *aadA* cassette was cloned into pTZ_14710mut, which had been previously digested with SalI and end-repaired with T4 DNA polymerase. The DNA fragment carrying 14710::*aadA* was excised from pTZ_14710::*aadA* by digestion with XbaI and subsequently subcloned into the XbaI site of the pJQ200mp18 vector.

**pJQ_ΔcerOp::aadA**: The *aadA* cassette was cloned into pTZ_cerRuP-cerXdW previously digested with EcoRI. The DNA fragment corresponding to Δ*cerOp*:*aadA* was released from pTZ18R with XbaI and subcloned into the XbaI site of pJQ200mp18 vector.

**pcerM (pRK415_cerM)**: The fragment carrying *cerM* was excised from pTZ_cerM with EcoRI and subcloned into pRK415, previously digested with EcoRI. In this plasmid, *cerM* is oriented opposite to the *plac* promoter present in pRK415.

**pcerN (pRK415_cerN)**: The fragment carrying *cerN* was released with EcoRI and XbaI from the pTZ_cerN and subcloned into pRK415, which had been previously digested with EcoRI and XbaI. In this plasmid, *cerN* is oriented opposite to the *plac* promoter present in pRK415.

**pcerR (pRK415_cerR)**: The fragment carrying *cerR* was released from the pTZ_cerR with EcoRI and XbaI and subcloned into pRK415, previously digested with EcoRI and XbaI. In this plasmid, *cerR* is oriented in the same direction as the *plac* promoter present in pRK415.

**p14710 (pRK415_14710)**: The fragment carrying ORF RSWS8N_14710 was excised from pTZ_14710 with XbaI and HindIII, and subcloned into pRK415, previously digested with XbaI and HindIII.

**pcerI (pRK415_cerI)**: The XbaI fragment obtained from pTZ_*cerI* plasmid was subcloned into pRK415, which had been previously digested with XbaI. In this plasmid, *cerI* is oriented in opposite direction to the *plac* promoter present in pRK415.

**pcerOp (pRK415_cerOp)**: The fragment carrying *cerR-*ORF2*-cerI* was released from the pTZ_cerOp with XbaI and subcloned into pRK415, previously digested with XbaI. In this plasmid, *cerR-*ORF2*-cerI* is oriented in opposite direction to the *plac* promoter present in pRK415.

**pcerOpΔcerR (pRK_cerOpΔcerR)**: The plasmid pTZ_cerOp was used as a template for a reverse PCR reaction with the oligonucleotides UPcerRrv and DOWNcerRfw. After self-ligation, the presence of a deletion of 511 bp in the coding region of *cerR* was verified. The fragment carrying Δ*cerR-*ORF2*-cerI* was released with XbaI from the pTZ18R and subcloned into pRK415, which was previously digested with XbaI. In this plasmid, the Δ*cerR-*ORF2*-cerI* is oriented in opposite direction to the *plac* promoter present in pRK415.

**pcerOpΔorf-2 (pRK415_cerOpΔorf-2)**: The plasmid pTZ_cerOp was used as a template for a reverse PCR reaction with the oligonucleotides cerAmutEcoR1 and Rv_cerR_compl. After self-ligation, the presence of a deletion that encompasses from -19 to +3 of the coding region of ORF-2, was verified. The fragment carrying *cerR-*ΔORF2*-cerI* was released with XbaI from the pTZ18R_ pcerOpΔorf-2 and subcloned into pRK415, which was previously digested with XbaI. In this plasmid, *cerR-*ΔORF2*-cerI* is oriented in opposite direction to the *plac* promoter present in pRK415.

**pET_6xH-cerM (pET28a_6xHis-CerM)**: The DNA fragment carrying *cerM* was excised from pTZ_cerMpET with BamHI and EcoRI and subcloned into pET28a, which had been previously digested with the same enzymes.

**pGEX_cerN (pGEX-4T-2_GST-CerN)**: The fragment carrying *cerM* was released from pTZ_cerNpGEX with EcoRI and SalI and subcloned into pGEX-4T-2, which had been previously digested with the same enzymes.

**pcerMBS1::lacZ (pRS551_cerMBS1::lacZ)**: 10 µM of the oligonucleotides FW_artifprom_CerM and RV_artifprom_CerM were annealed through a temperature gradient from 98 °C to 12 °C. The resulting product was ligated into the pRS551 plasmid, which had been previously digested with EcoRI and BamHI to produce the transcriptional fusion *cerM*BS1::*lacZ*.

**pGEX_cerM (pGEX-4T-2_6xHisCerM)**: A 1,188 bp product containing the 6xHis-cerM fusion was PCR amplified from the vector pET28a_6xHis-cerM using the oligonucleotides T7promBglII and T7terBglII. The resulting product was cloned into the pGEX-4T-2 vector, which had been previously digested with EagI. In this plasmid, 6xHisCerM is oriented in the same direction as the *ptac* promoter present in pGEX-4T-2.

**pGEX_cerNM (pGEX-4T-2_GST-CerN - 6xHis-CerM)**: A PCR product of 1,188 bp containing the 6xHis-cerM was obtained from pET28a_6xHis-cerM using the oligonucleotides T7promBglII and T7terBglII. The resulting product was cloned into the pGEX_cerN vector, which had been previously digested with EagI. In this plasmid, 6xHisCerM is oriented in the same direction as the *ptac* promoter present in pGEX-4T-2.
